# Supplementary material for: Pyrrolizine/Indolizine-NSAID Hybrids: Design, Synthesis, Biological Evaluation, and Molecular Docking Studies
Source: Molecules. 2021 Oct 30;26(21):6582. doi: 10.3390/molecules26216582 (PMC8588198; doi:10.3390/molecules26216582)
Supplement: Supplementary file 1 [file molecules-26-06582-s001.zip › molecules-1436408-supplementary.pdf]

Pyrrolizine/indolizine-NSAID hybrids: design, synthesis, biological evaluation, and molecular docking studies

Mohammed A. S. Abourehab[1], Alaa M. Alqahtani[2,*], Faisal A. Almalki[2], Dana M. Zaher[3], Ashraf N. Abdalla[4,5], Ahmed M. Gouda[6,*] Eman A. M. Beshr[7]

[1]Department of Pharmaceutics, Faculty of Pharmacy, Umm Al-Qura University, Makkah 21955, Saudi Arabia

[2]Department of Pharmaceutical Chemistry, Faculty of Pharmacy, Umm Al-Qura University, Makkah 21955, Saudi Arabia

[3]Sharjah Institute for Medical Research, University of Sharjah, Sharjah 27272, United Arab Emirates

[4]Department of Pharmacology and Toxicology, Faculty of Pharmacy, Umm Al-Qura University, Makkah 21955, Saudi Arabia

[5]Department of Pharmacology, Medicinal and Aromatic plants research institute, National center for research, Khartoum 2404, Sudan

[6]Medicinal chemistry department, Faculty of pharmacy, Beni-Suef University, Beni-Suef 62514, Egypt

[7]Department of Medicinal Chemistry, Faculty of Pharmacy, Minia University, Minia 61519, Egypt

---

***Correspondence:**

***Ahmed M. Gouda:**

Department of Medicinal Chemistry, Faculty of Pharmacy, Beni-Suef University, Beni-Suef 62514, Egypt.
Scopus ID: 26321547200
ORCID: 0000-0003-4527-8885
Tel.: (002)-01126897483
Fax: (002)-082-2162133
E-mail address: ahmed.gouda@pharm.bsu.edu.eg or amsaid@uqu.edu.sa

***Mohammed A. S. Abourehab[1], Ph.D.**

Department of Pharmaceutics, Faculty of Pharmacy, Umm Al-Qura University, Makkah 21955, Saudi Arabia.

E-mail address: maabourehab@uqu.edu.sa

Supplementary materials

IR Spectra

Infrared spectra (IR) were done using BRUKER TENSOR 37 spectrophotometer and absorptions were expressed in wave number (cm^{-1}) using KBr disc.

Fig. S1. IR spectrum of compound **8a**

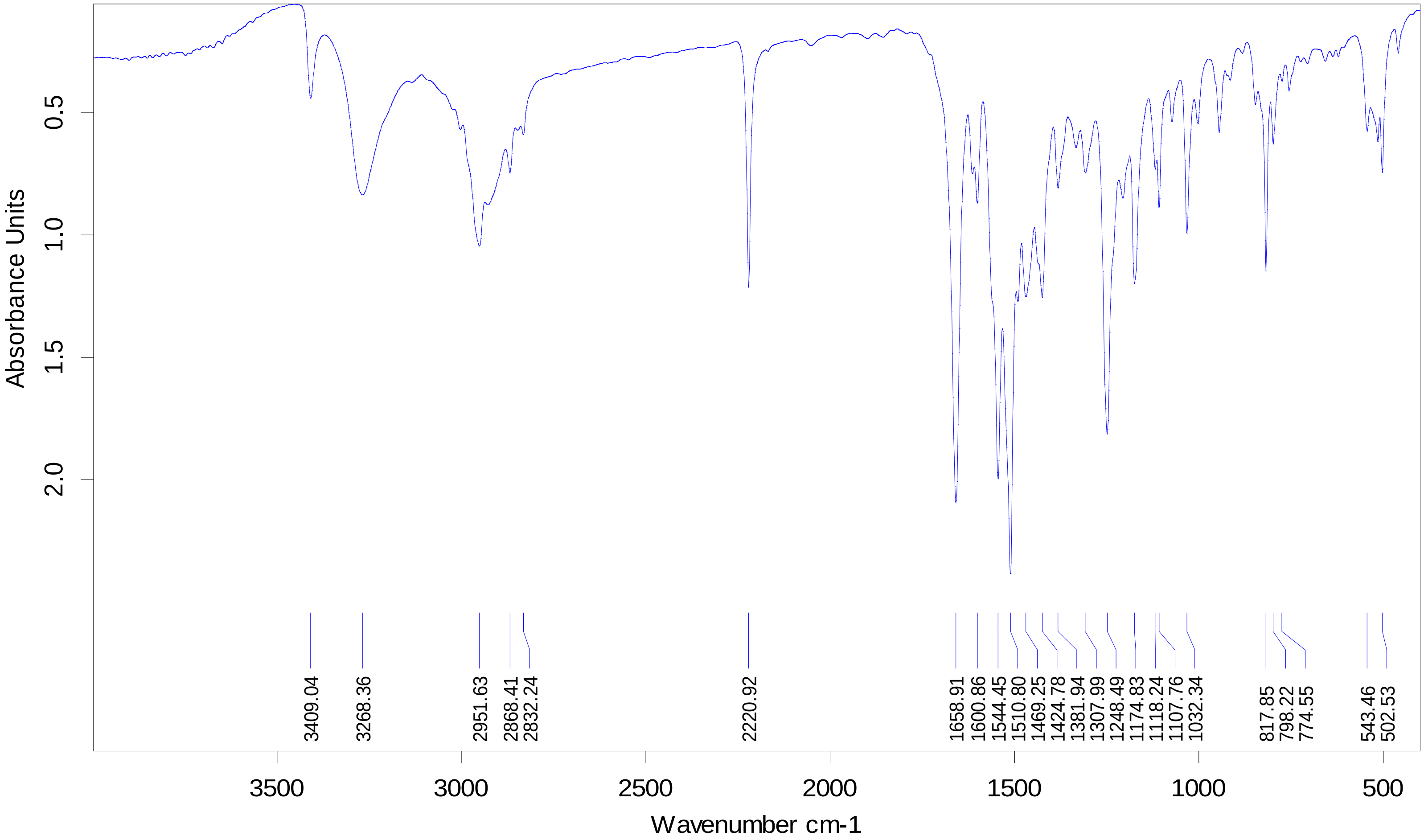

C:\Program Files\OPUS_65\MEAS\Protein.290	Protein	AquaSpec	04/02/2018

Page 1/1

Fig. S2. IR spectrum of compound **8b**

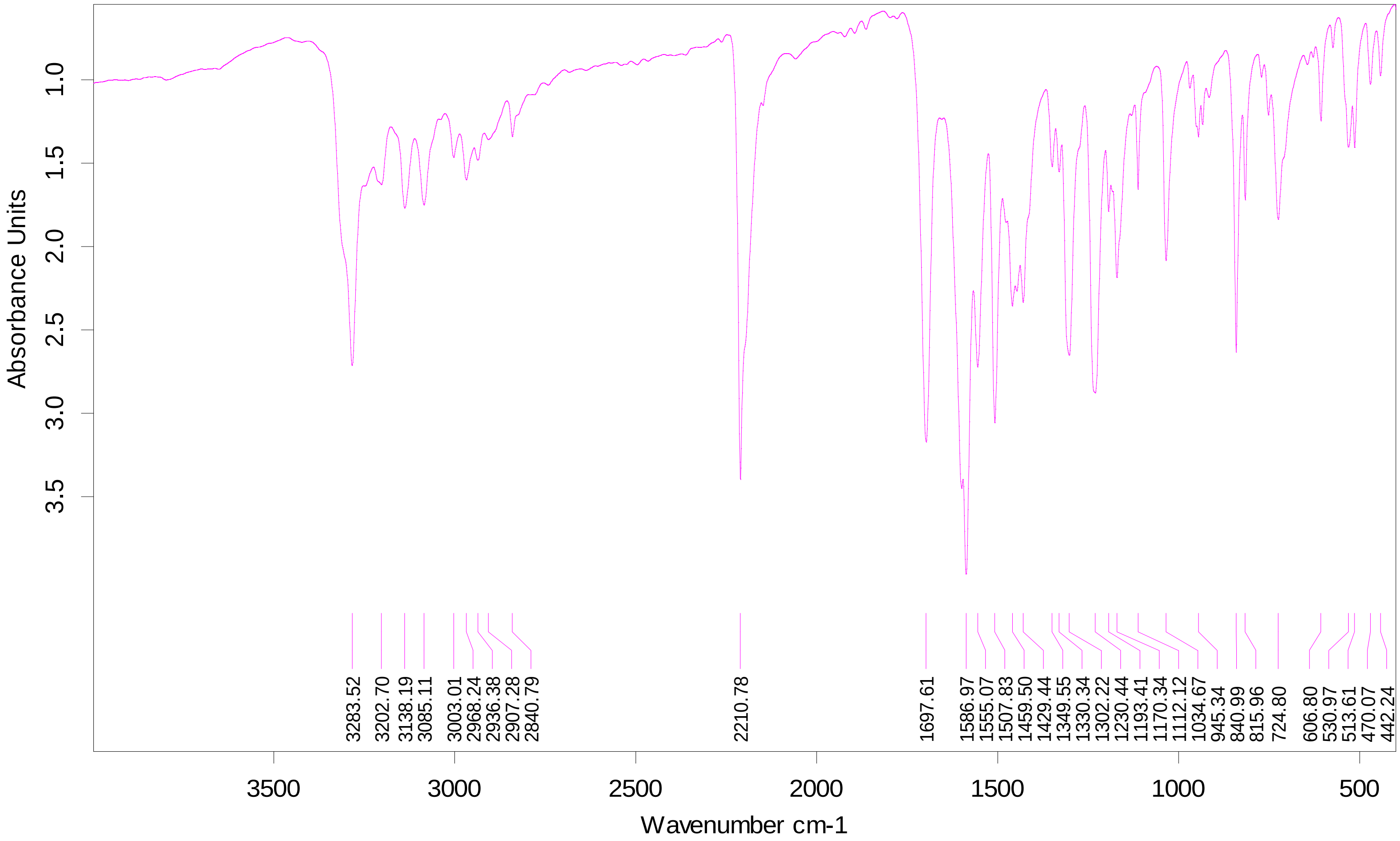

C:\Program Files\OPUS_65\MEAS\Protein.292 Protein AquaSpec	04/02/2018

Page 1/1

Fig. S3. IR spectrum of compound **8c**

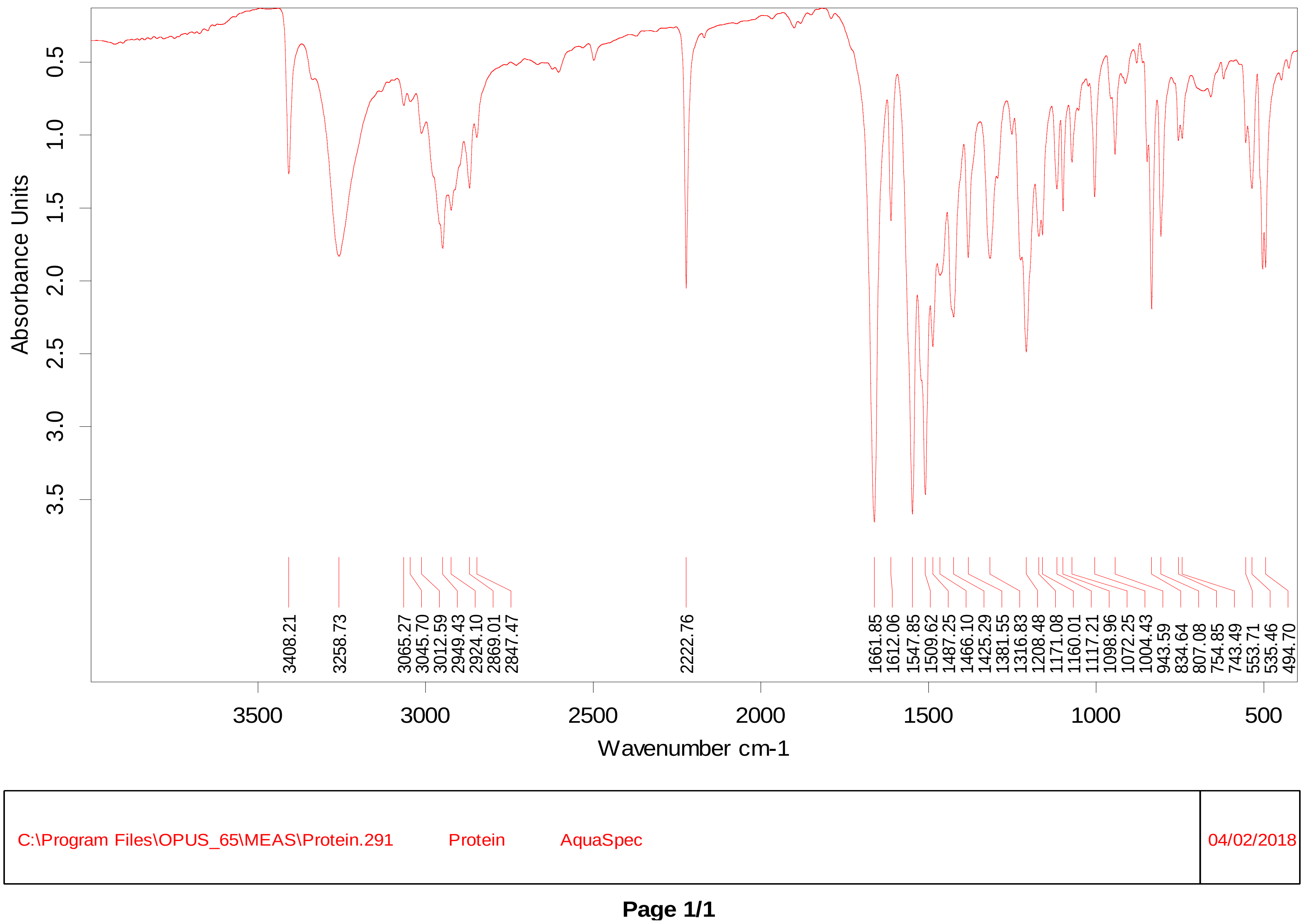

Fig. S4. IR spectrum of compound **8d**

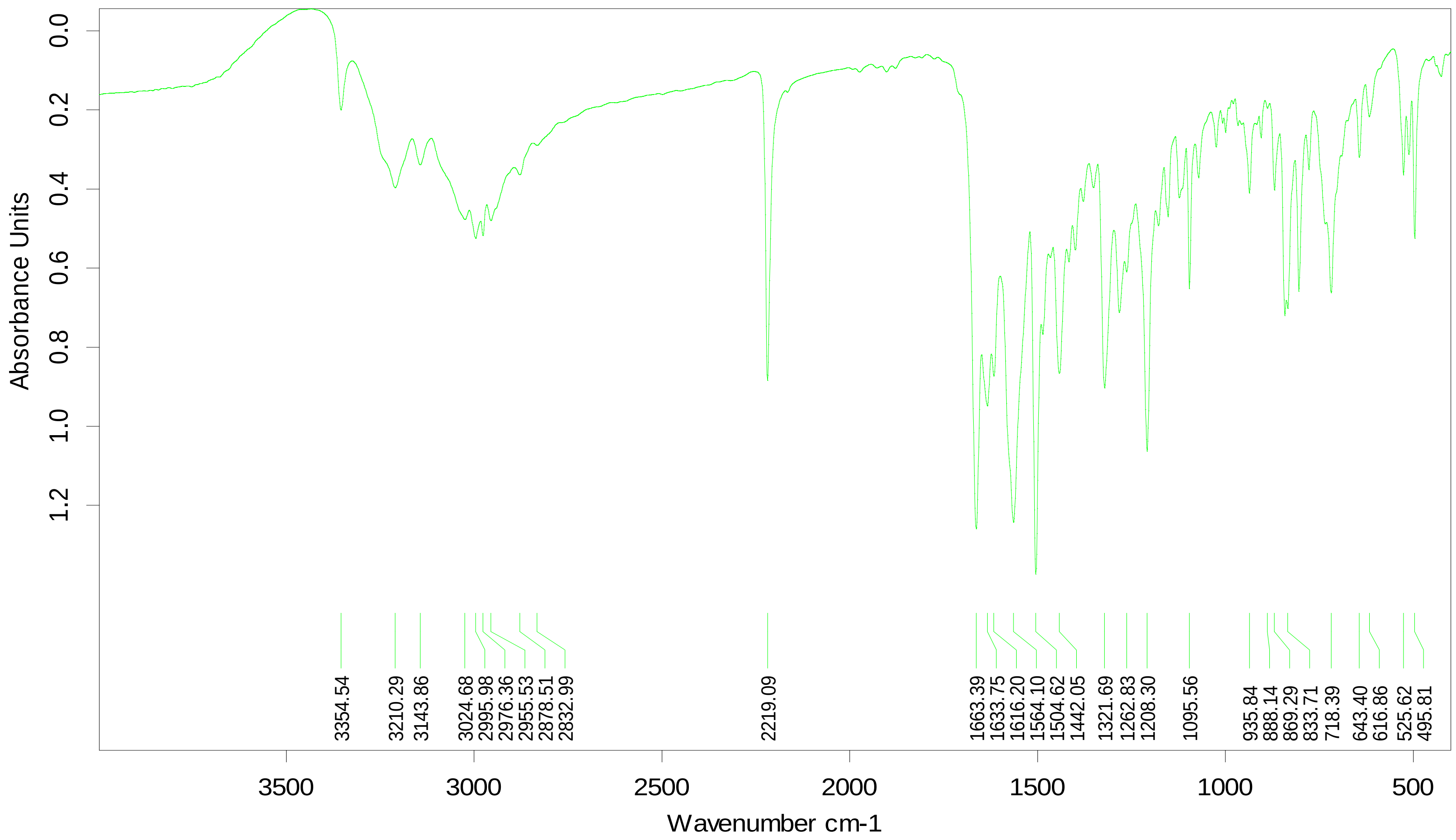

C:\Program Files\OPUS_65\MEAS\Protein.293 Protein AquaSpec	04/02/2018

Page 1/1

Fig. S5. IR spectrum of compound **8e**

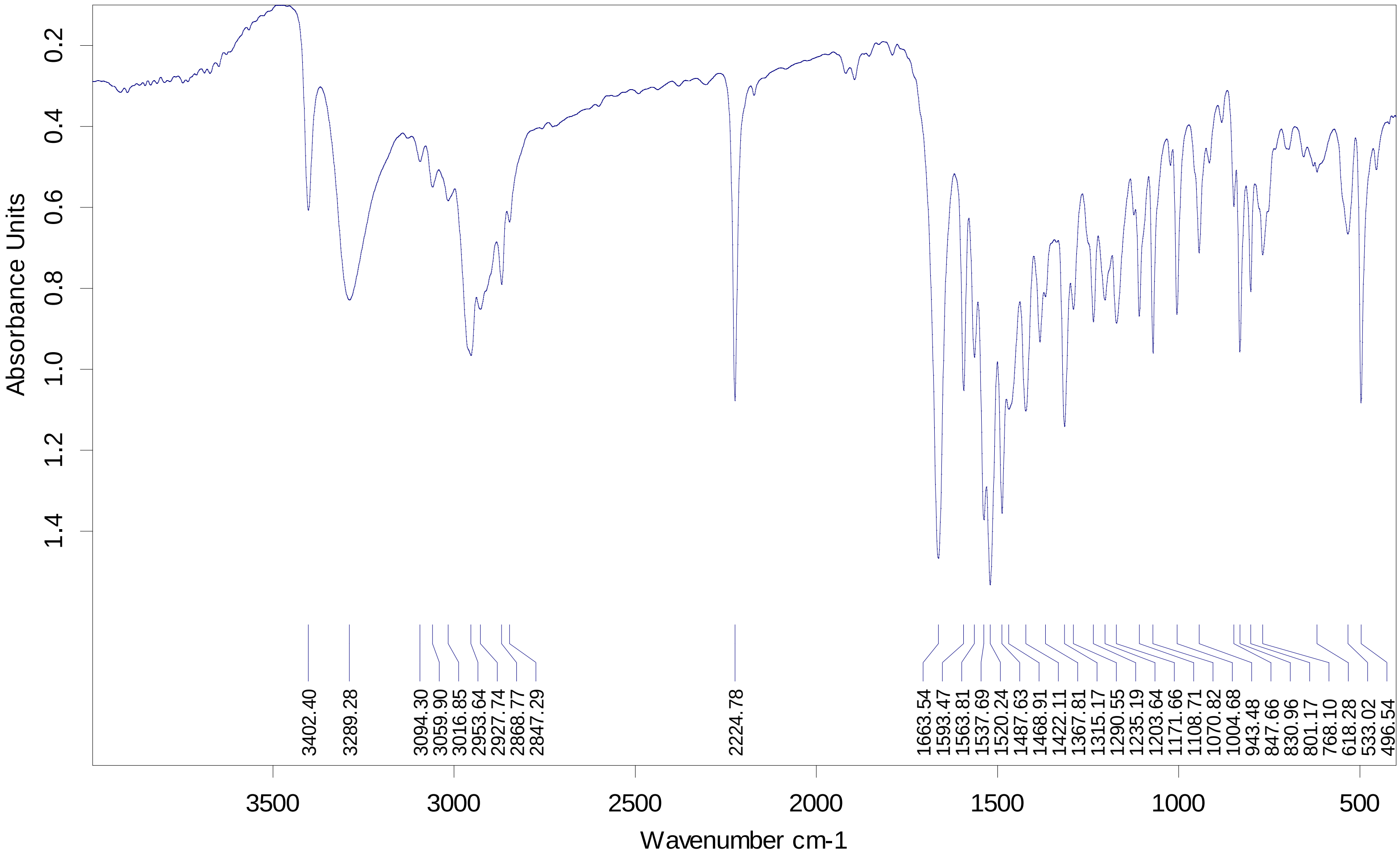

C:\Program Files\OPUS_65\MEAS\Protein.298 Protein AquaSpec	04/02/2018

Page 1/1

Fig. S6. IR spectrum of compound **8f**

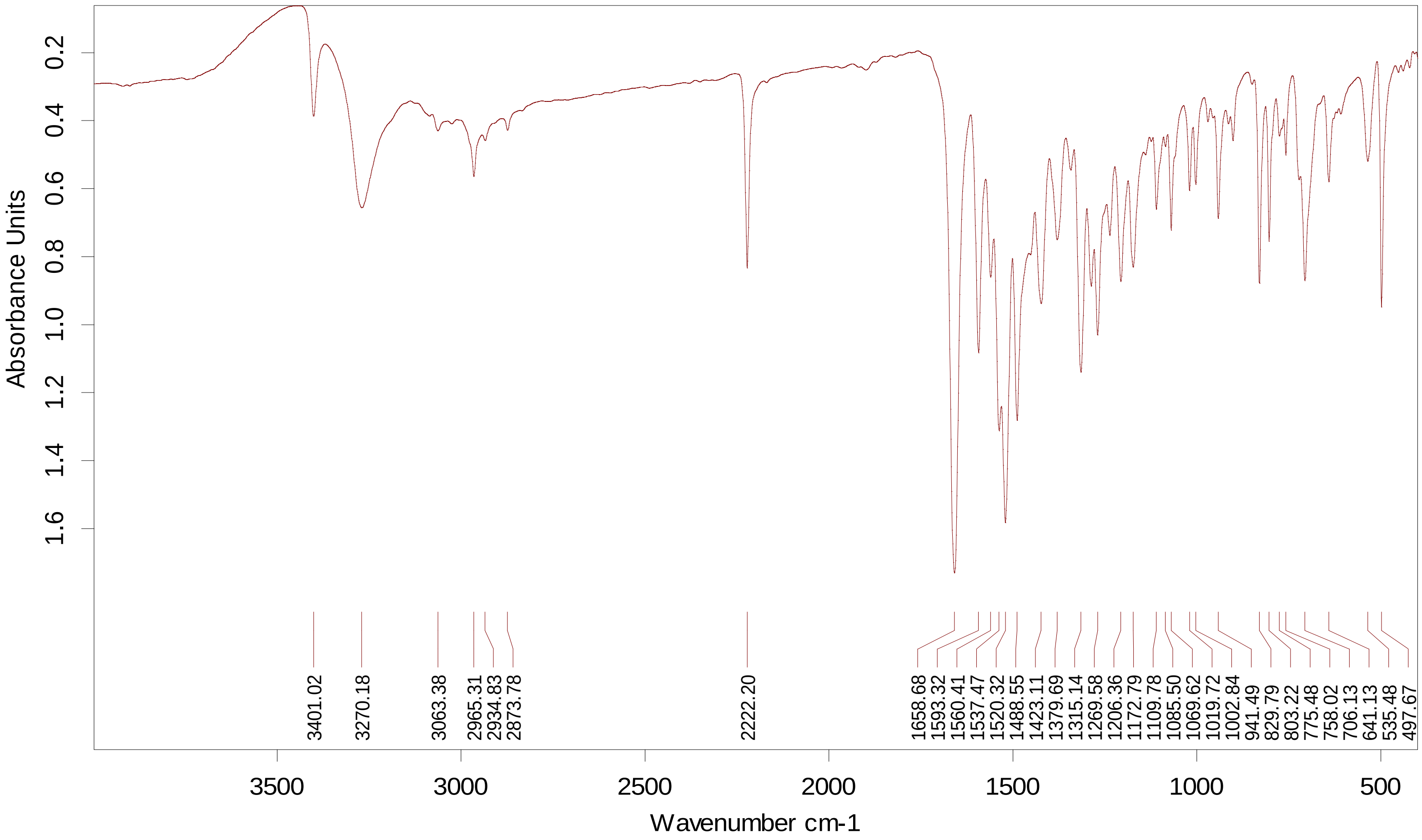

C:\Program Files\OPUS_65\MEAS\Protein.299	Protein	AquaSpec	04/02/2018

Page 1/1

Fig. S7. IR spectrum of compound **8g**

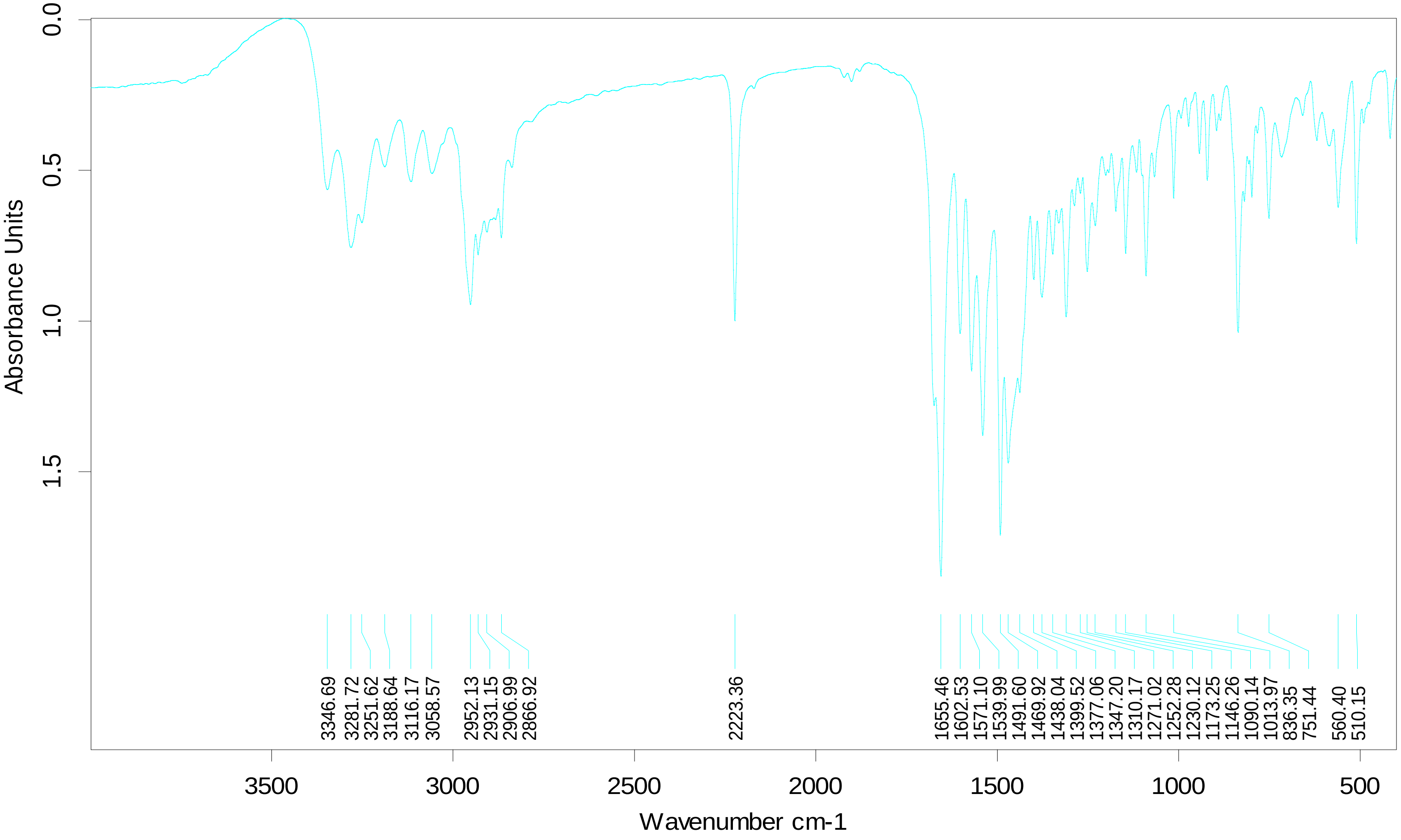

C:\Program Files\OPUS_65\MEAS\Protein.294	Protein	AquaSpec	04/02/2018

Page 1/1

Fig. S8. IR spectrum of compound **8h**

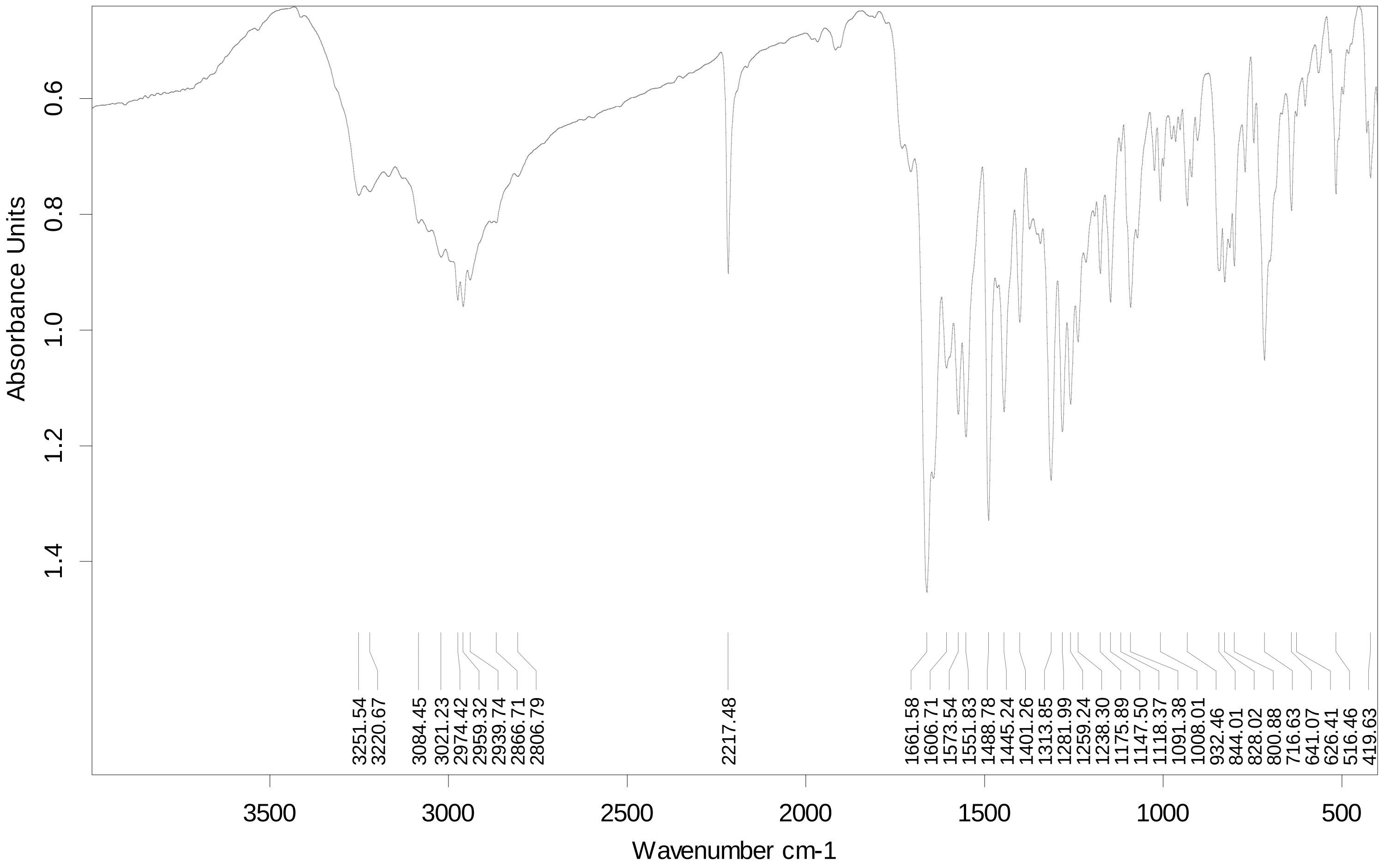

C:\Program Files\OPUS_65\MEAS\Protein.297	Protein	AquaSpec		04/02/2018

Fig. S9. IR spectrum of compound **8i**

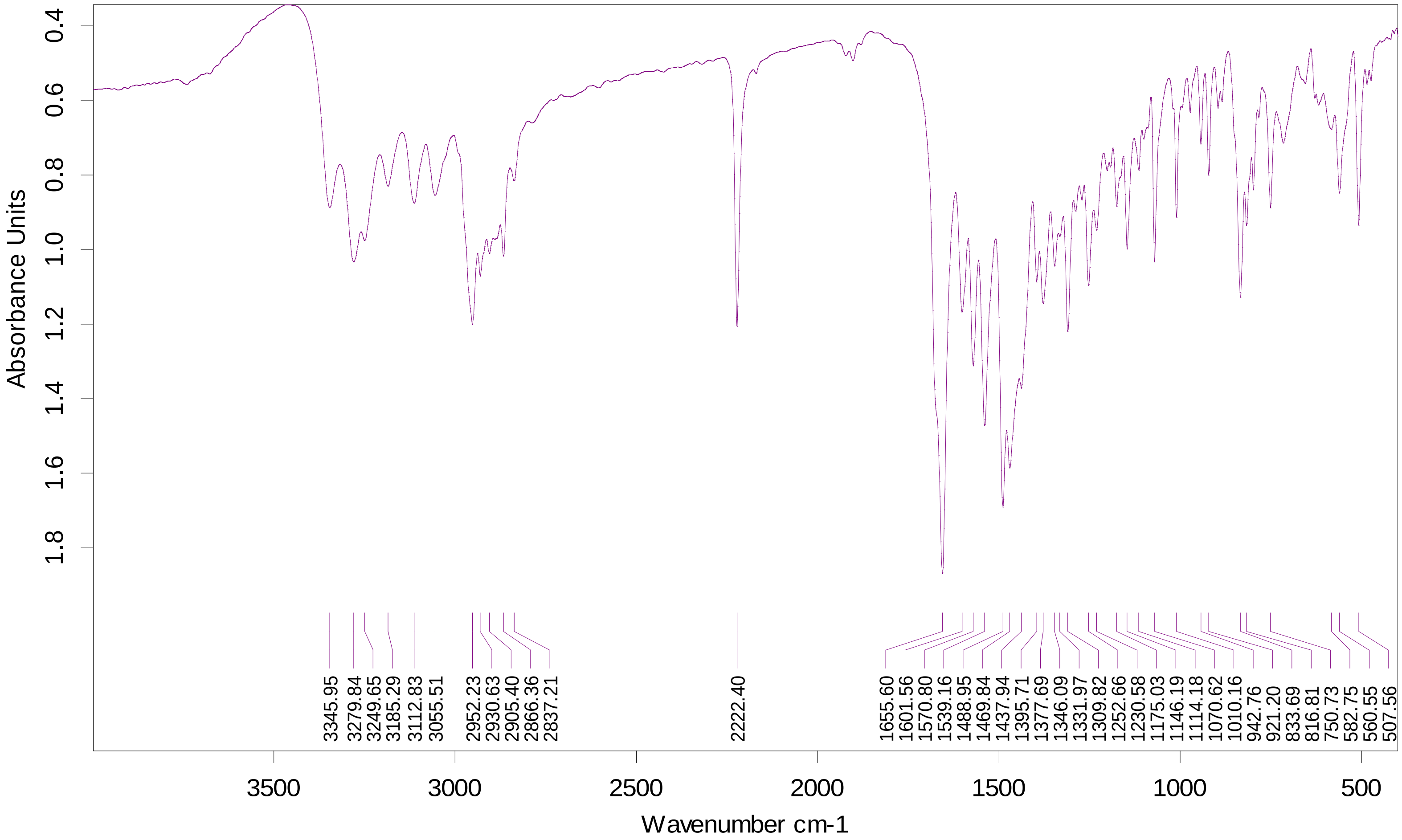

C:\Program Files\OPUS_65\MEAS\Protein.300	Protein	AquaSpec		04/02/2018

Page 1/1

^{1}H-NMR, ^{13}C-NMR & DEPT C^{135} Spectra

- ^{1}H-NMR spectra were recorded on a BRUKER AVANCE III spectrometer (at the faculty of pharmacy, Umm Al-Qura University) at 500 MHz in the specified solvent, chemical shifts were reported on the δ scale and were related to that of the solvent and J values are given in Hz
- ^{13}C NMR and DEPT135 spectra were obtained on a BRUKER AVANCE III at 125 MHz (at the faculty of pharmacy, Umm Al-Qura University) and 100 MHz (at the faculty of pharmacy, Beni-Suef University).

Fig. S10. ^{1}H-NMR ($CDCl_3$, 500 MHz, δ ppm) spectrum of compound **8a**

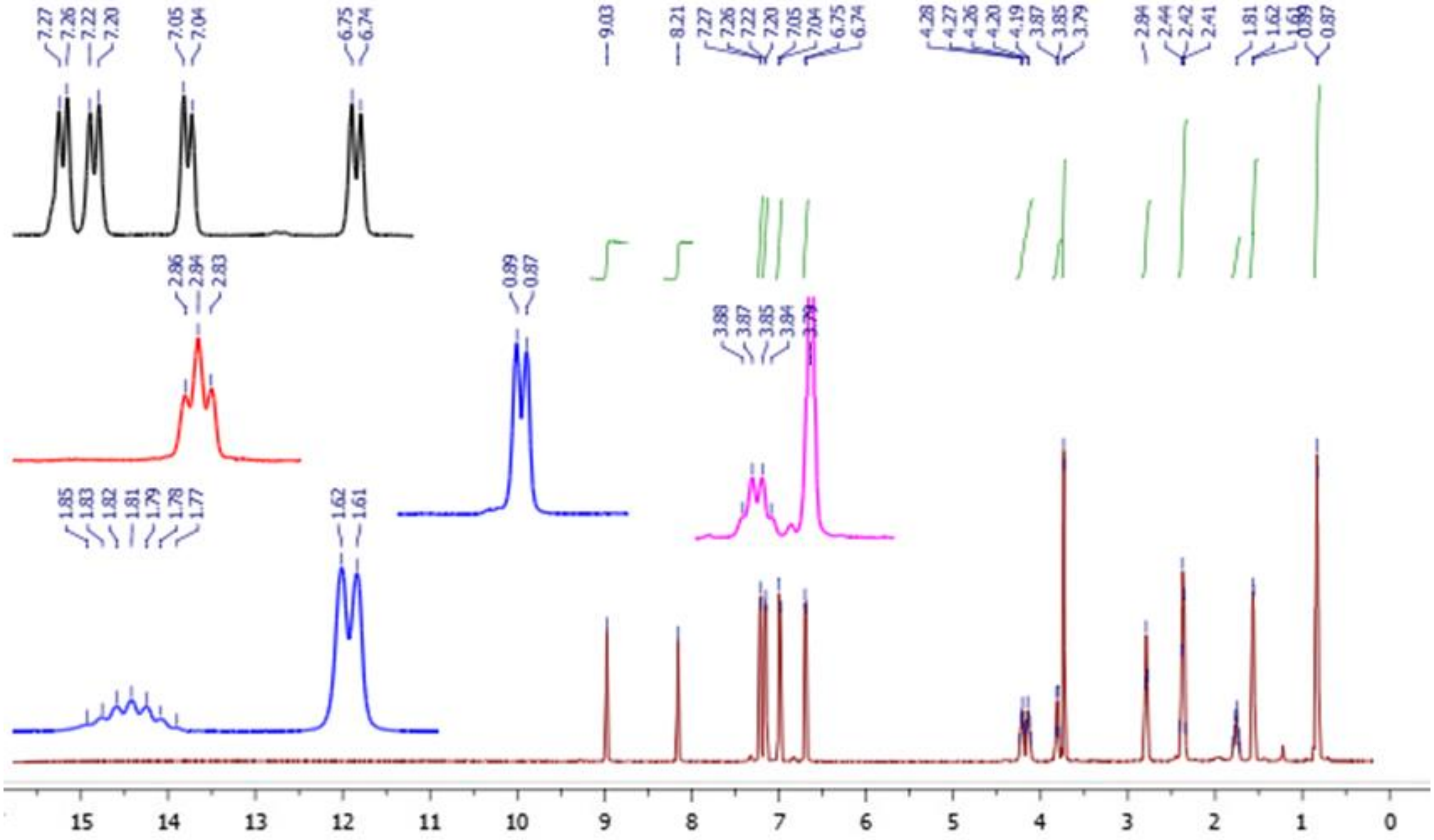

Fig. S11. ^{1}H-NMR ($CDCl_3$, 500 MHz, δ ppm) spectrum of compound **8a**

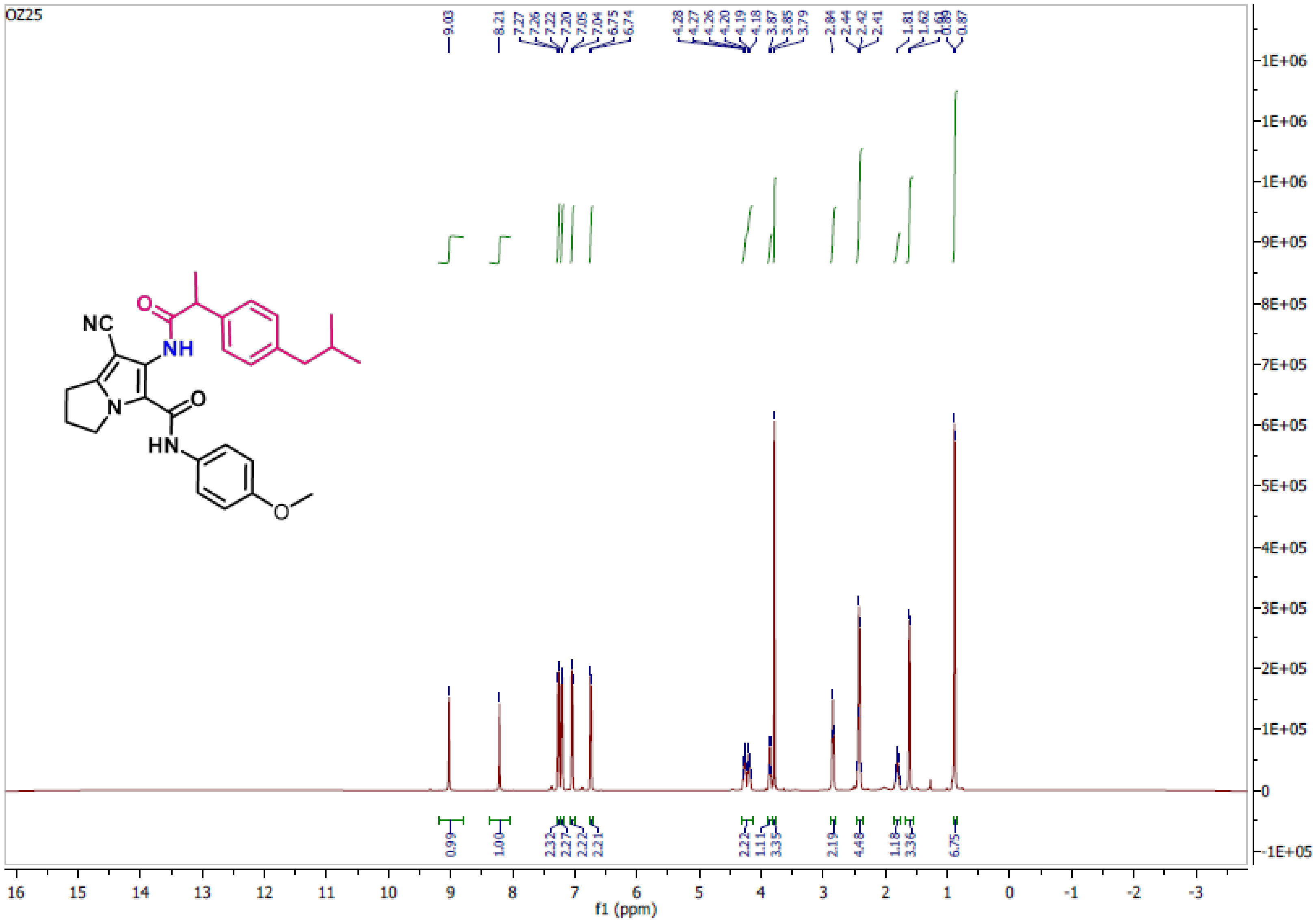

Fig. S12. ^{1}H-NMR ($CDCl_3$, 500 MHz, δ ppm) spectrum of compound **8a** **(zoom on aliphatic Hs)**

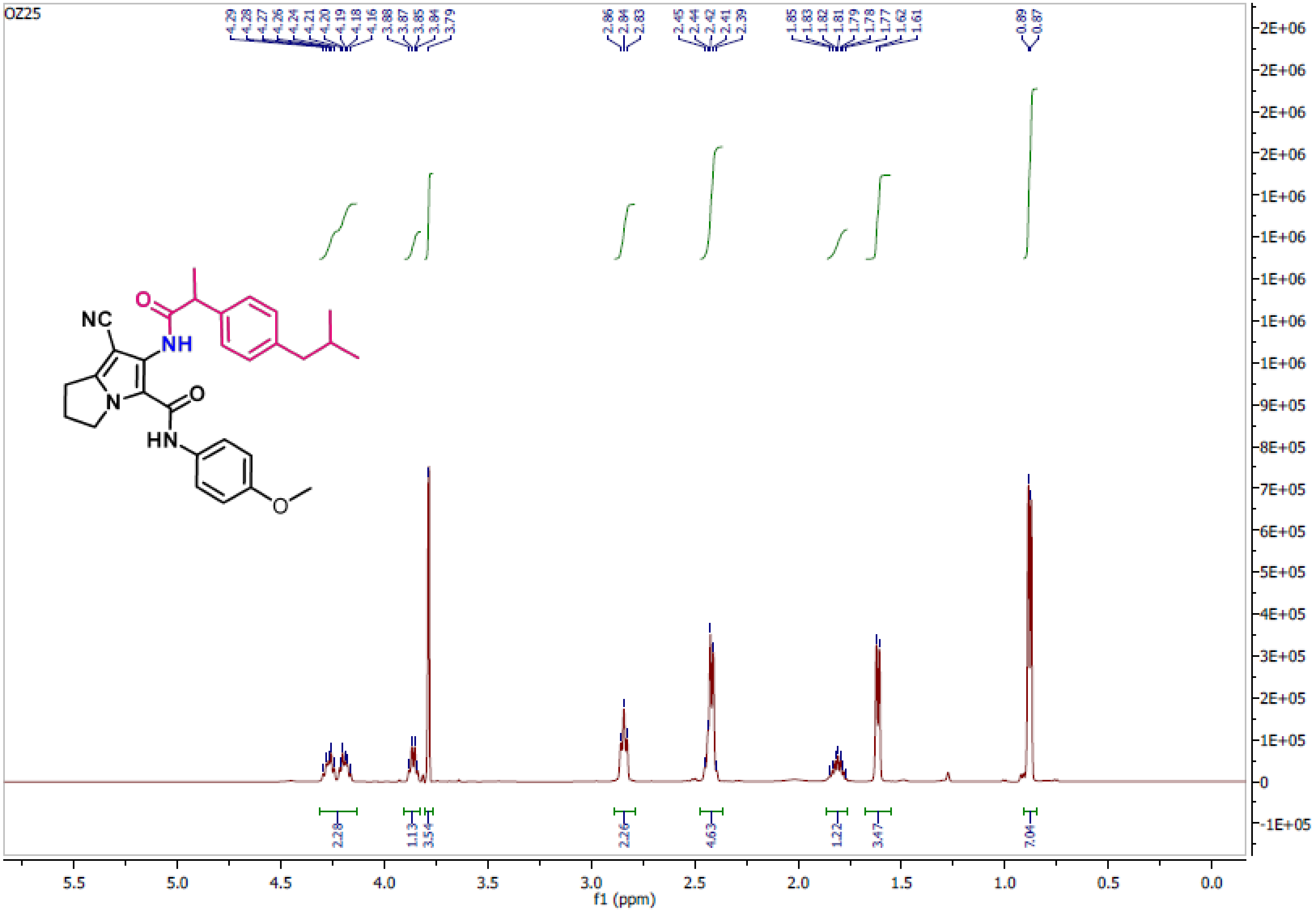

Supplementary materials

Fig. S13. ^{1}H-NMR ($CDCl_3$, 500 MHz, δ ppm) spectrum of compound **8a** **(zoom on aromatic Hs)**

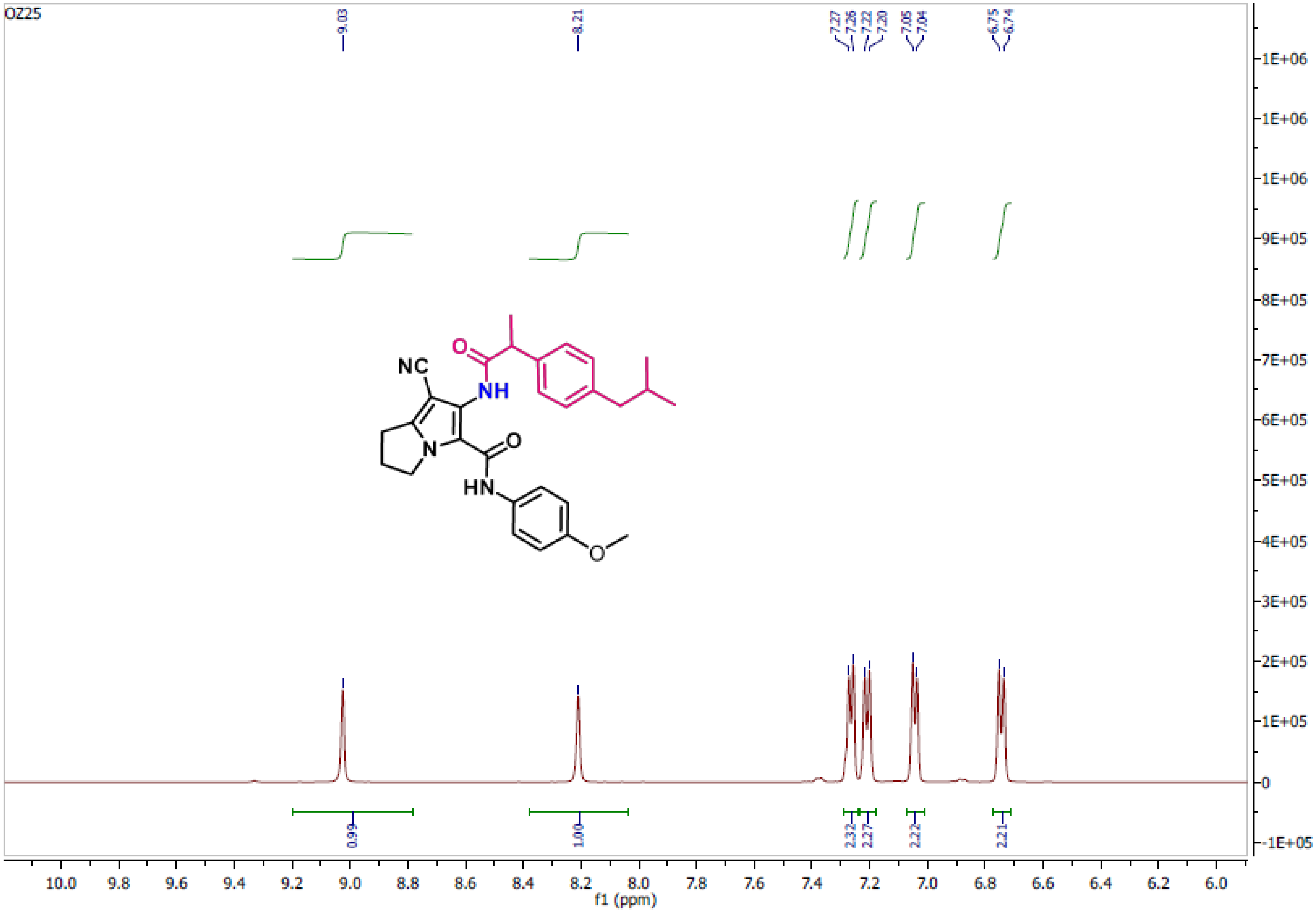

Fig. S14. ^{13}C-NMR ($CDCl_3$, 125 MHz, δ ppm) spectrum of compound **8a**

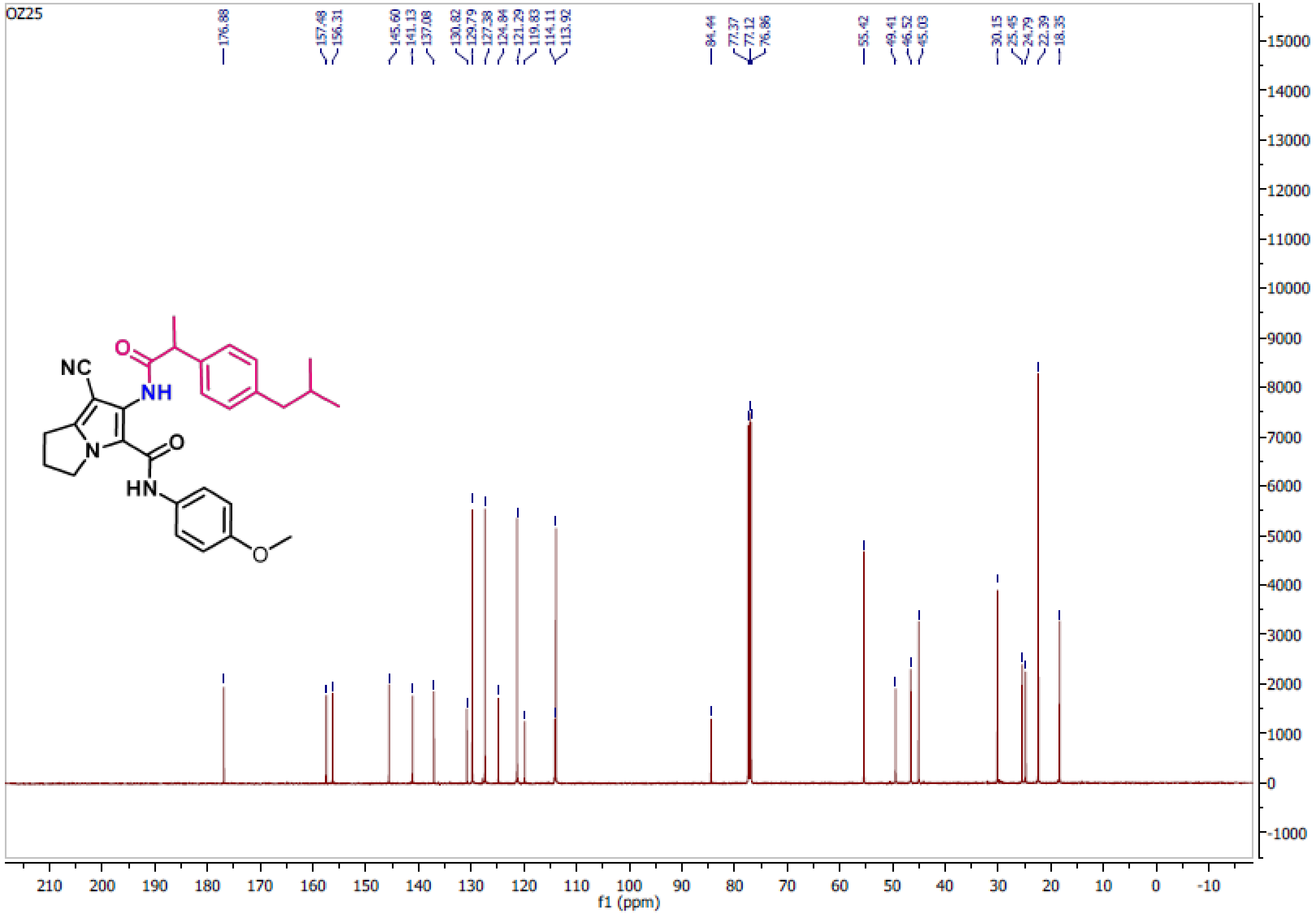

Fig. S15. ^{13}C-NMR ($CDCl_3$, 125 MHz, δ ppm) spectrum of compound **8a** **(zoom on aliphatic Cs)**

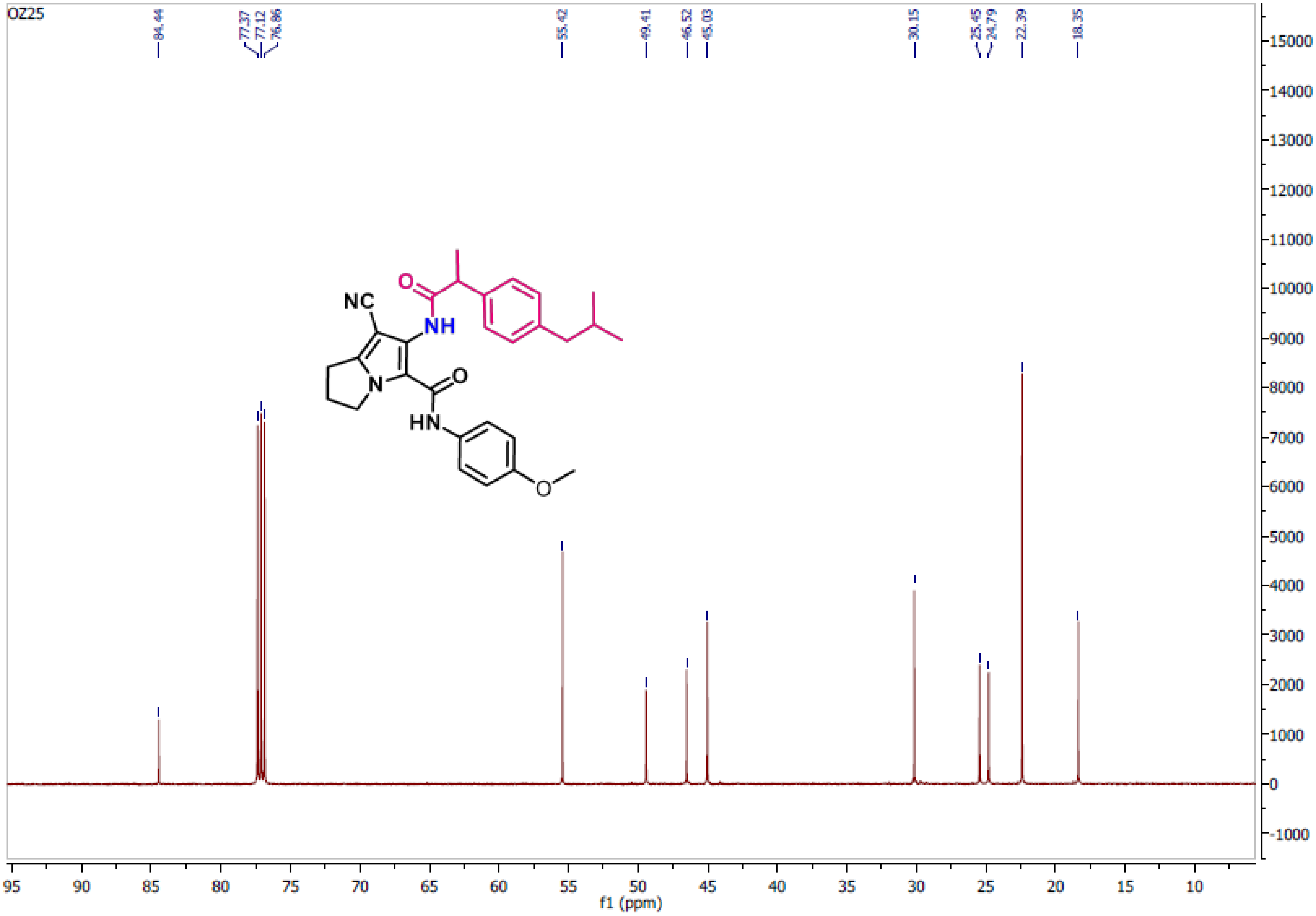

Fig. S16. ^{13}C-NMR ($CDCl_3$, 125 MHz, δ ppm) spectrum of compound **8a** **(zoom on aromatic Cs)**

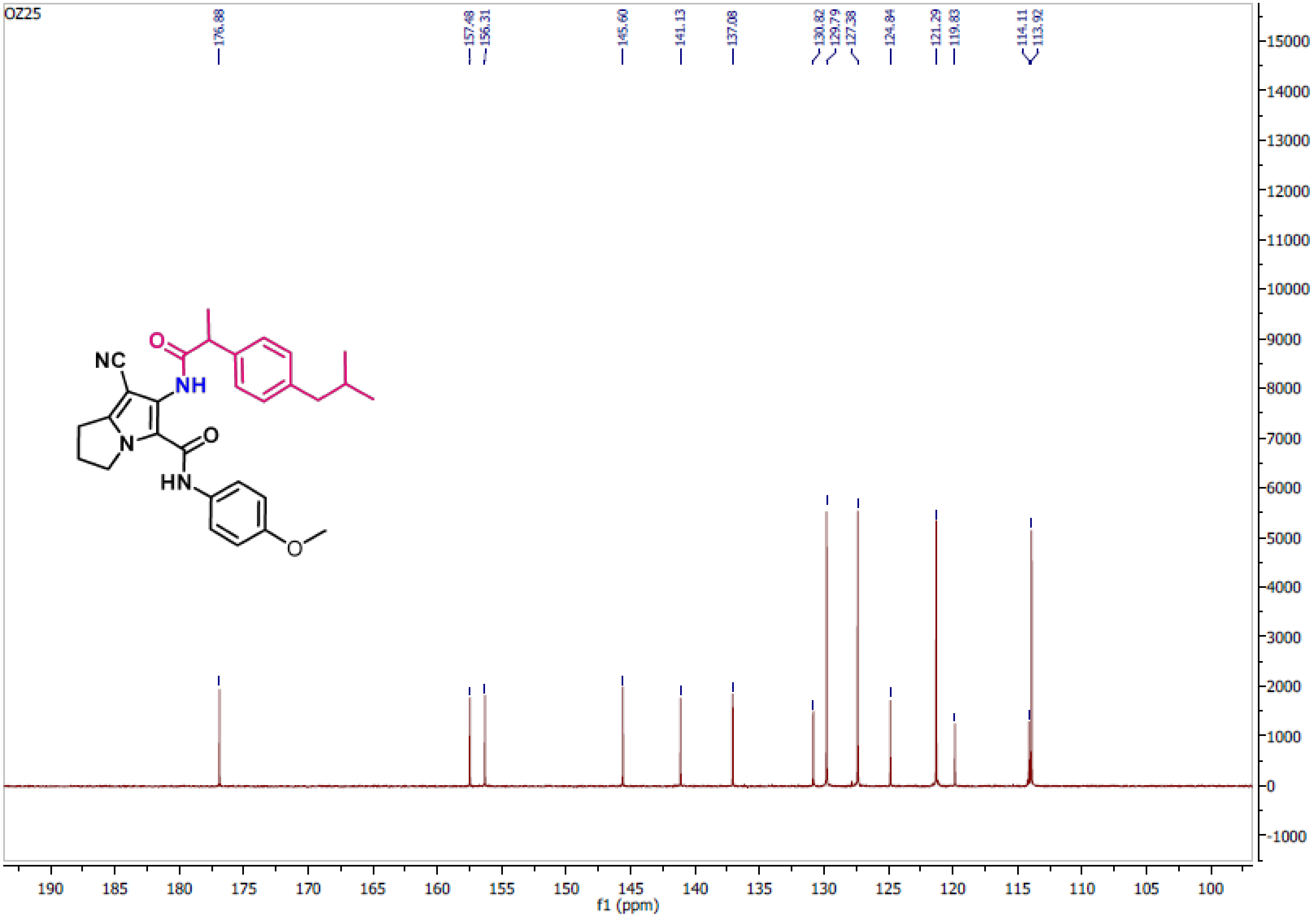

Fig. S17. DEPT C135 ($CDCl_3$, 125 MHz, δ ppm) of compound **8a**

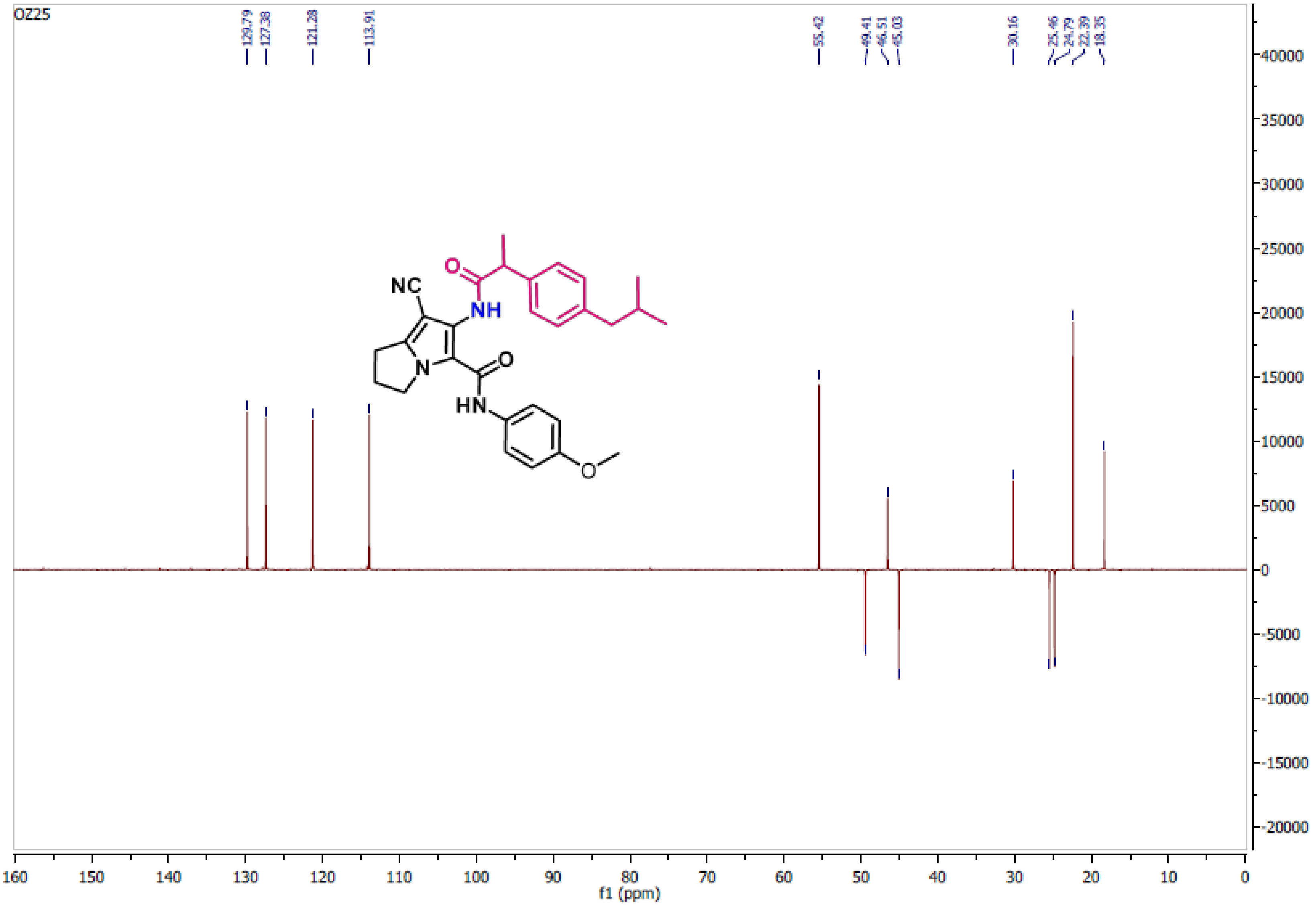

Fig. S18. DEPT C^{135} ($CDCl_3$, 125 MHz, δ ppm) of compound **8a** **(zoom on aliphatic Cs)**

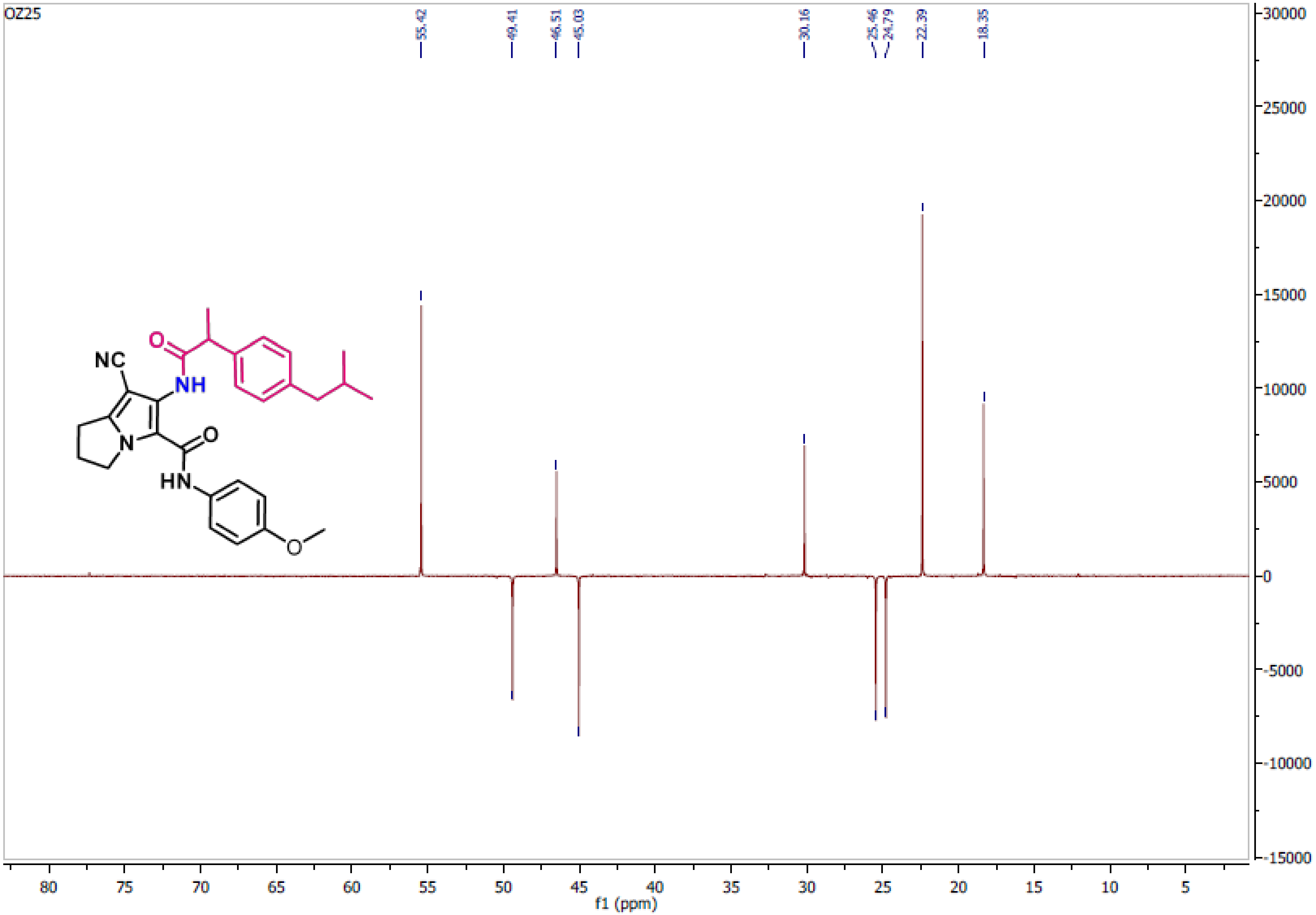

Fig. S19. DEPT C^{135} ($CDCl_3$, 125 MHz, δ ppm) of compound **8a** **(zoom on aromatic Cs)**

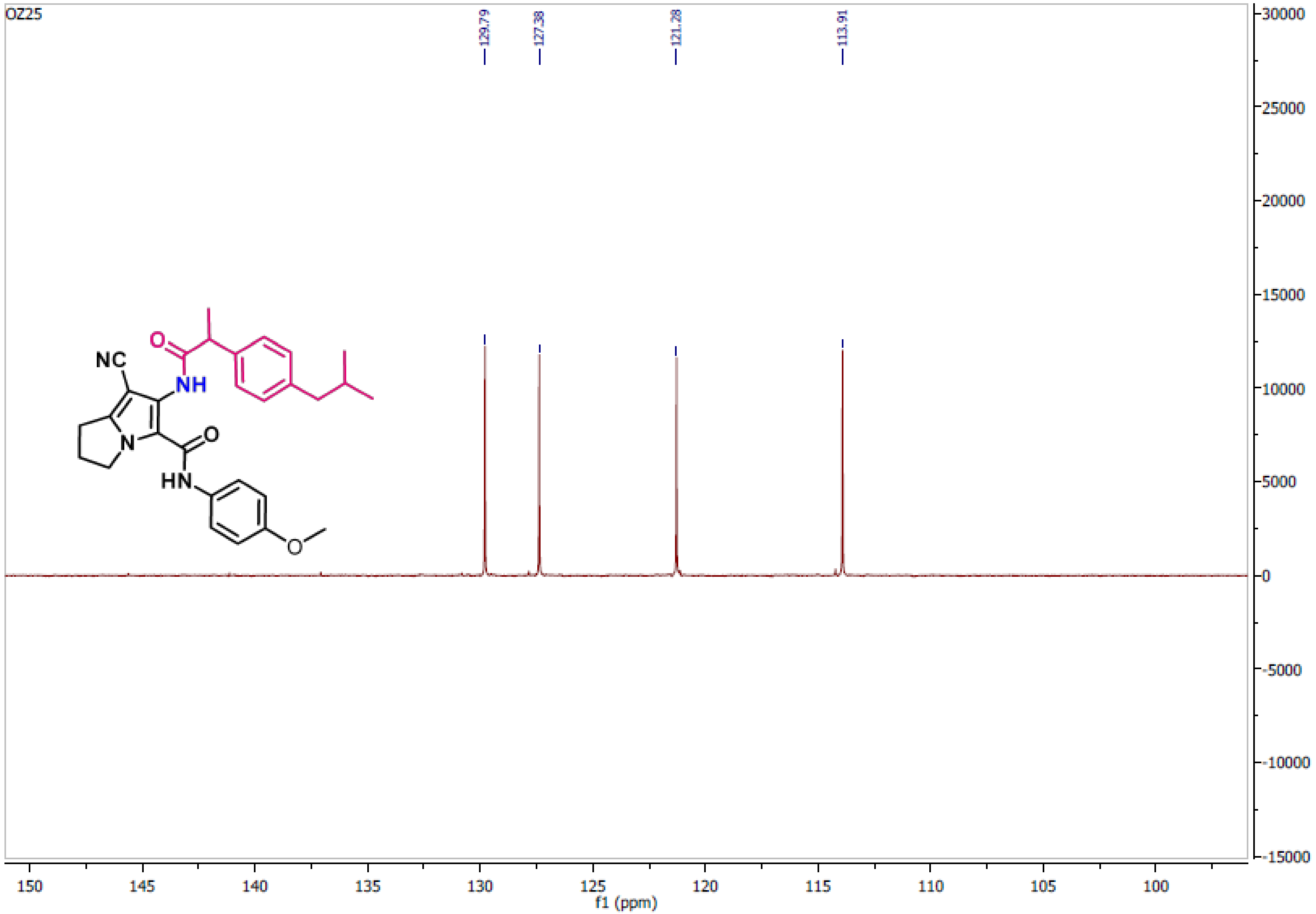

Fig. S20. ^{1}H-NMR ($CDCl_3$, 500 MHz, δ ppm) spectrum of compound **8b**

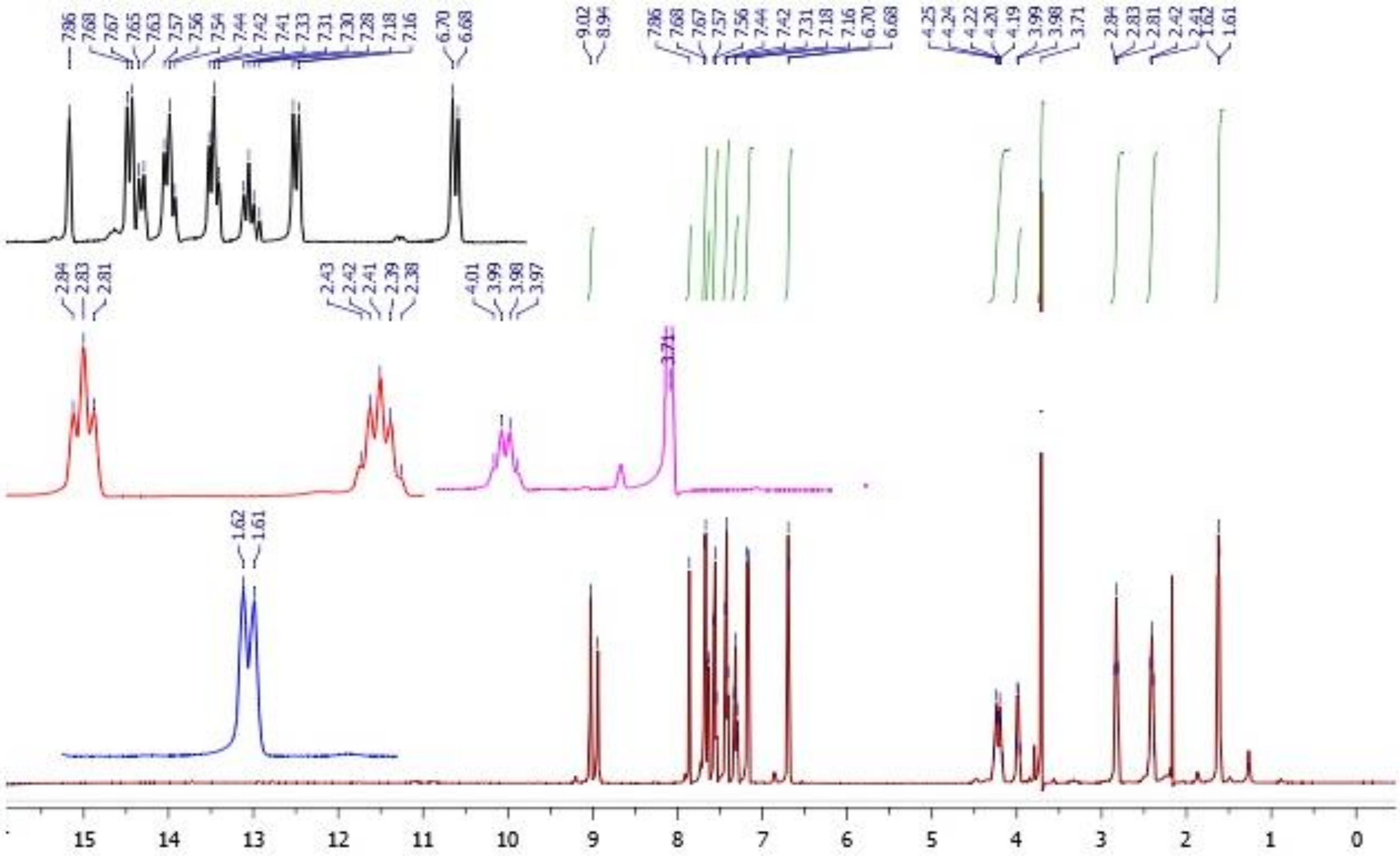

Fig. S21. ^{1}H-NMR ($CDCl_3$, 500 MHz, δ ppm) spectrum of compound **8b**

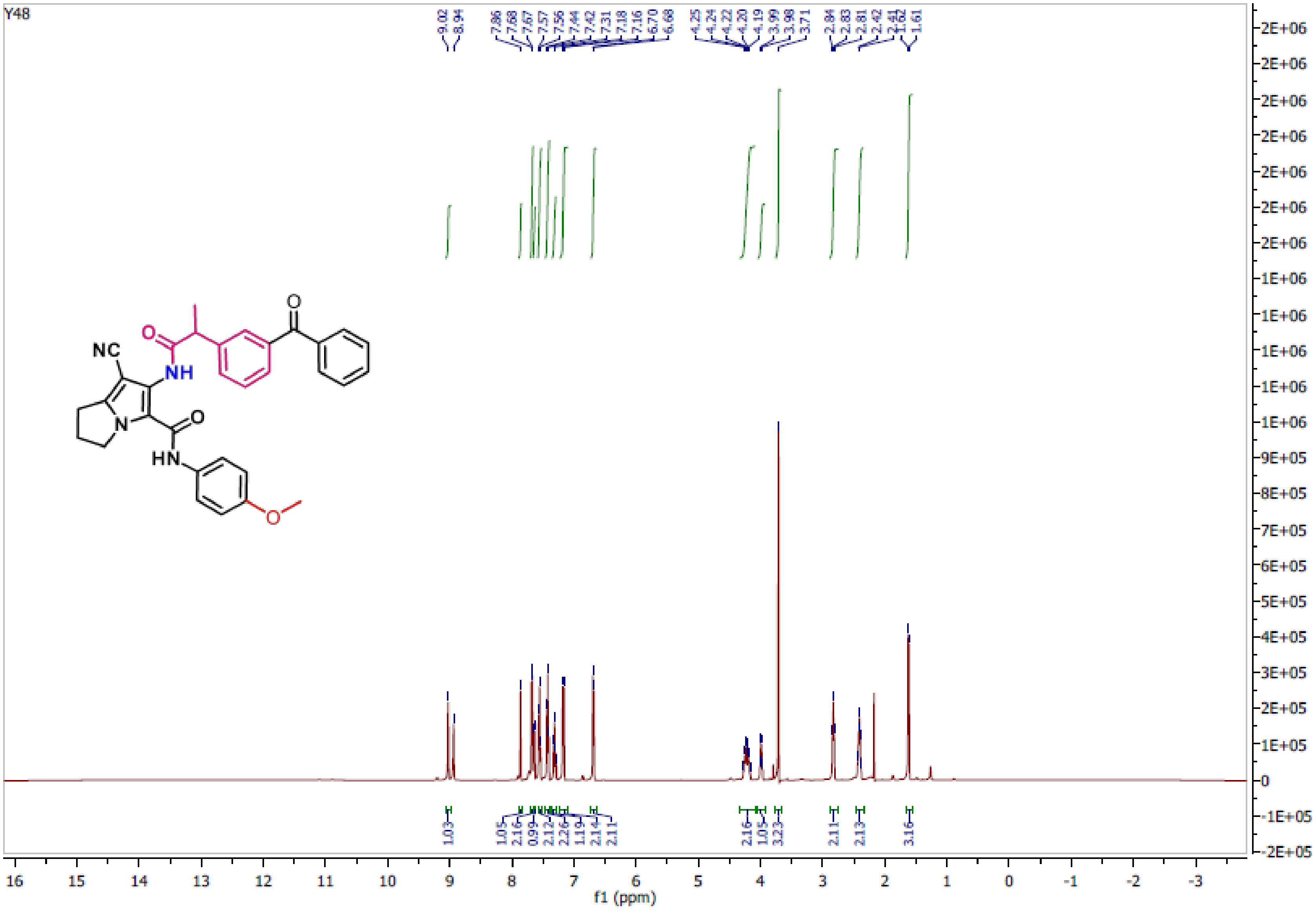

Fig. S22. ^{1}H-NMR ($CDCl_3$, 500 MHz, δ ppm) spectrum of compound **8b** **(zoom on aliphatic Hs)**

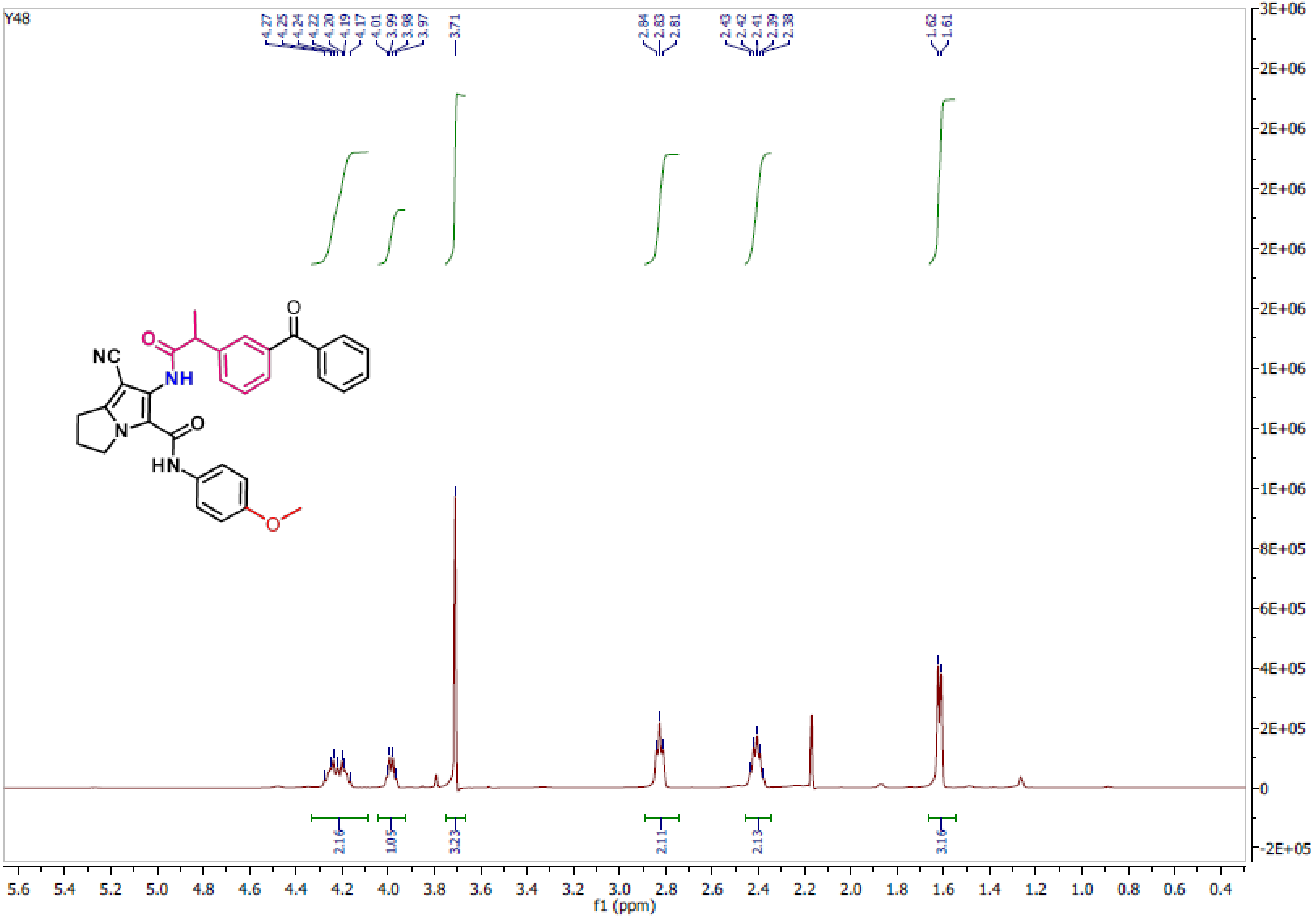

Fig. S23. ^{1}H-NMR ($CDCl_3$, 500 MHz, δ ppm) spectrum of compound **8b** **(zoom on aromatic Hs)**

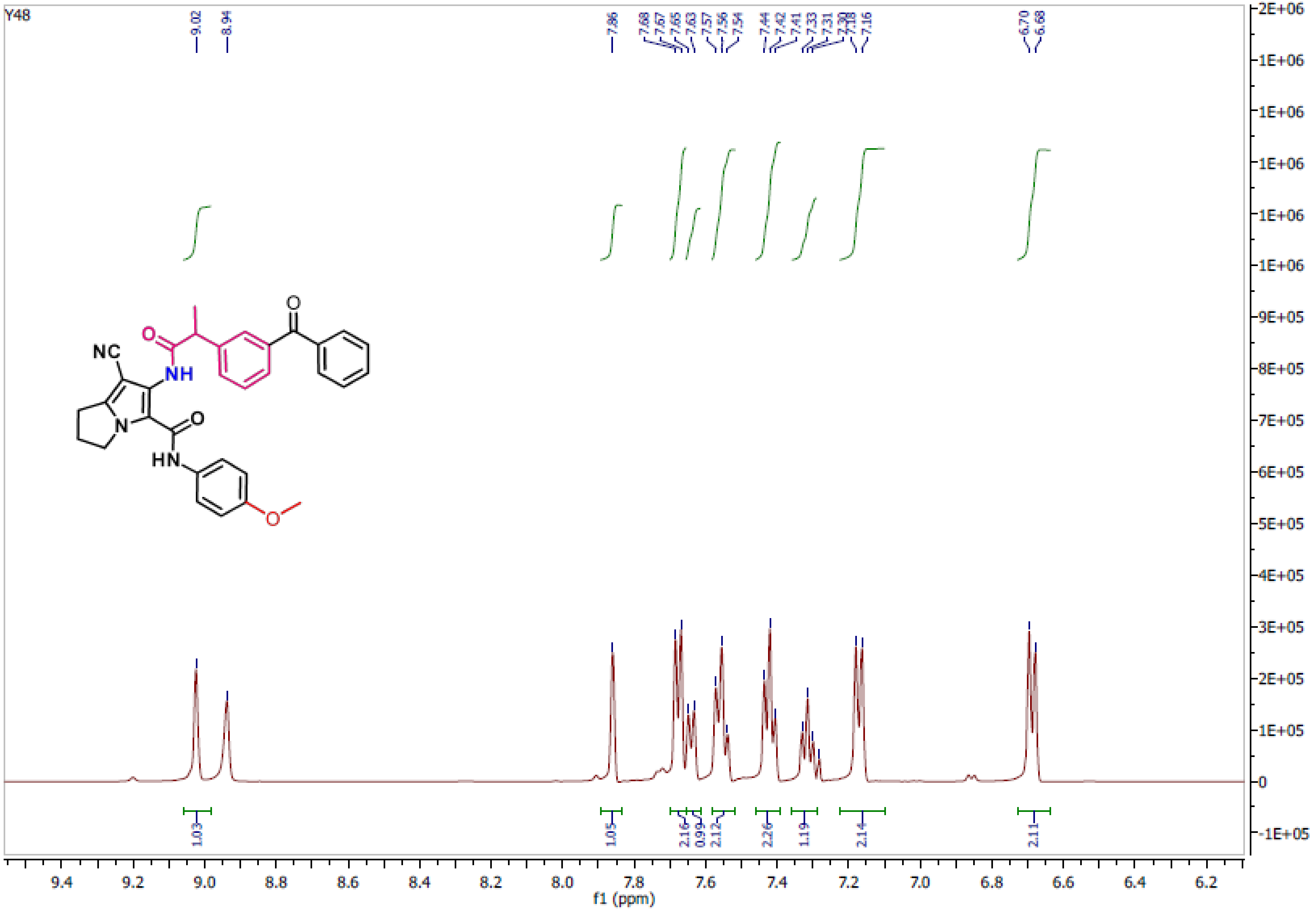

Fig. S24. ^{13}C-NMR ($CDCl_3$, 125 MHz, δ ppm) spectrum of compound **8b**

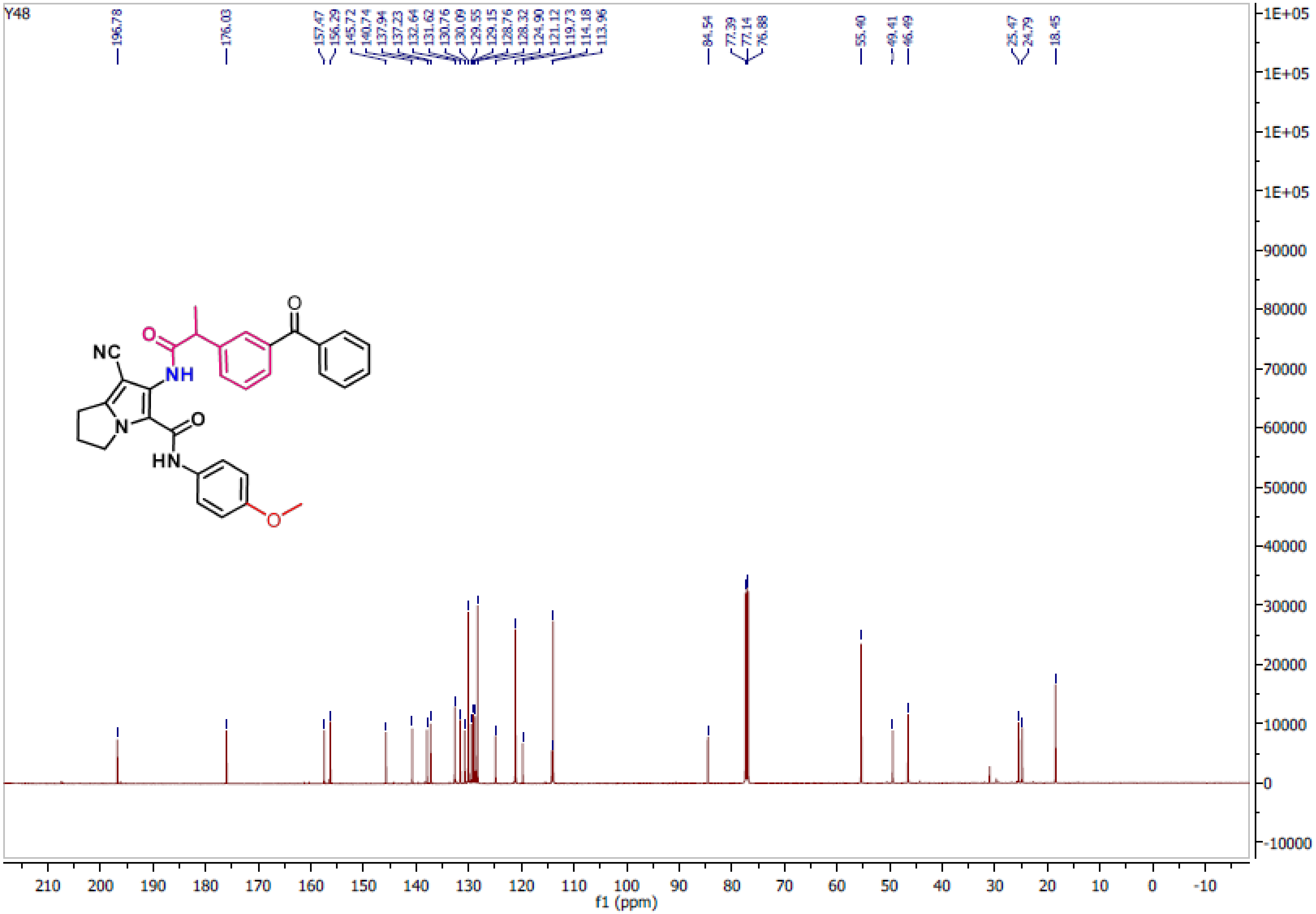

Fig. S25. ^{13}C-NMR ($CDCl_3$, 125 MHz, δ ppm) spectrum of compound **8b** **(zoom on aliphatic Cs)**

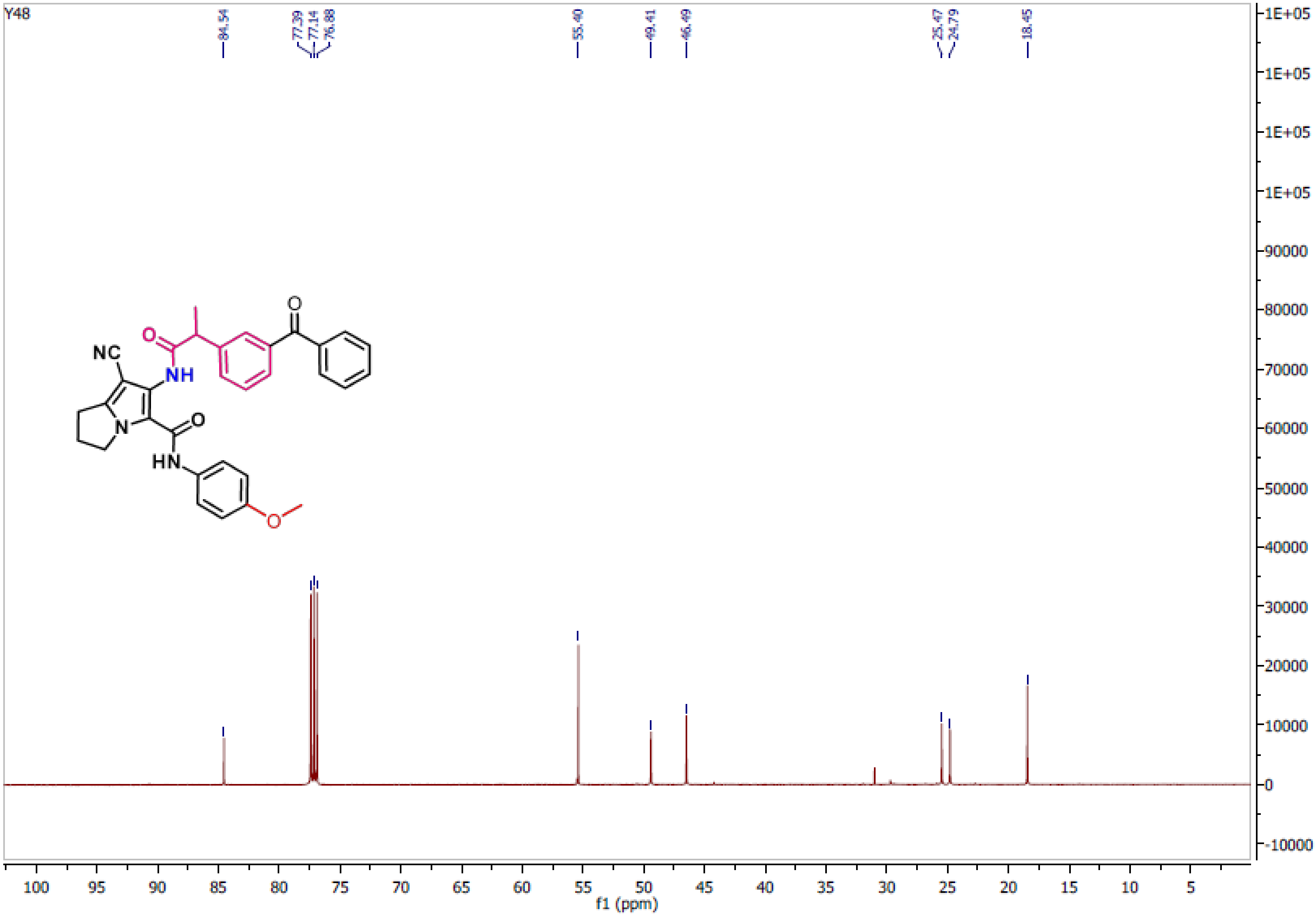

Supplementary materials

Fig. S26. ^{13}C-NMR ($CDCl_3$, 125 MHz, δ ppm) spectrum of compound **8b** **(zoom on aromatic Cs)**

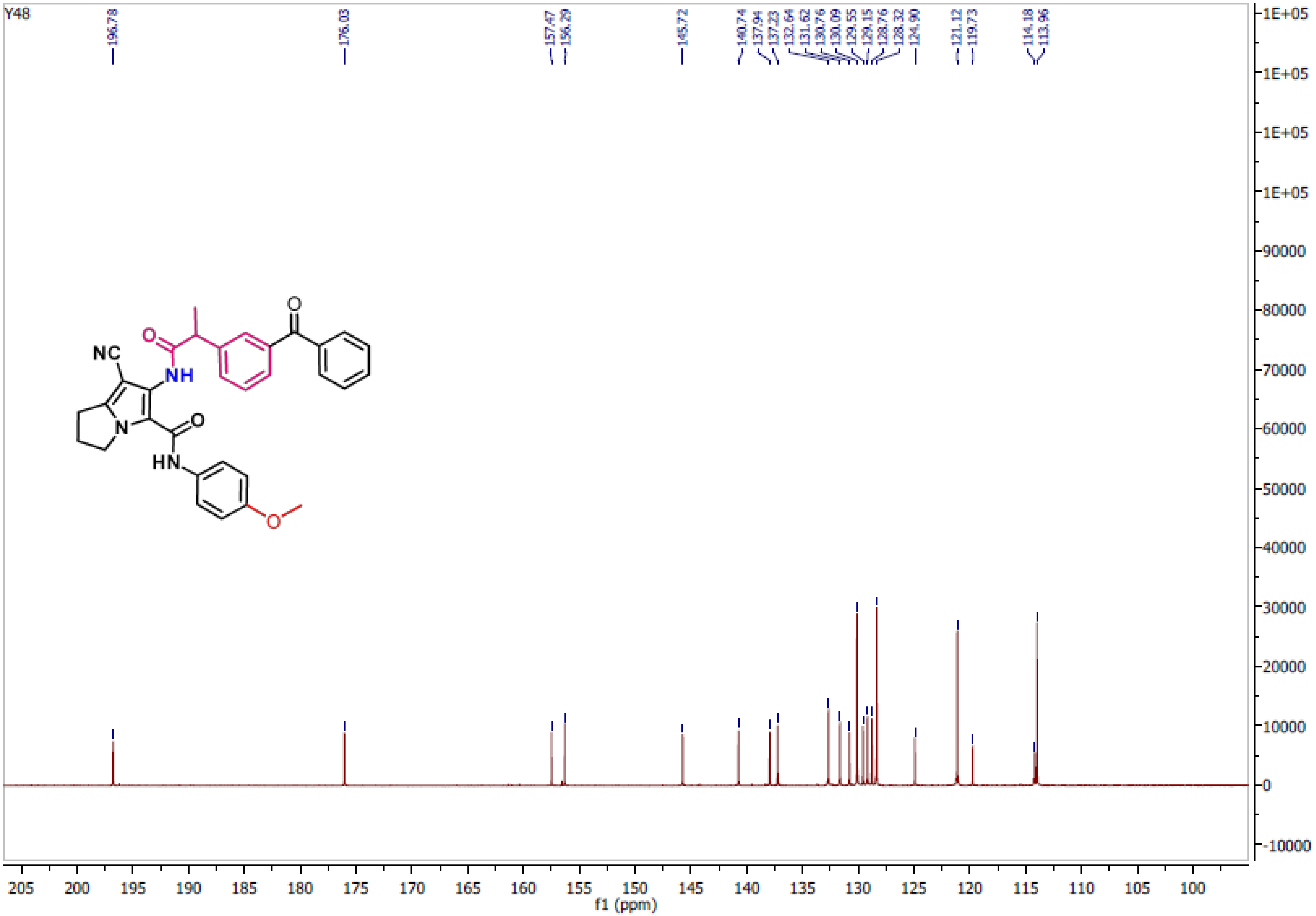

Fig. S27. DEPT C^{135} (CDCl$_3$, 125 MHz, δ ppm) of compound **8b**

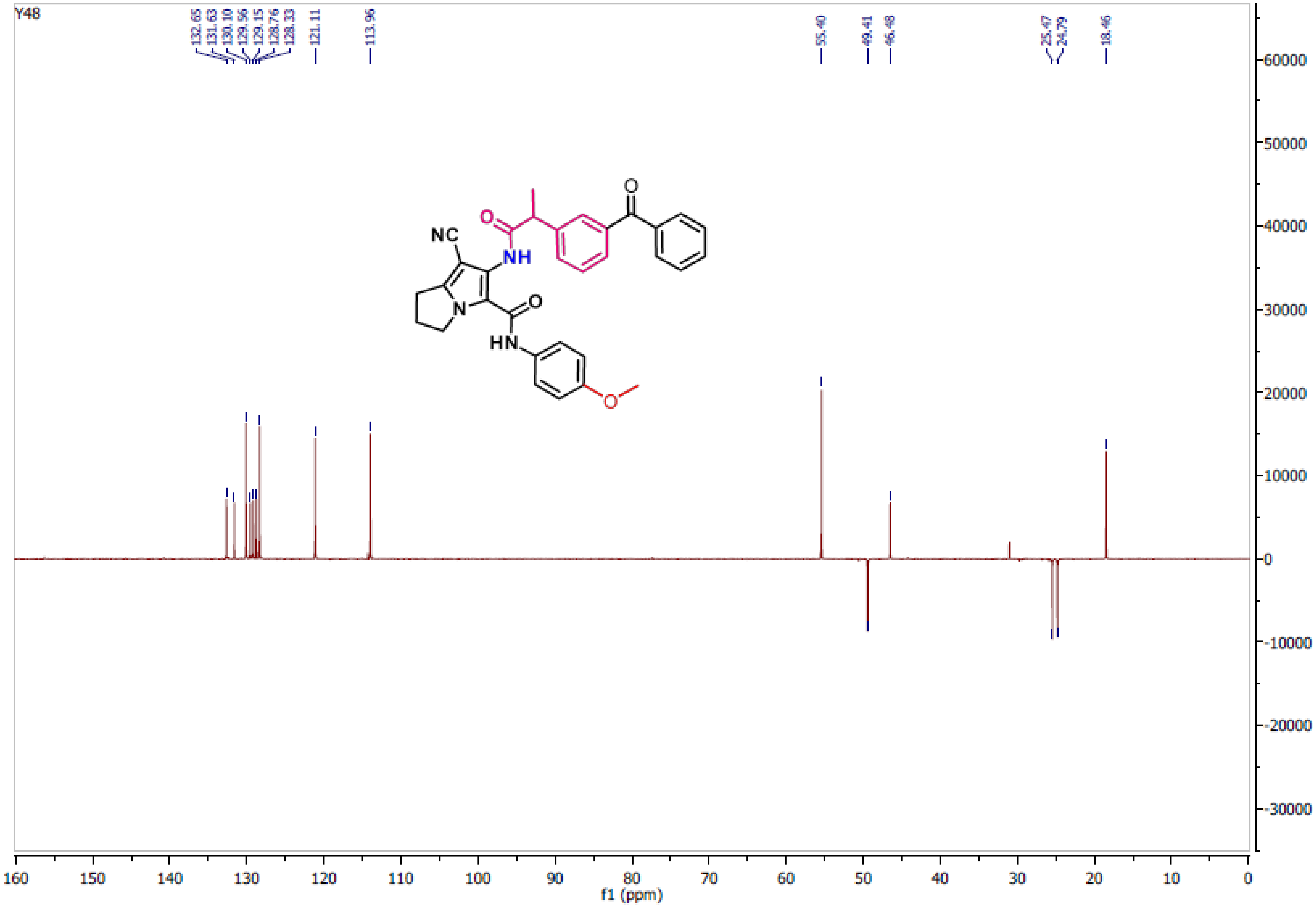

Fig. S28. DEPT C^{135} (CDCl$_3$, 125 MHz, δ ppm) of compound **8b** **(zoom on aliphatic Cs)**

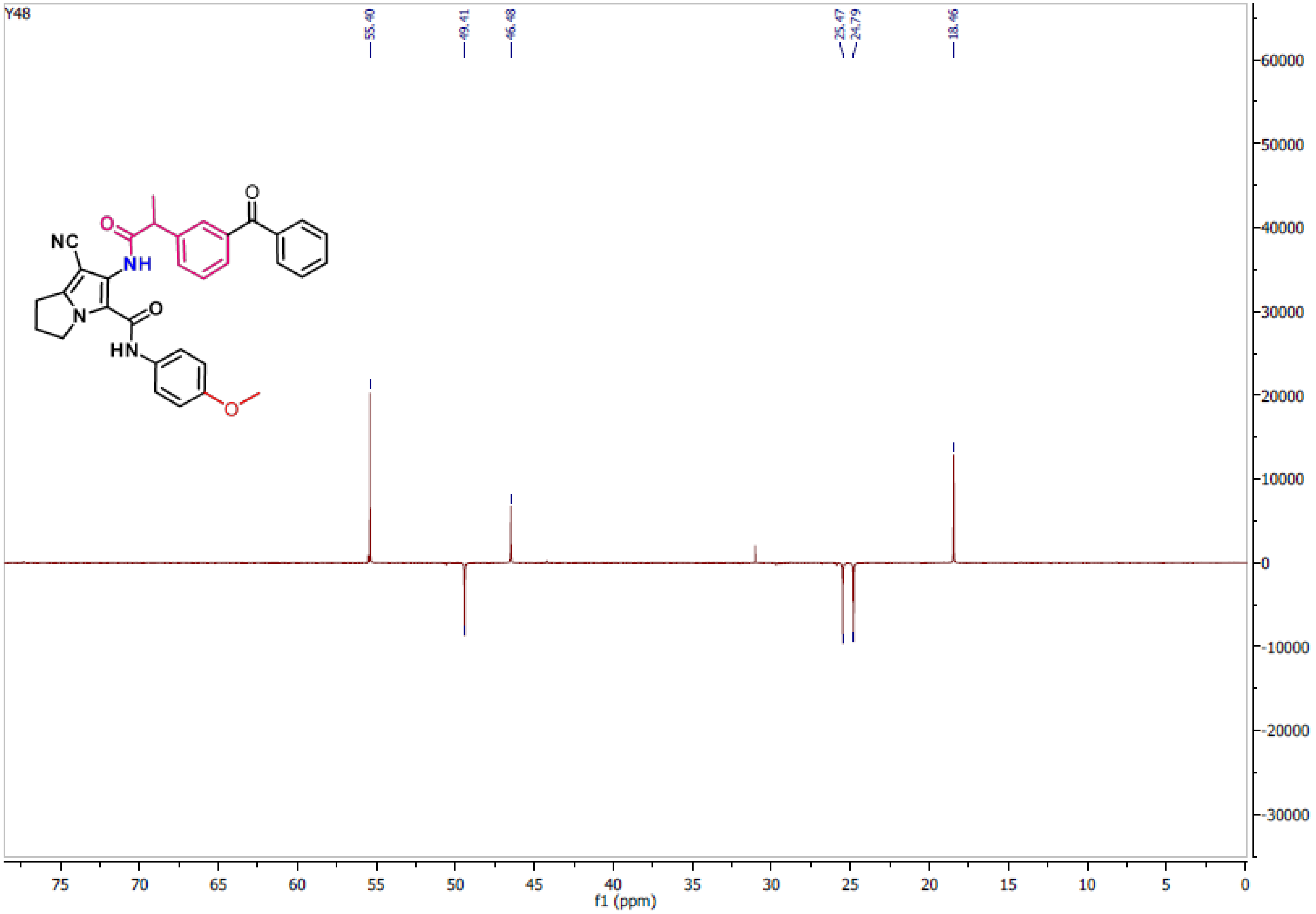

Fig. S29. DEPT C^{135} ($CDCl_3$, 125 MHz, δ ppm) of compound **8b** **(zoom on aromatic Cs)**

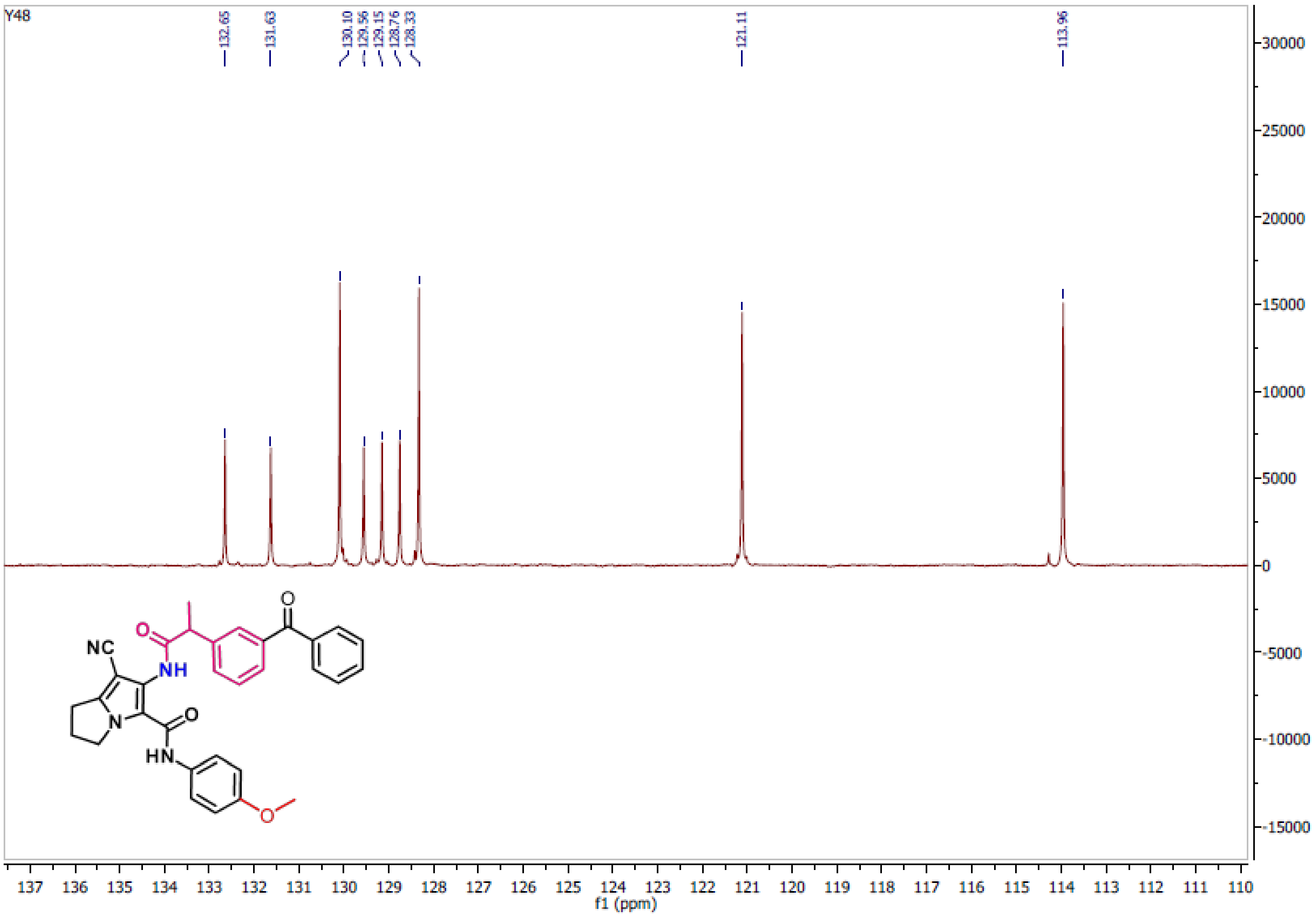

Fig. S30. ^{1}H-NMR (DMSO-d_6, 500 MHz, δ ppm) spectrum of compound **8c**

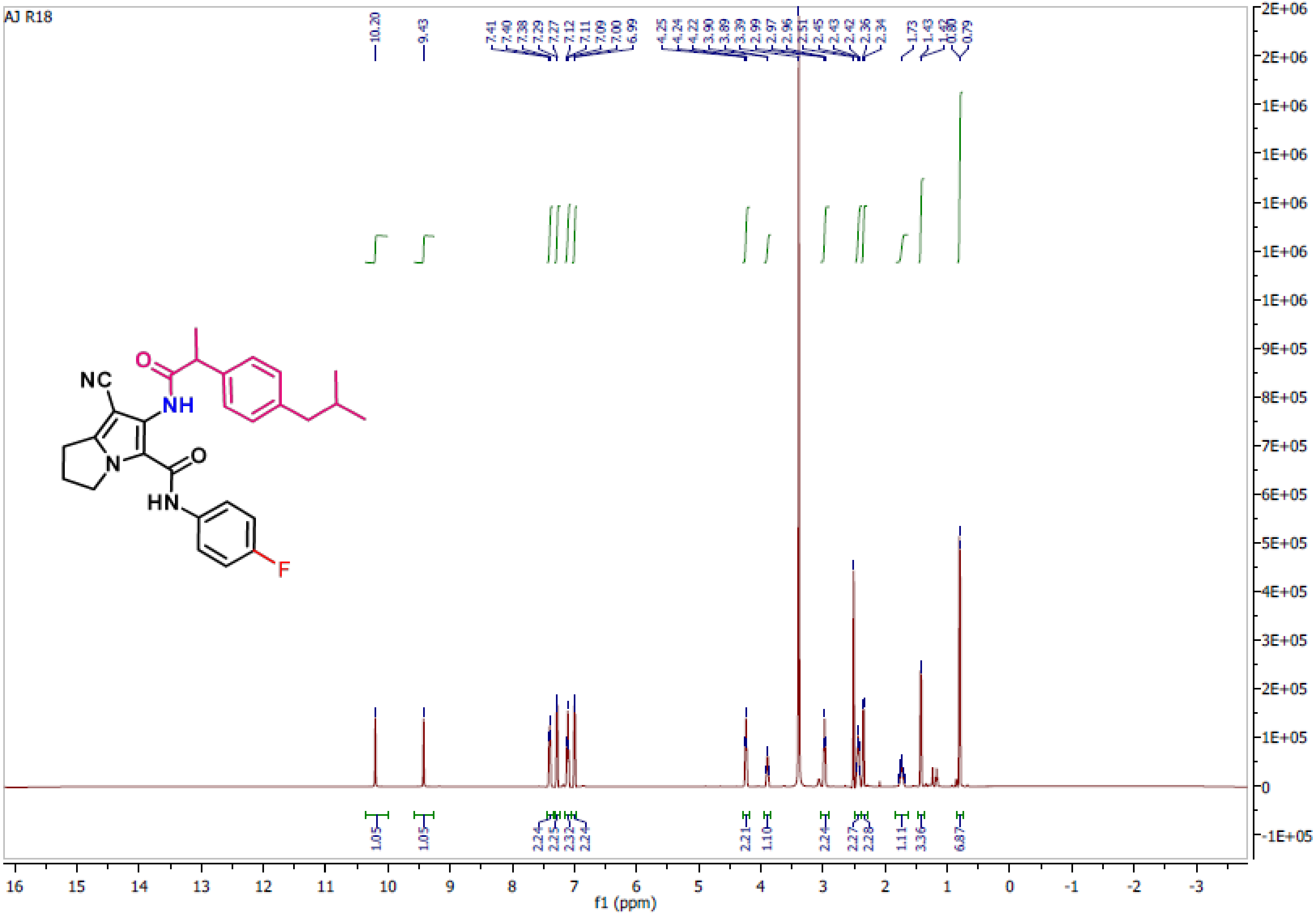

Fig. S31. ^{1}H-NMR (DMSO-d_6, 500 MHz, δ ppm) spectrum of compound **8c** **(zoon on aliphatic Hs)**

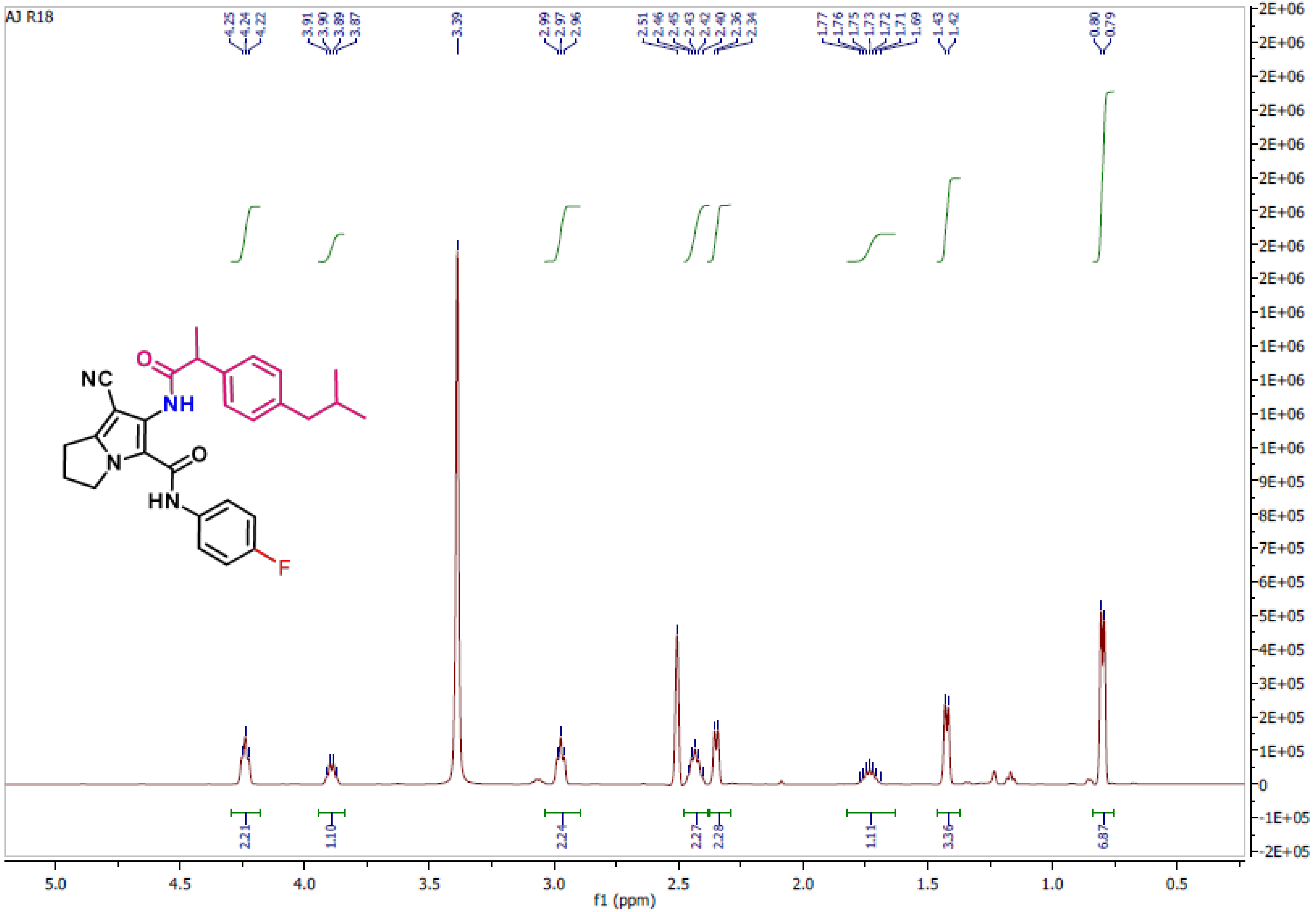

Fig. S32. ^{1}H-NMR (DMSO-d_6, 500 MHz, δ ppm) spectrum of compound **8c** **(zoon on aromatic Hs)**

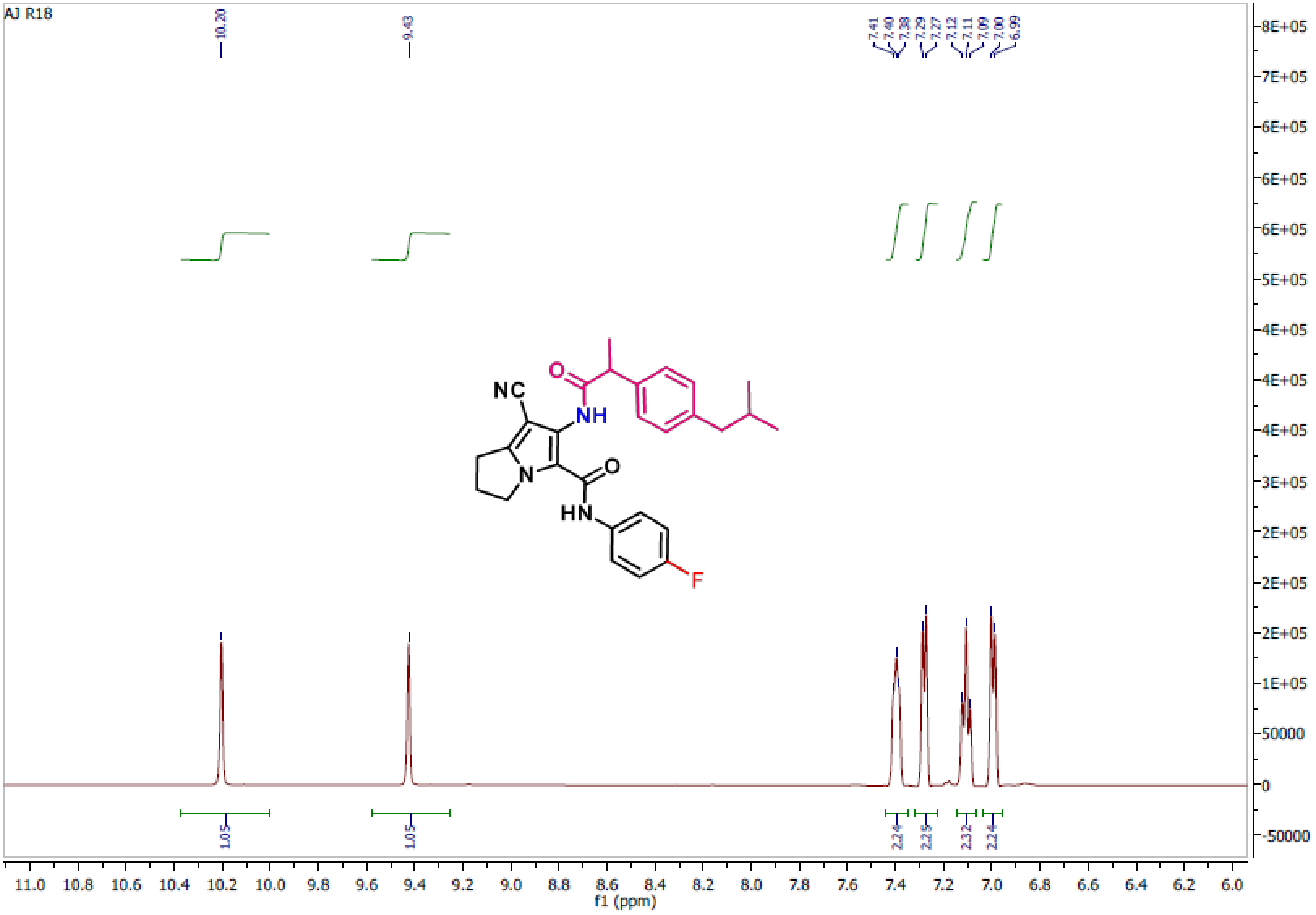

Fig. S33. ^{13}C-NMR (DMSO, 125 MHz, *δ* ppm) spectrum of compound **8c**

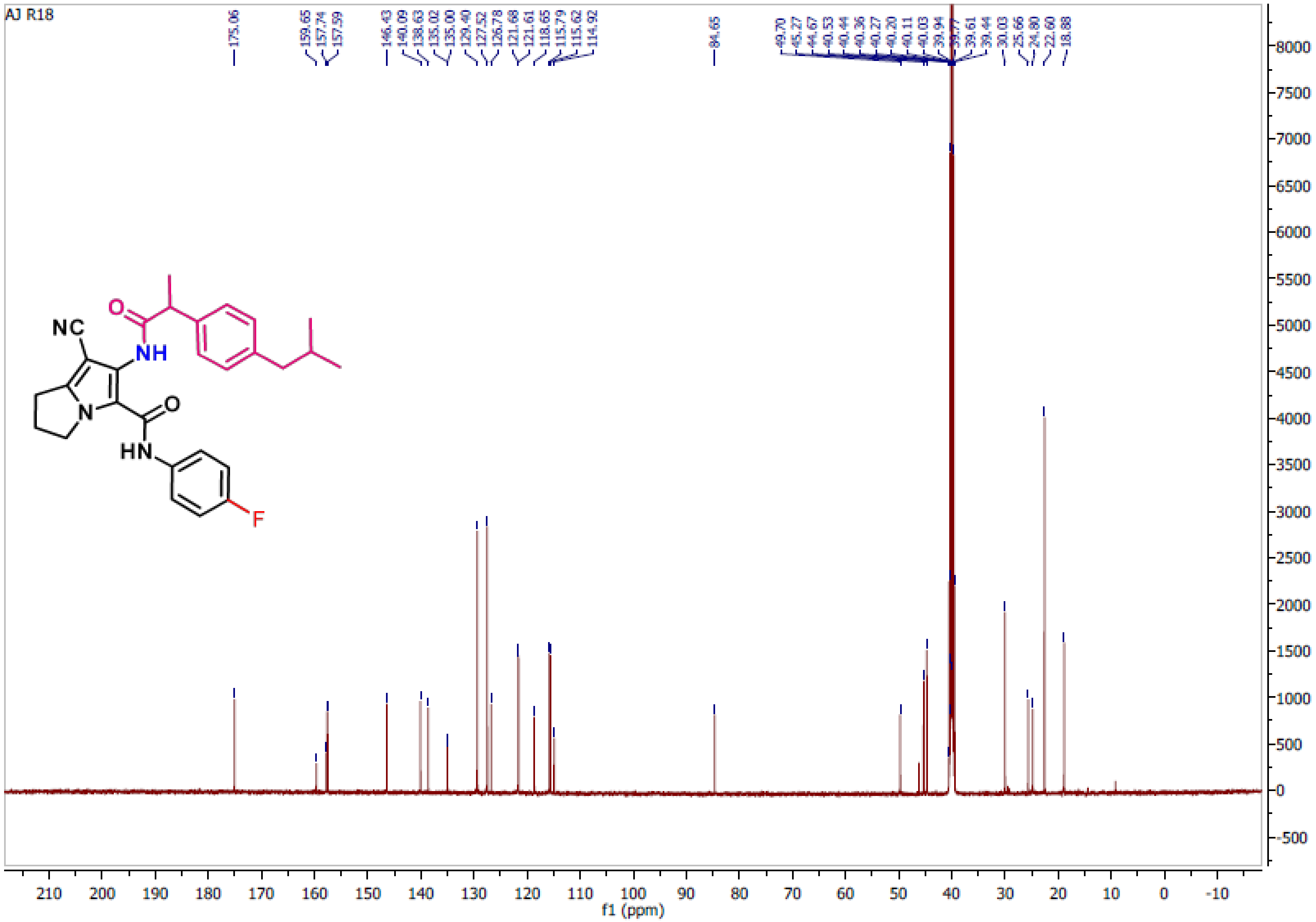

Fig. S34. ^{13}C-NMR (DMSO, 125 MHz, δ ppm) spectrum of compound **8c** **(zoon on aliphatic Cs)**

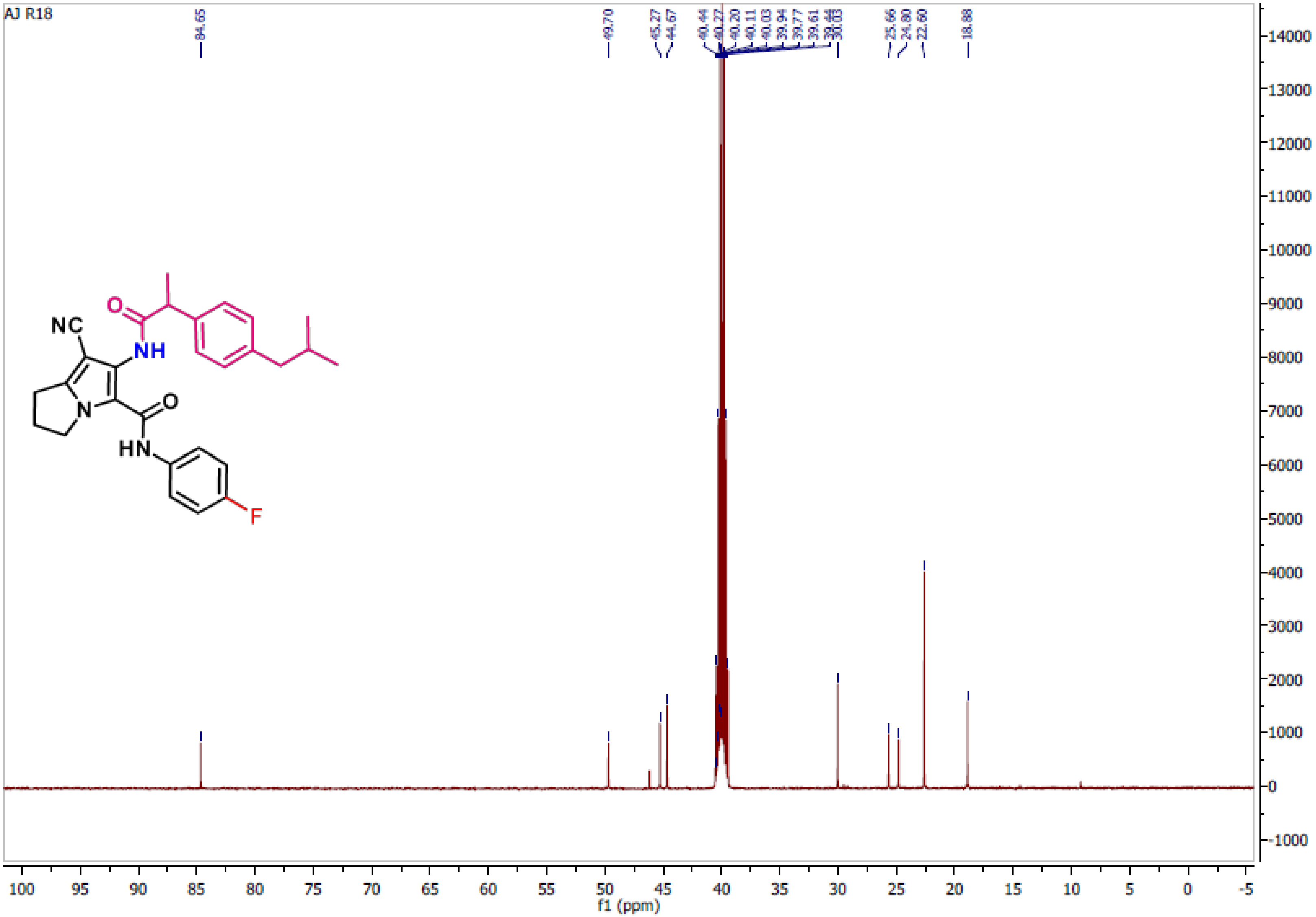

Fig. S35. ^{13}C-NMR (DMSO, 125 MHz, δ ppm) spectrum of compound **8c** **(zoon on aromatic Cs)**

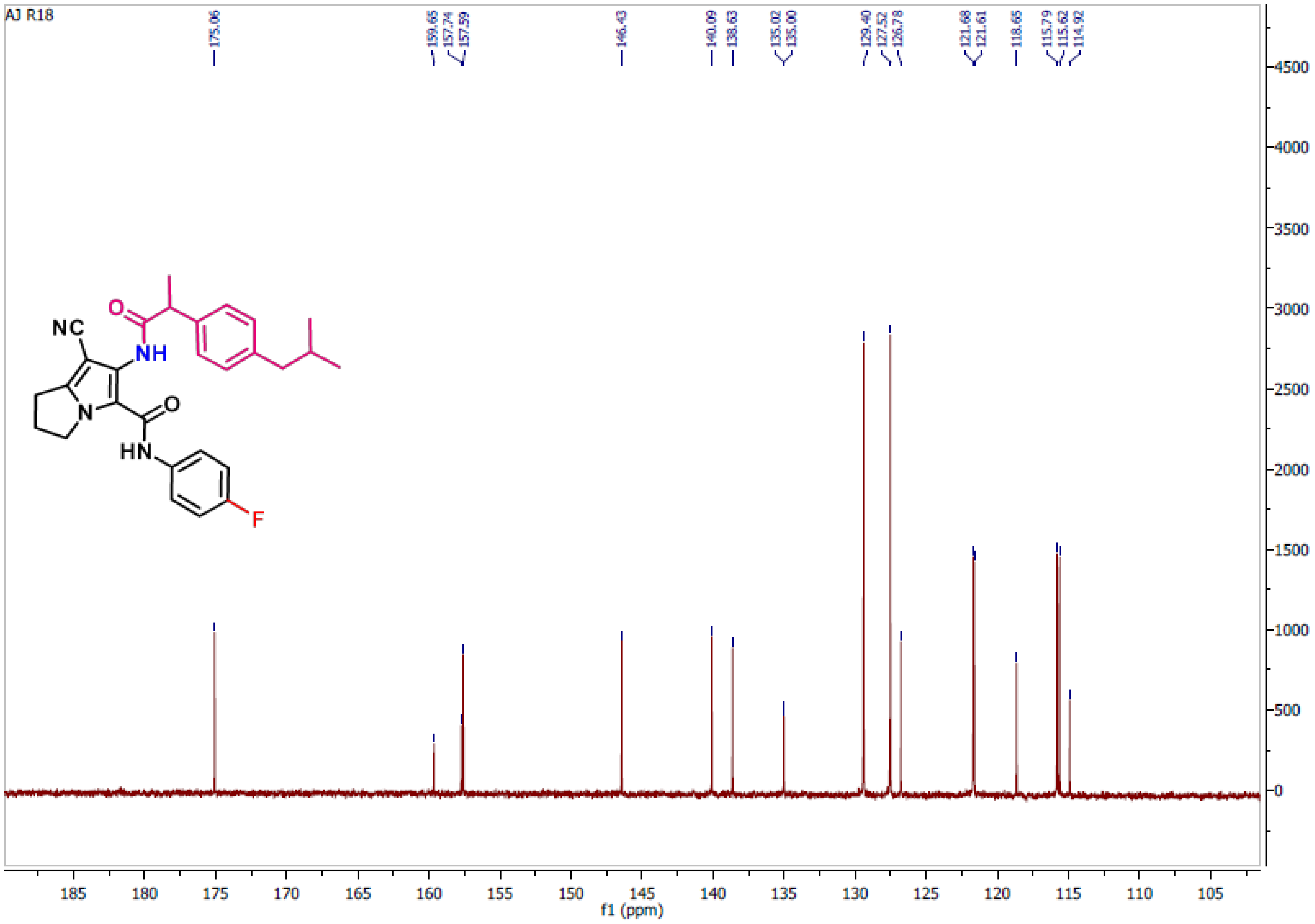

Fig. S36. ^{13}C-NMR (DMSO, 125 MHz, δ ppm) spectrum of compound **8c** **(zoon on aromatic Cs)**

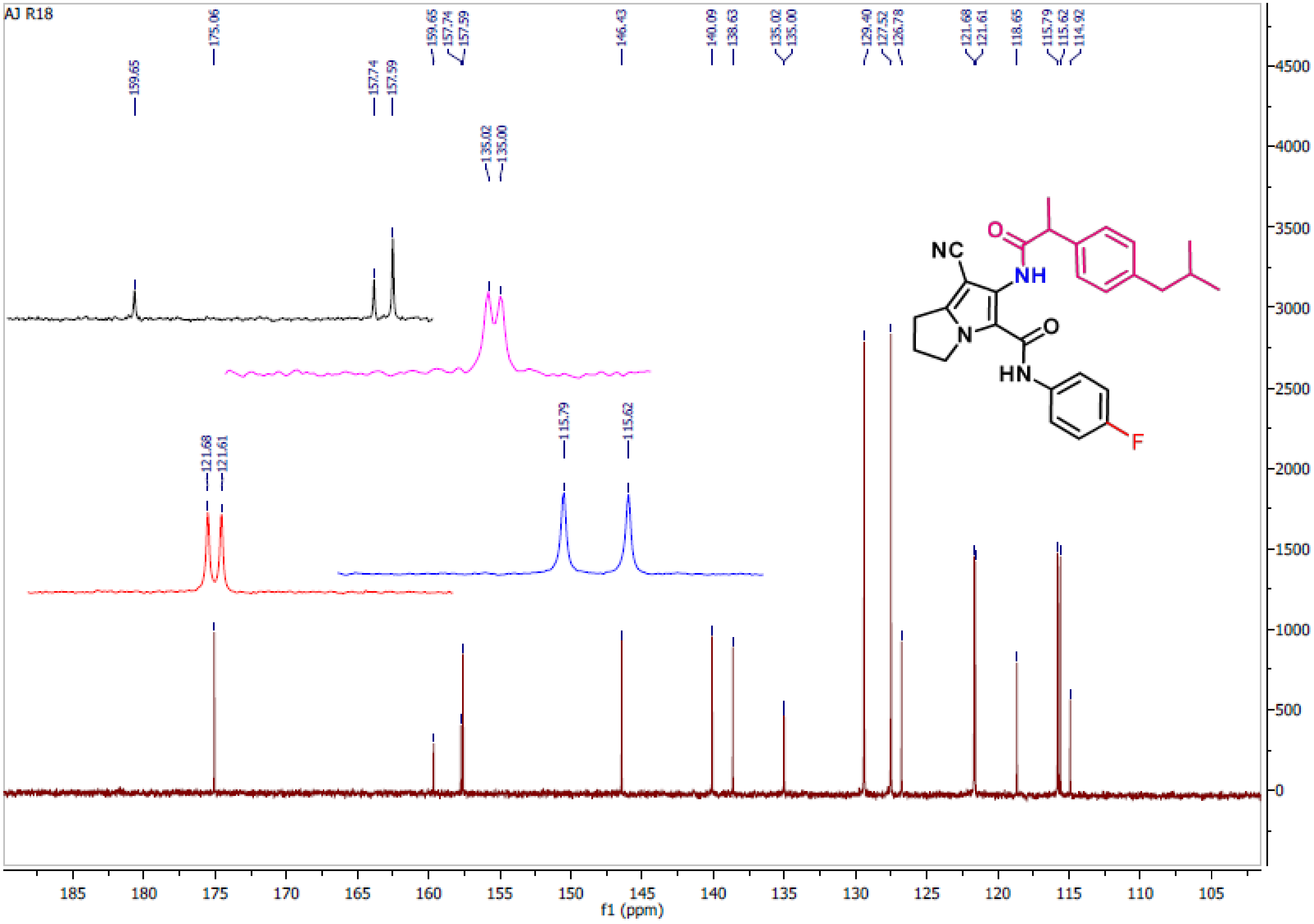

Fig. S37. ^{1}H-NMR (DMSO-d_6, 500 MHz, δ ppm) spectrum of compound **8d**

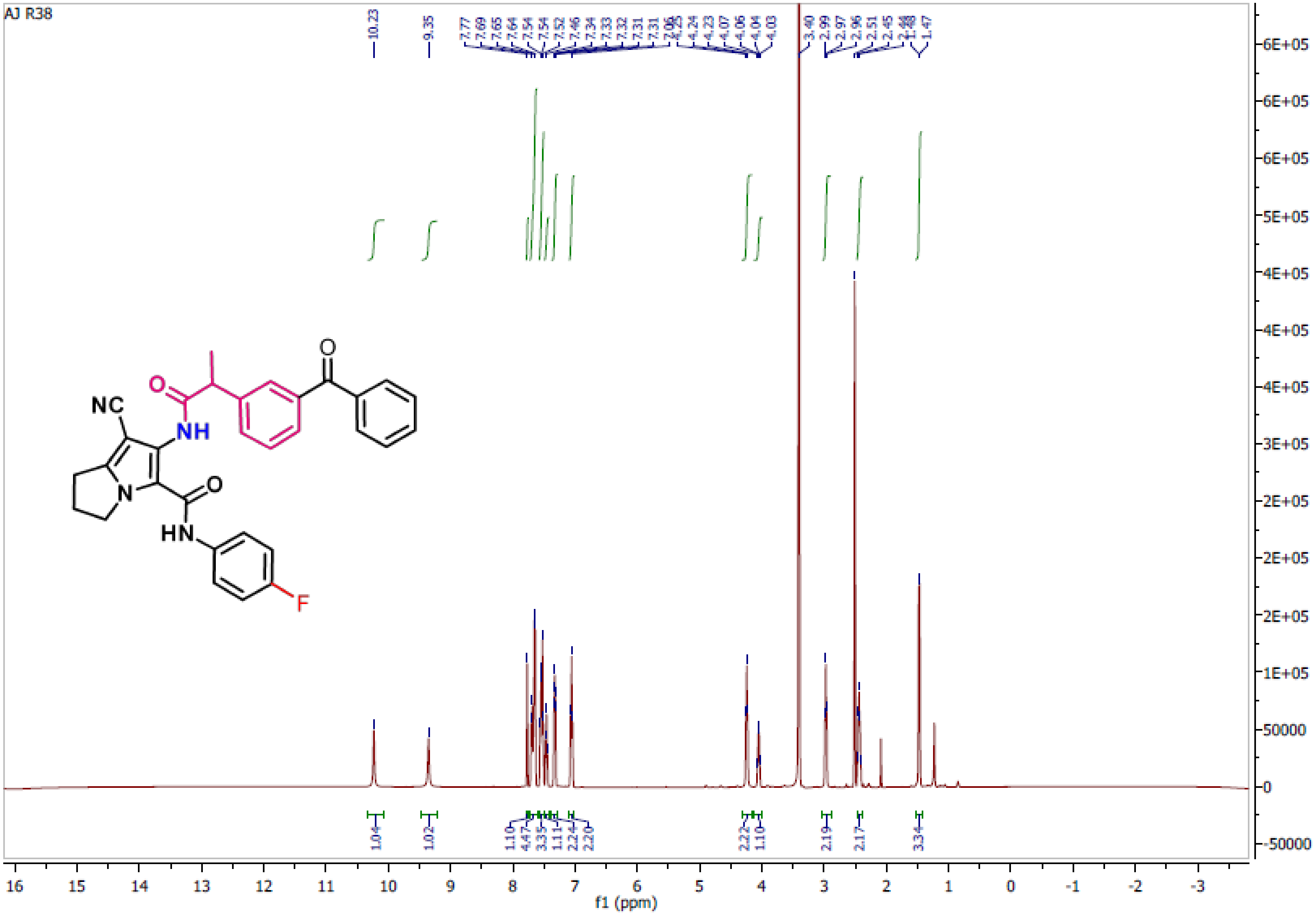

Fig. S38. ^{1}H-NMR (DMSO-d_6, 500 MHz, δ ppm) spectrum of compound **8d** **(zoom on aliphatic Hs)**

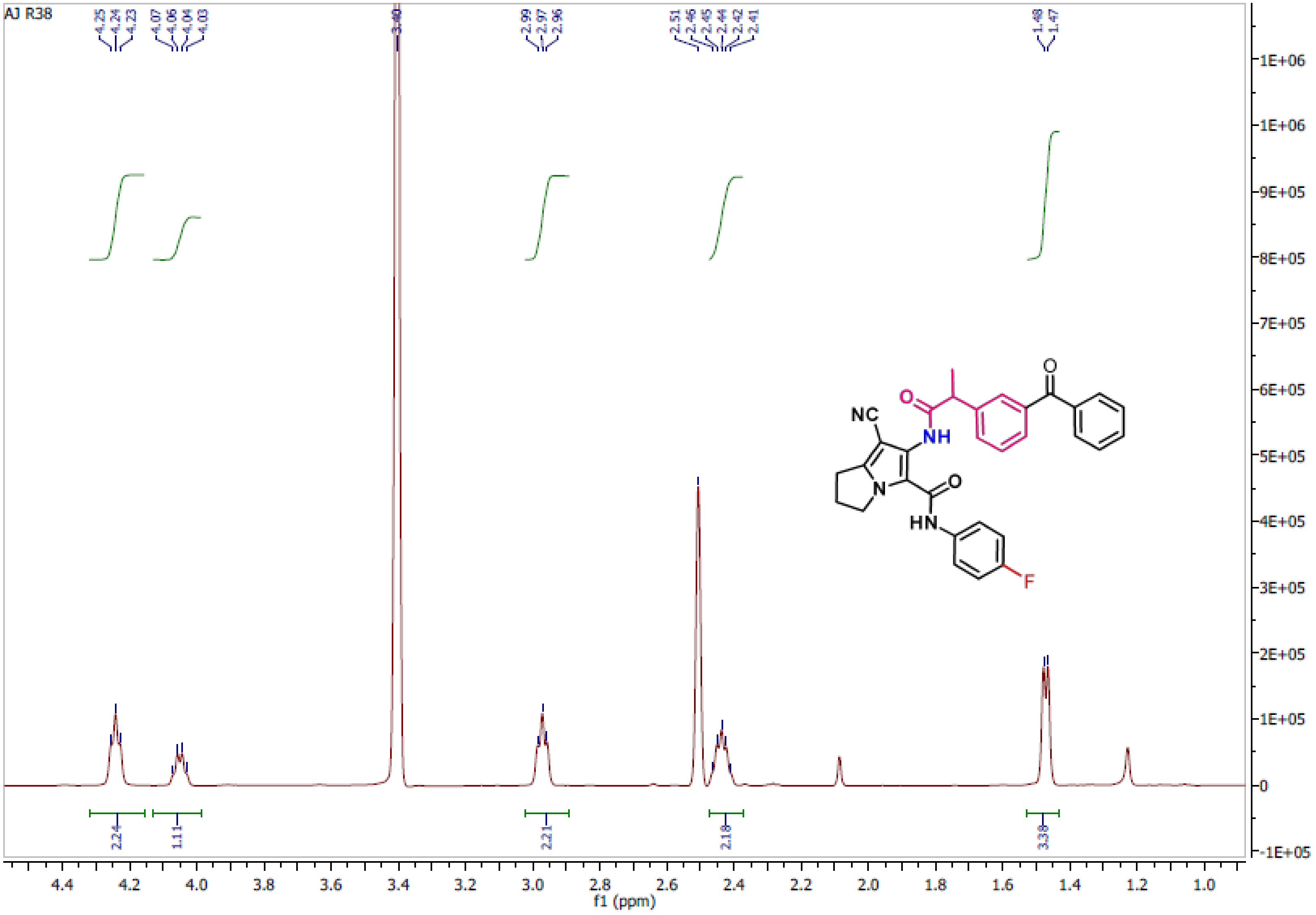

Fig. S39. ^{1}H-NMR (DMSO-d_6, 500 MHz, δ ppm) spectrum of compound **8d** **(zoom on aromatic Hs)**

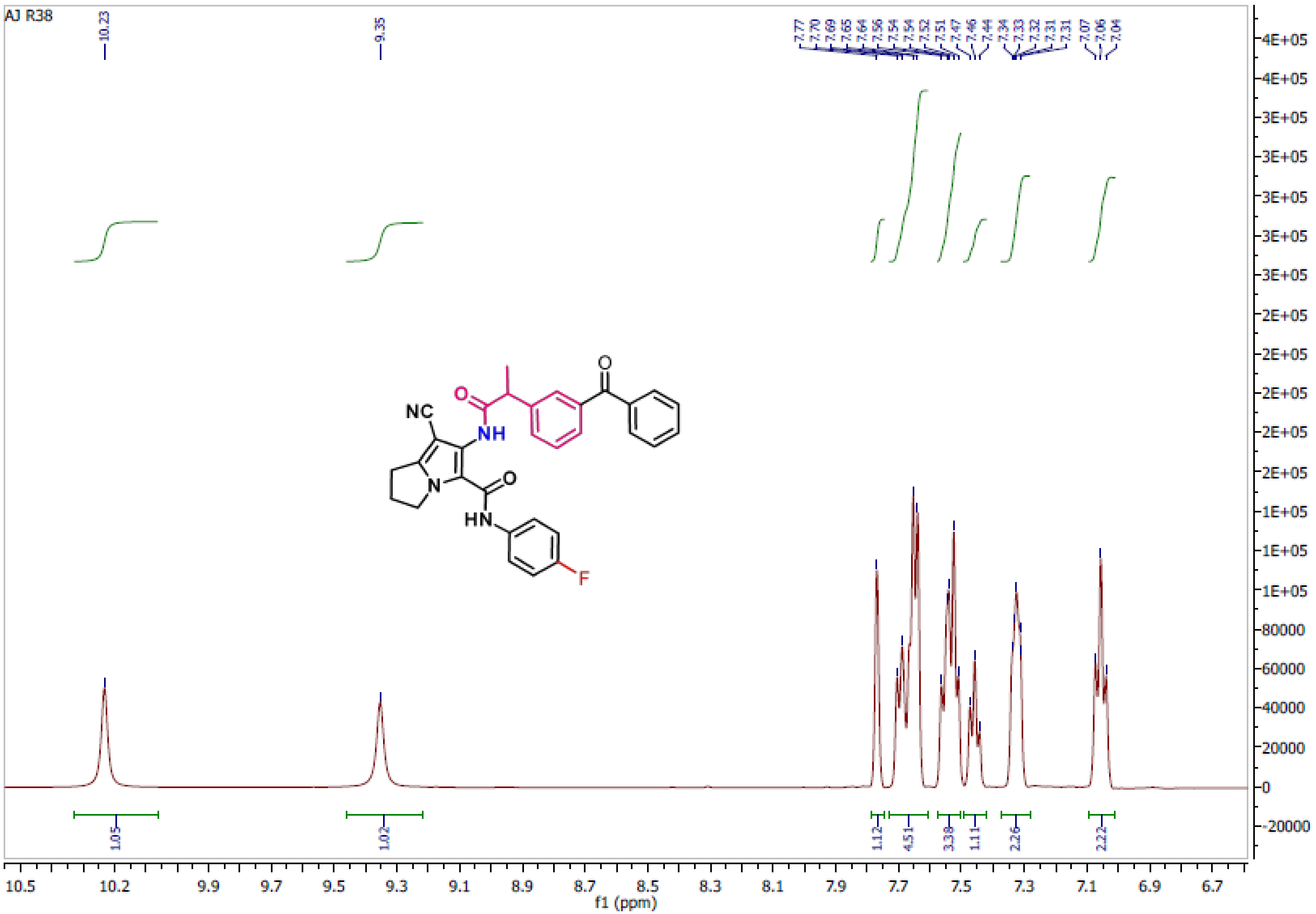

Fig. S40. ^{13}C-NMR (DMSO, 125 MHz, δ ppm) spectrum of compound **8d**

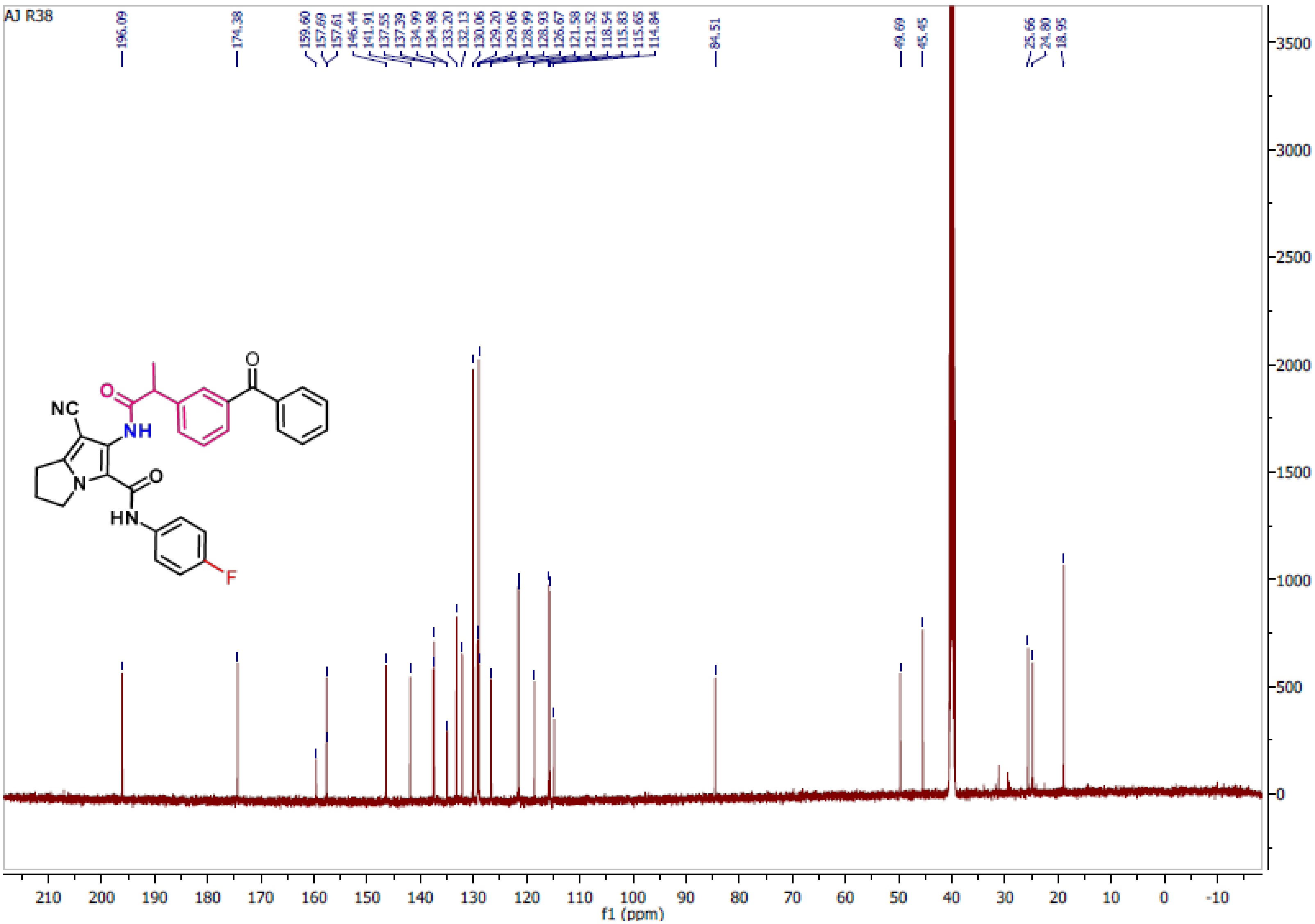

Fig. S41. ^{13}C-NMR (DMSO, 125 MHz, δ ppm) spectrum of compound **8d** **(zoom on aliphatic Cs)**

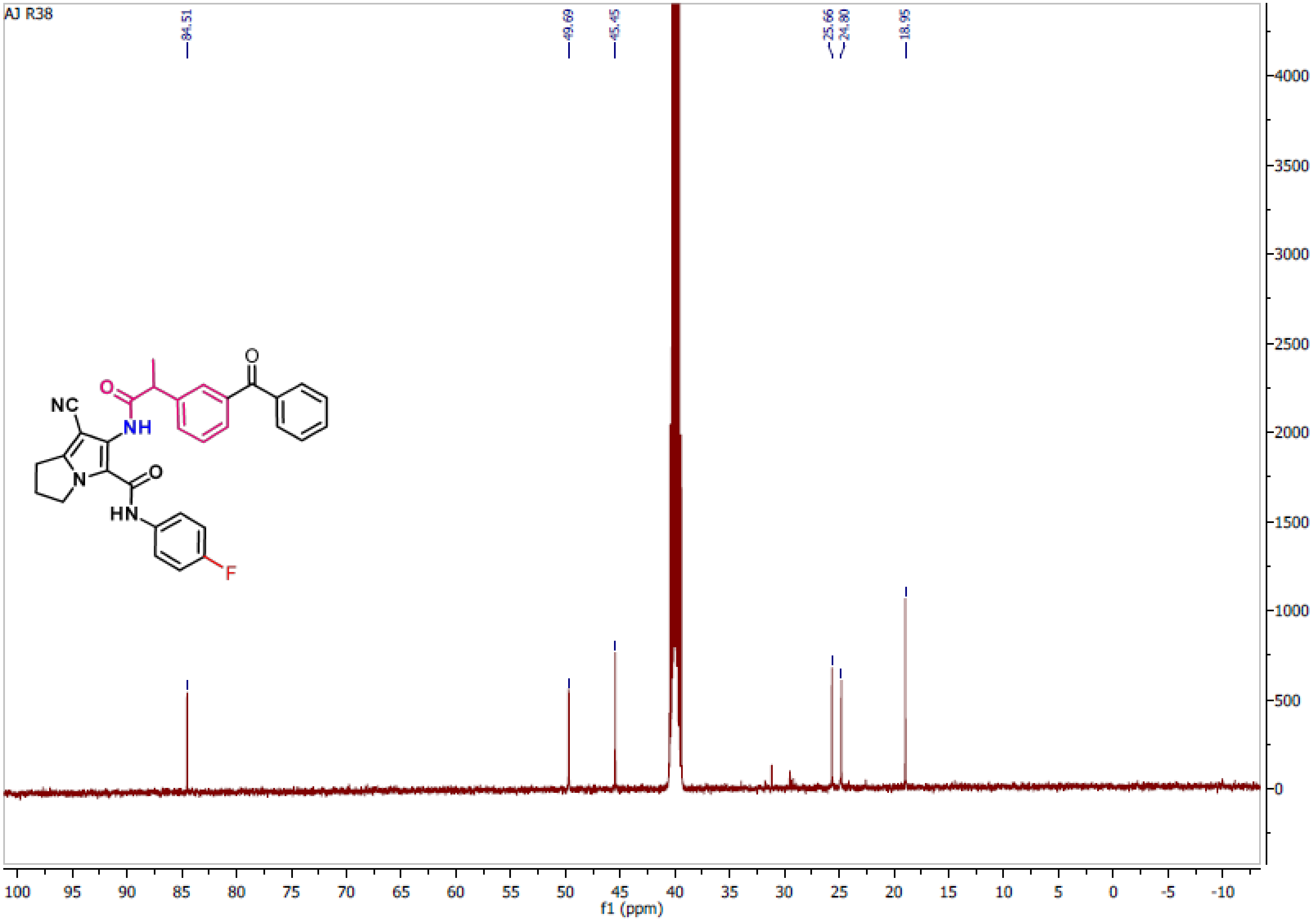

Fig. S42. ^{13}C-NMR (DMSO, 125 MHz, δ ppm) spectrum of compound **8d** **(zoom on aromatic Cs)**

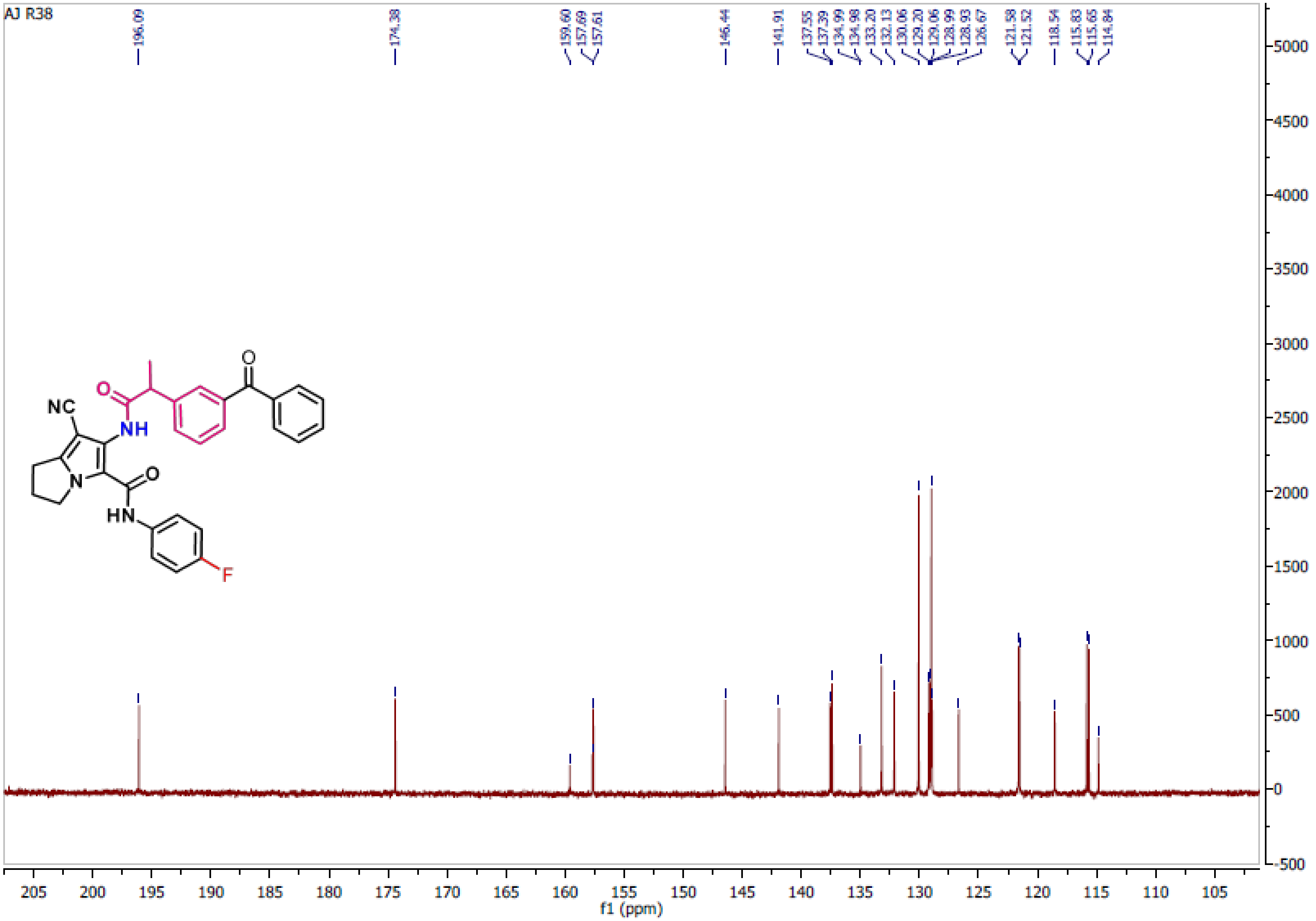

Fig. S43. ^{13}C-NMR (DMSO, 125 MHz, δ ppm) spectrum of compound **8d** **(zoom on aromatic Cs)**

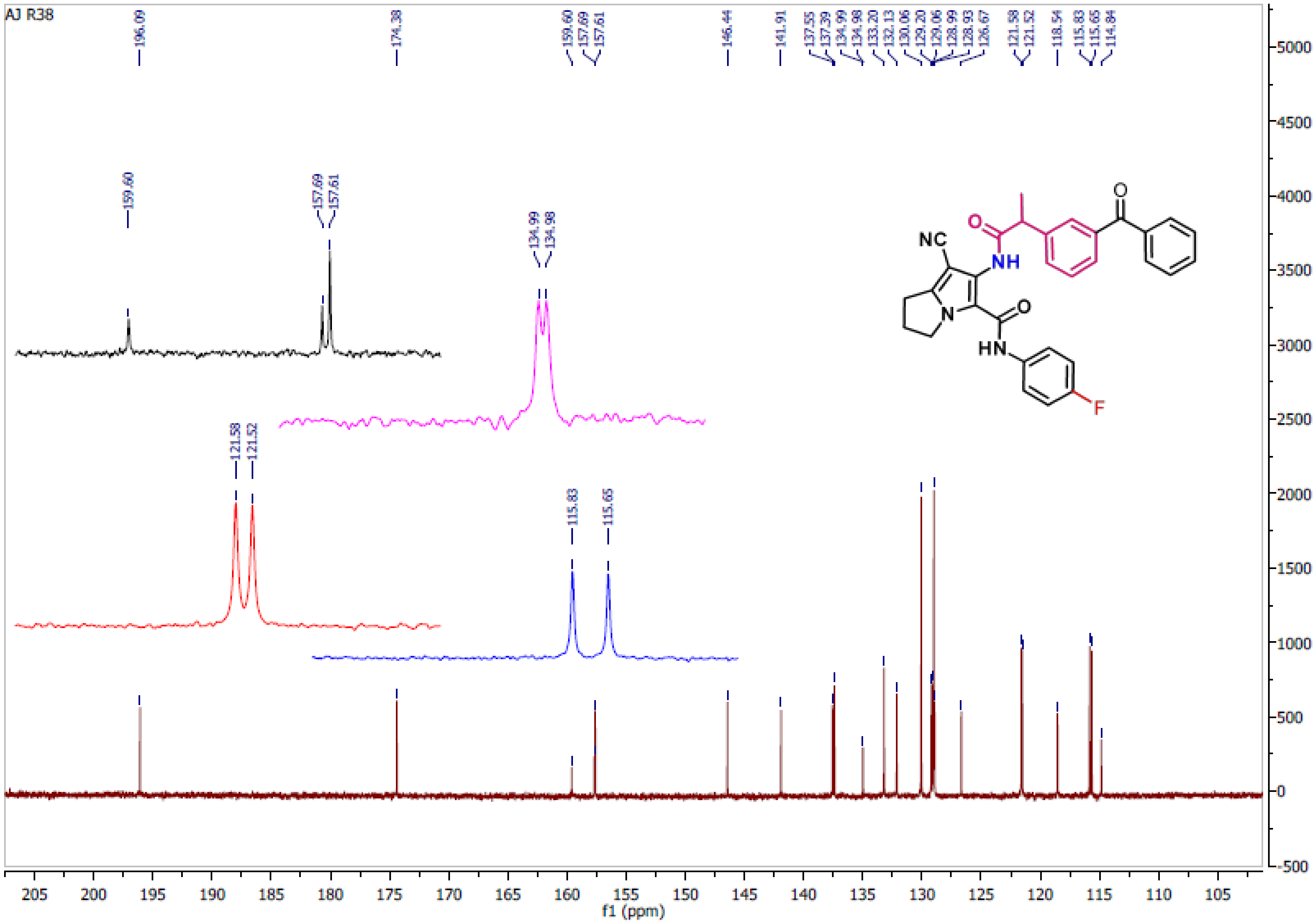

Fig. S44. DEPT C135 (DMSO, 125 MHz, δ ppm) of compound **8d**

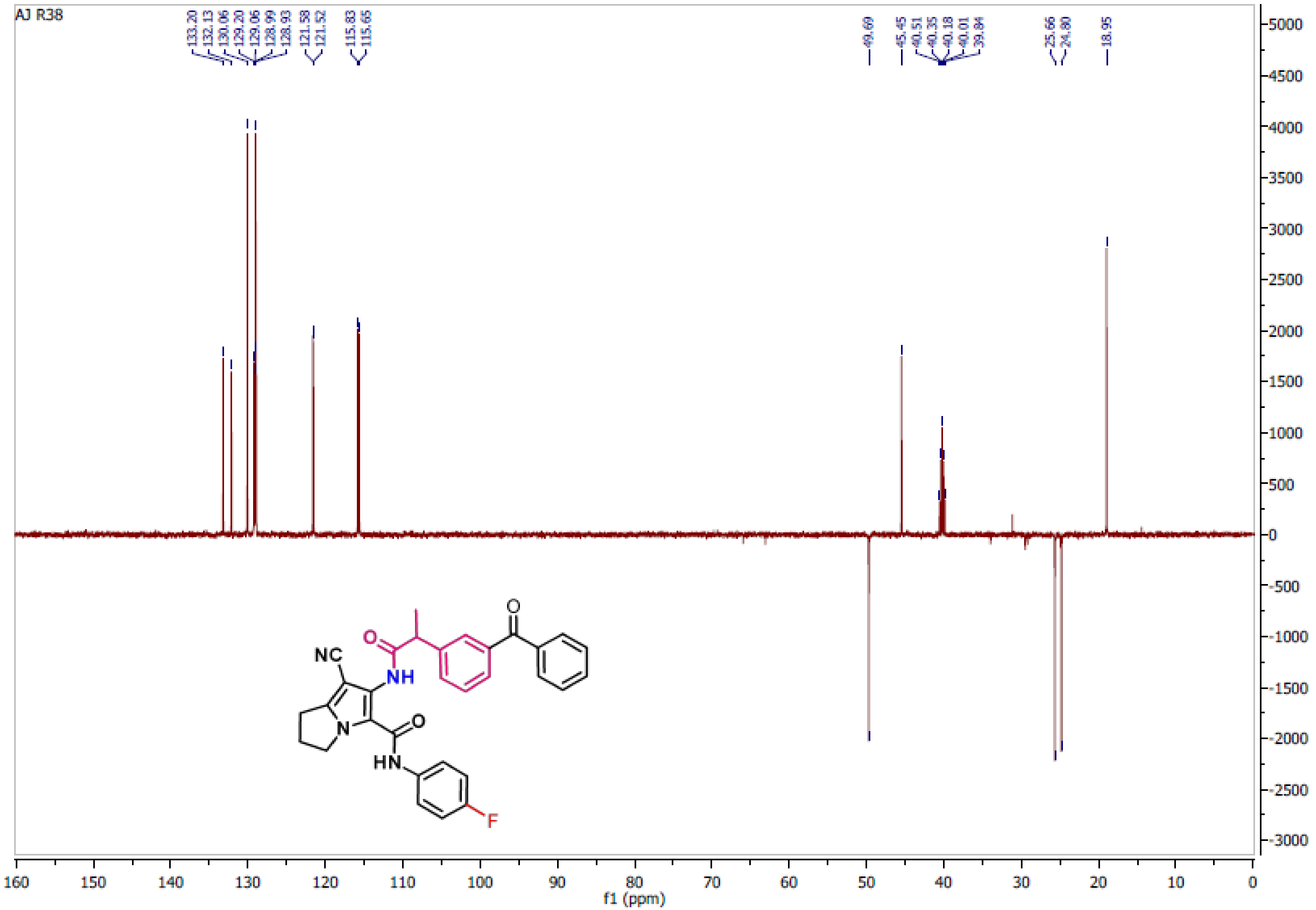

Fig. S45. DEPT C135 (DMSO, 125 MHz, *δ* ppm) of compound **8d** **(zoom on aliphatic Cs)**

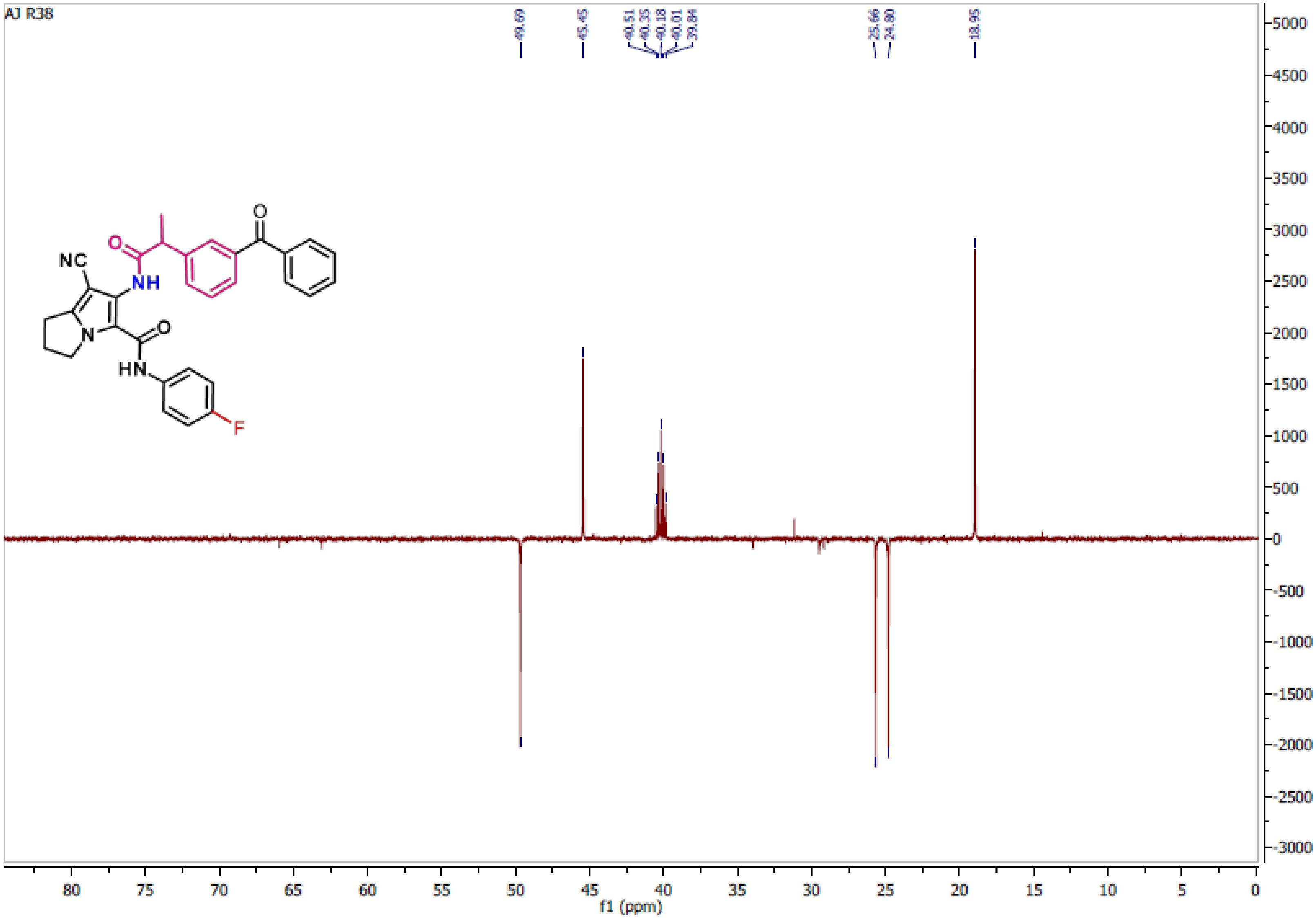

Supplementary materials

Fig. S46. DEPT C^{135} (DMSO, 125 MHz, δ ppm) of compound **8d** **(zoom on aromatic Cs)**

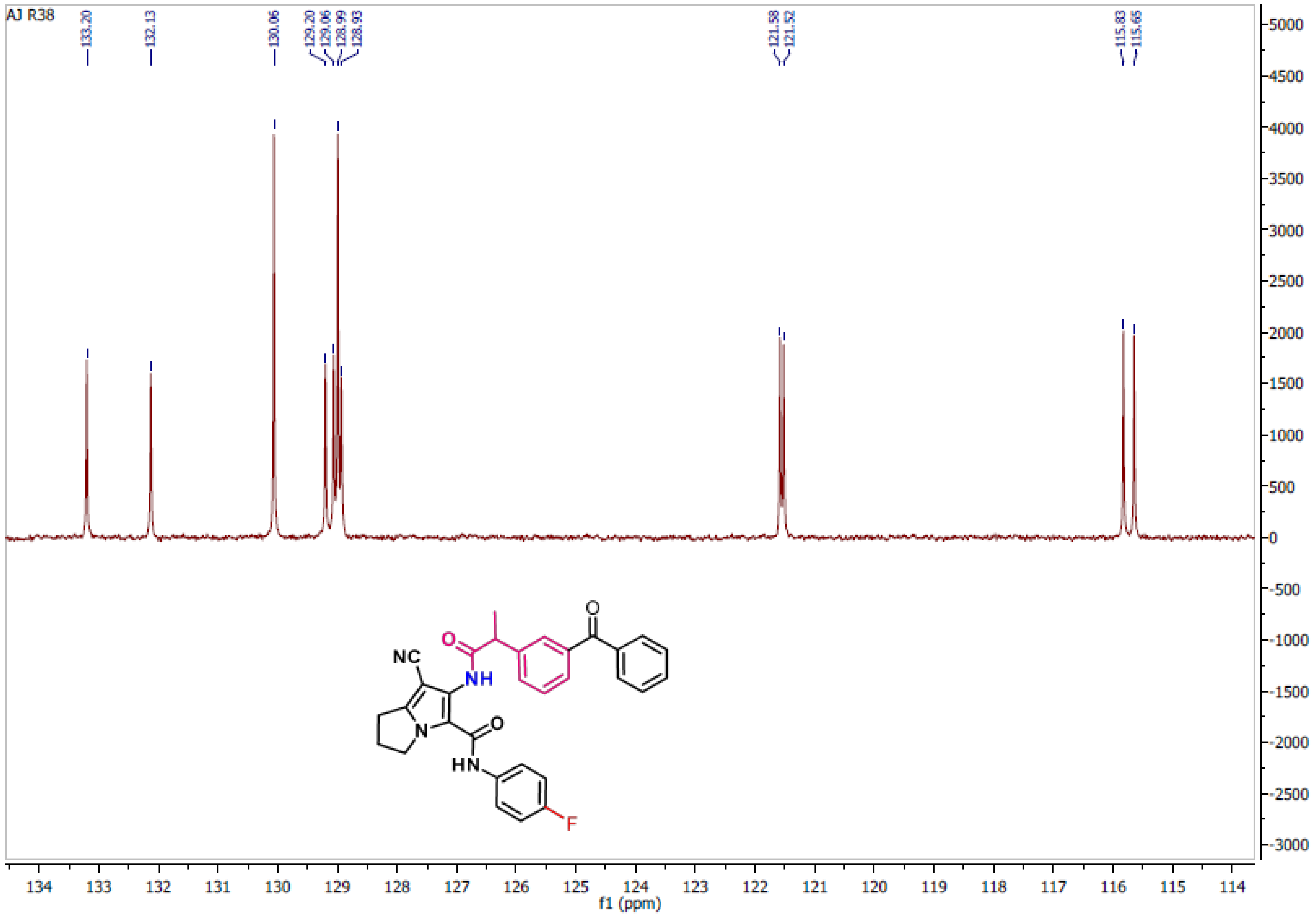

Fig. S47. DEPT C135 (DMSO, 125 MHz, δ ppm) of compound **8d** **(zoom on aromatic Cs)**

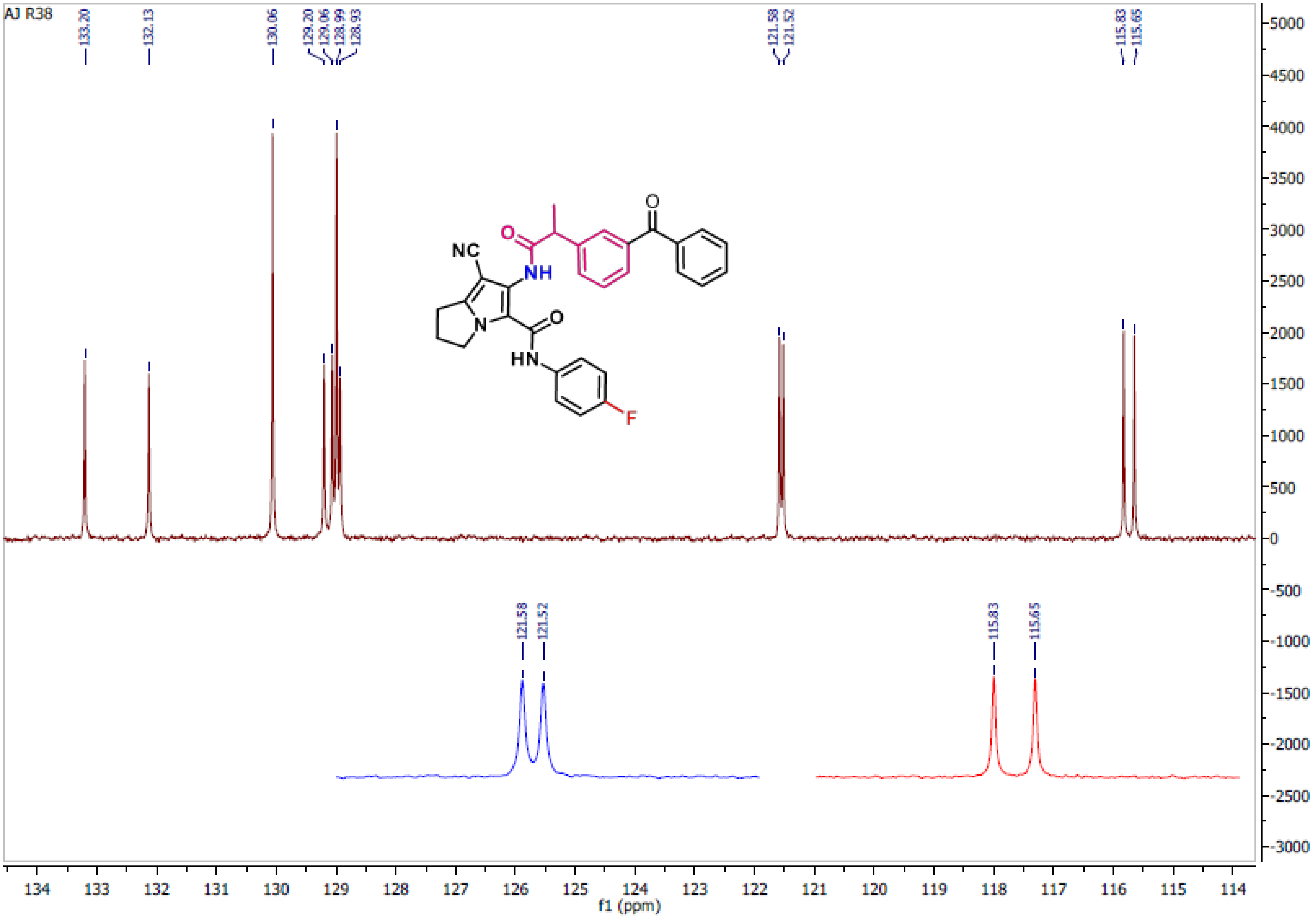

Supplementary materials

Fig. S48. ^{1}H-NMR (DMSO, 500 MHz, δ ppm) spectrum of compound **8e**

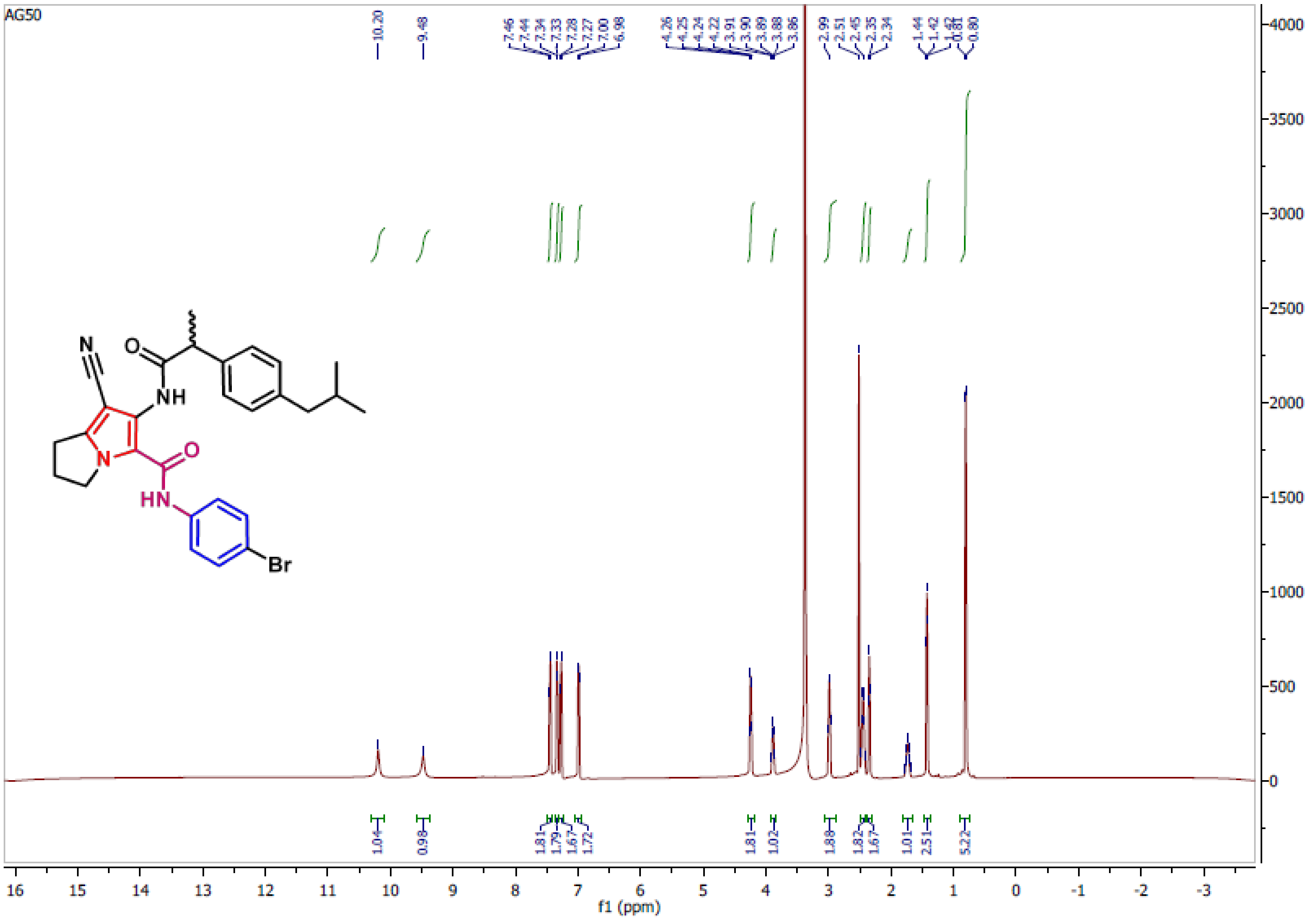

Fig. S49. ^{13}C-NMR (DMSO, 125 MHz, δ ppm) spectrum of compound **8e**

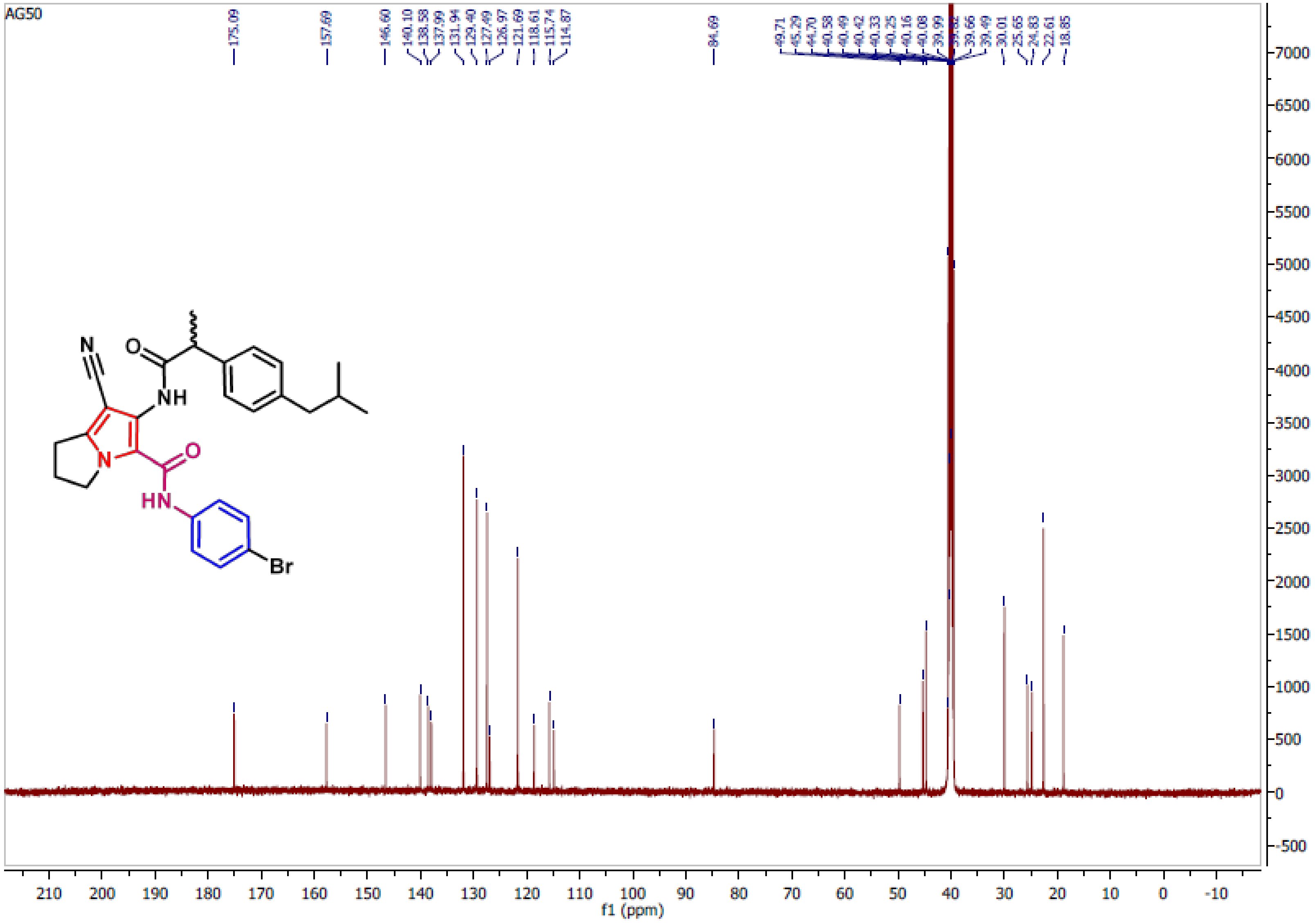

Fig. S50. ^{13}C-NMR (DMSO, 125 MHz, δ ppm) spectrum of compound **8e** **(zoom on aliphatic Cs)**

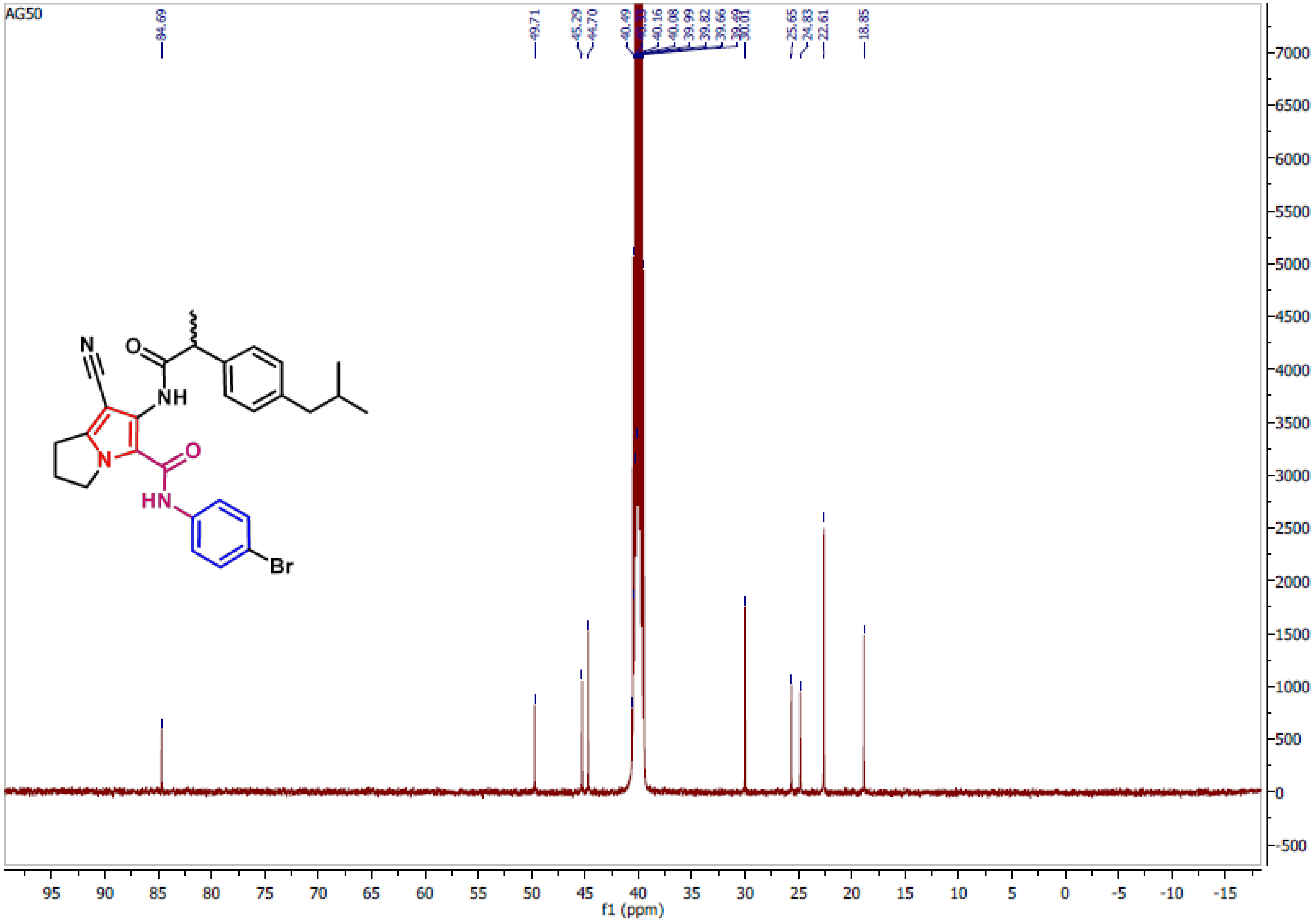

Fig. S51. ^{13}C-NMR (DMSO, 125 MHz, δ ppm) spectrum of compound **8e** **(zoom on aromatic Cs)**

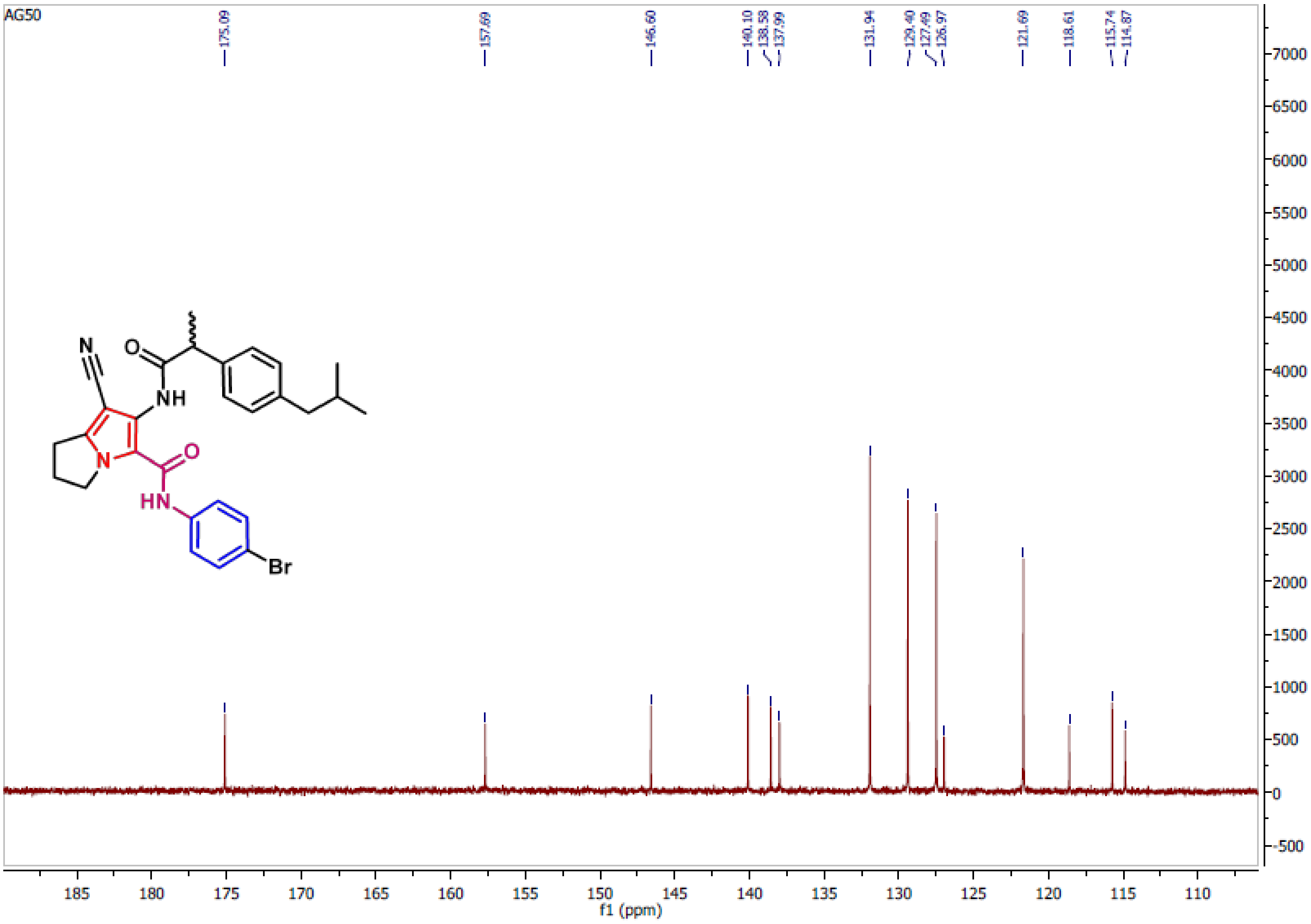

Fig. S52. ^{1}H-NMR (DMSO-d_6, 500 MHz, δ ppm) spectrum of compound **8f**

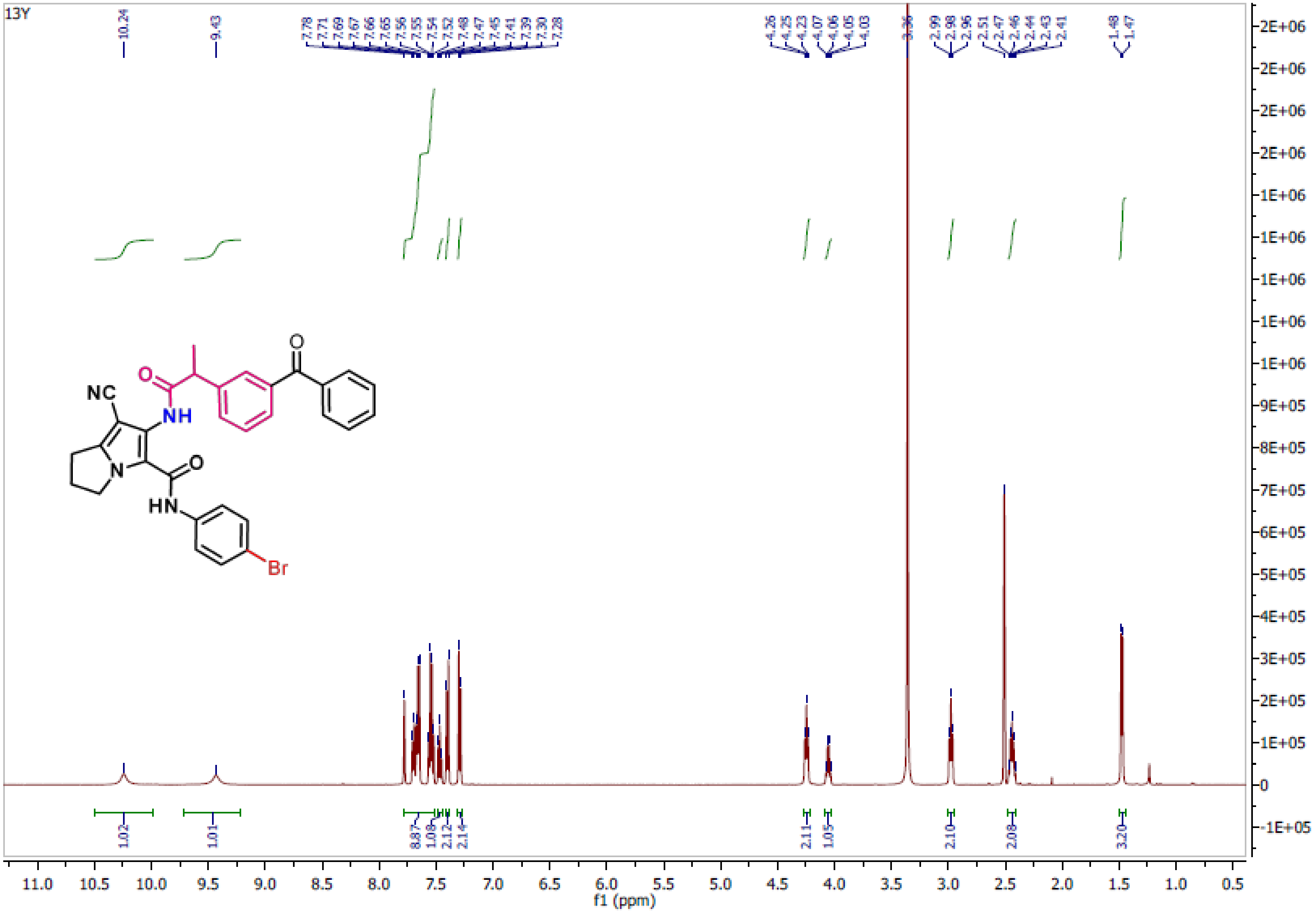

Fig. S53. ^{1}H-NMR (DMSO-d_6, 500 MHz, δ ppm) spectrum of compound **8f** **(zoom on aliphatic Hs)**

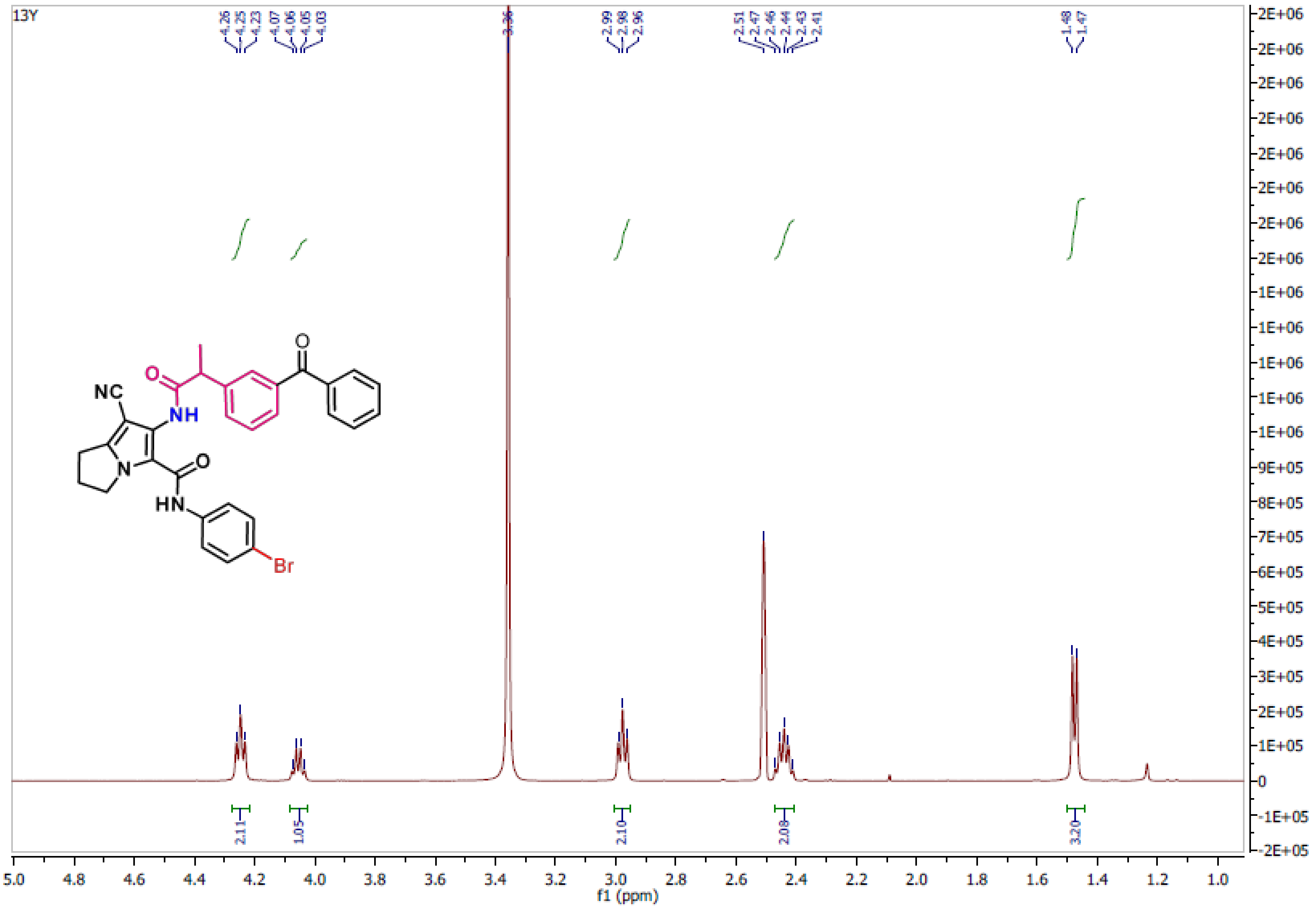

Fig. S54. ^{1}H-NMR (DMSO-d_6, 500 MHz, δ ppm) spectrum of compound **8f** **(zoom on aromatic Hs)**

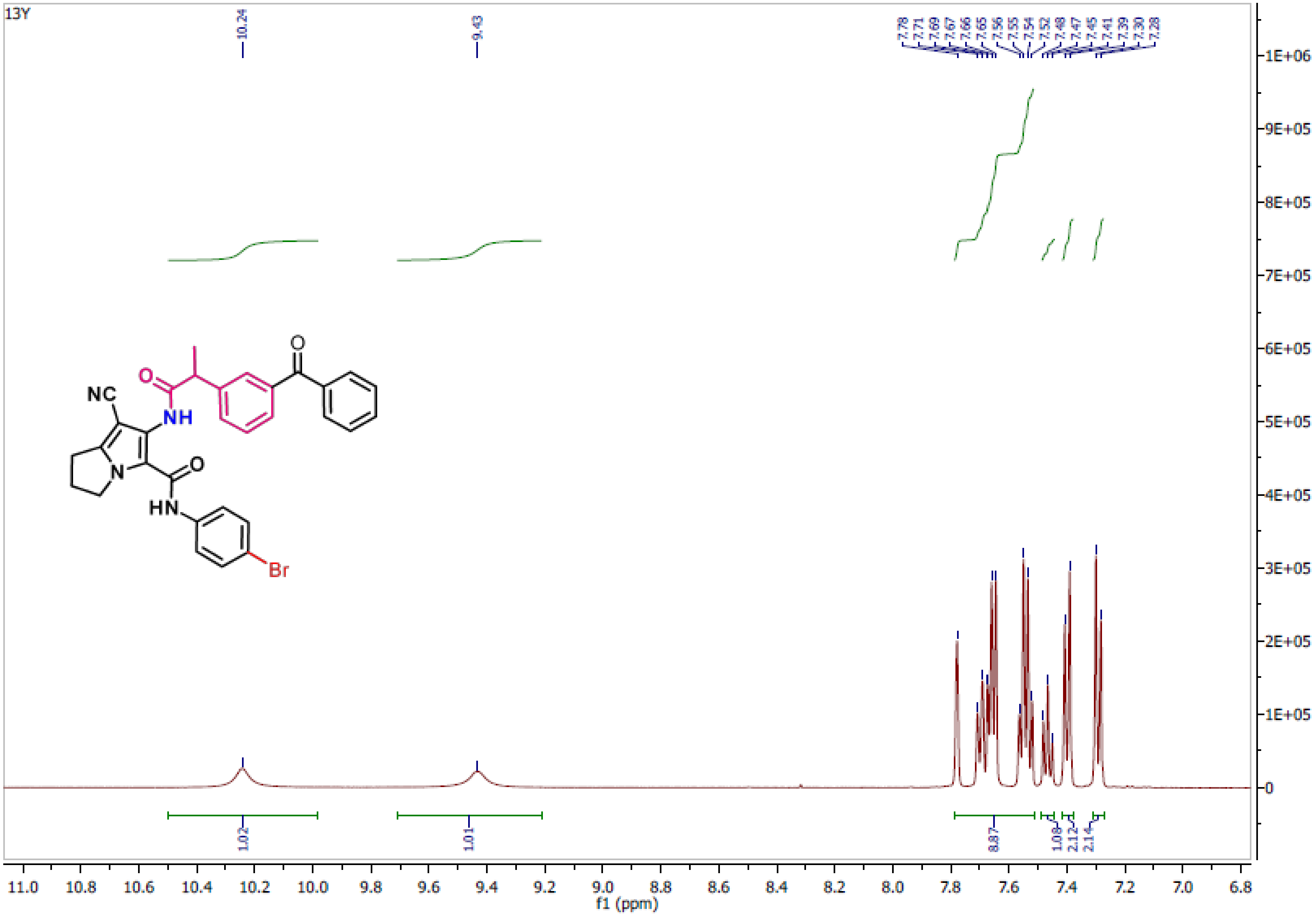

Fig. S55. ^{13}C-NMR (DMSO, 125 MHz, δ ppm) spectrum of compound **8f**

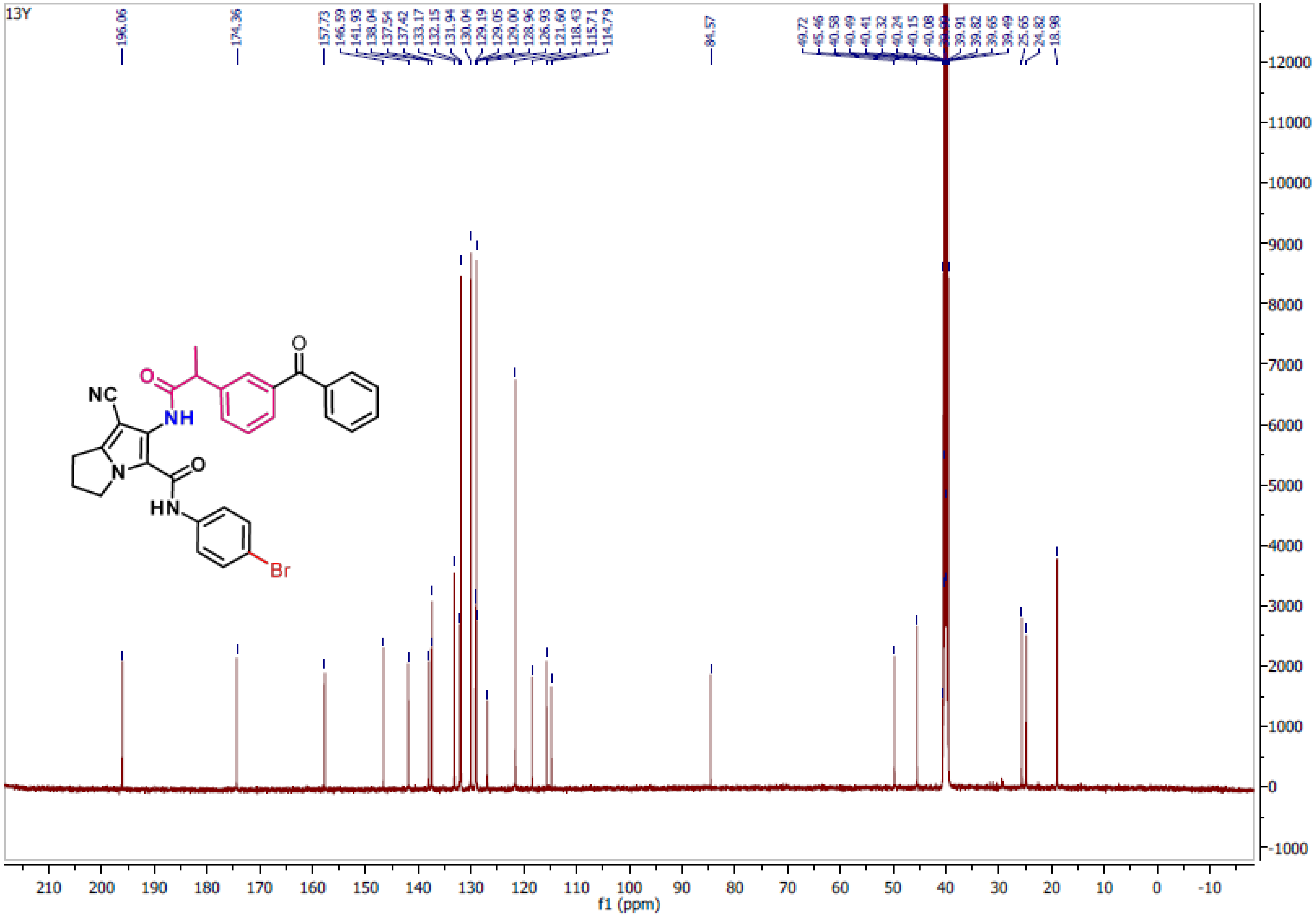

Fig. S56. ^{13}C-NMR (DMSO, 125 MHz, δ ppm) spectrum of compound **8f** **(zoom on aliphatic Cs)**

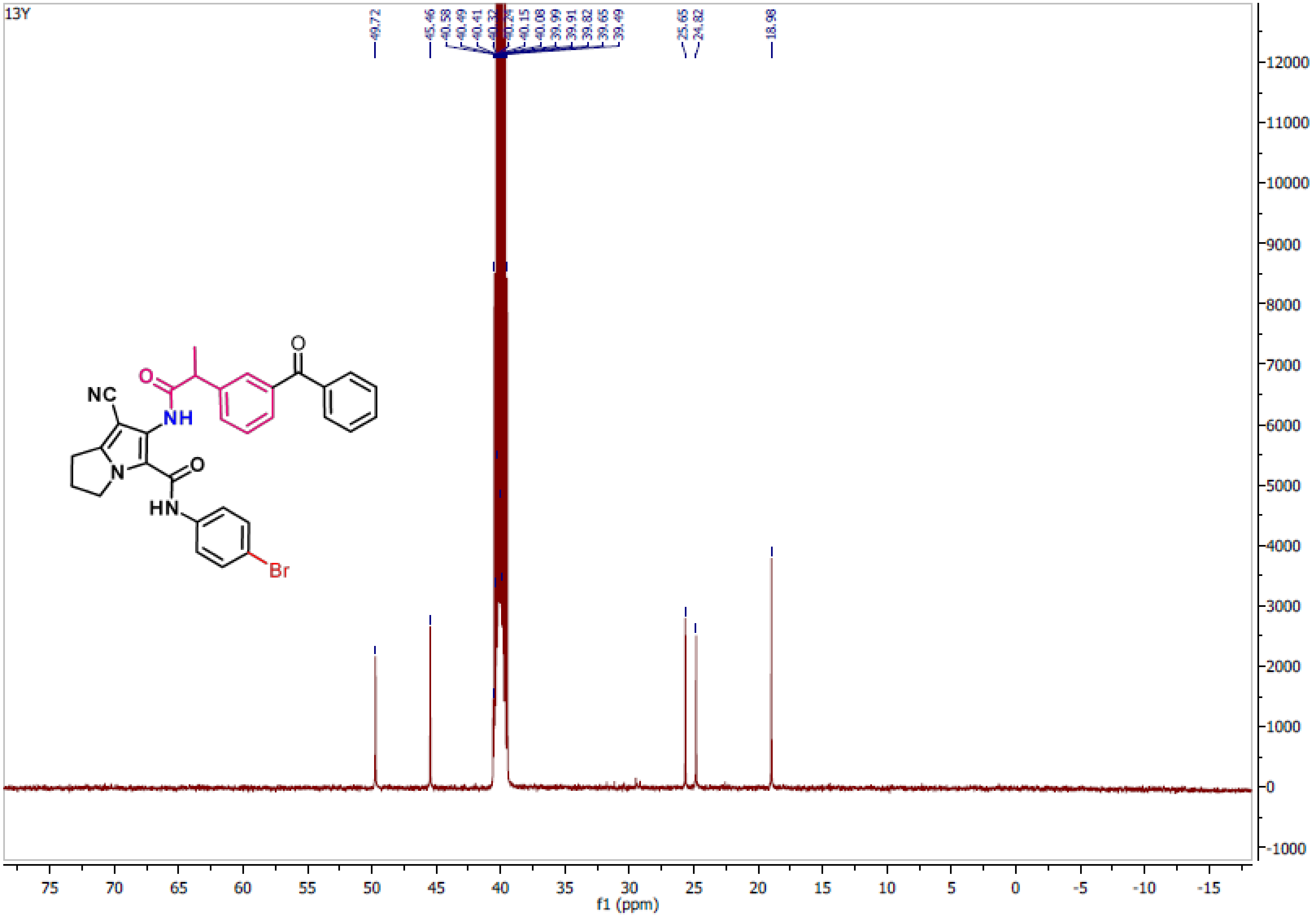

Fig. S57. ^{13}C-NMR (DMSO, 125 MHz, δ ppm) spectrum of compound **8f** **(zoom on aromatic Cs)**

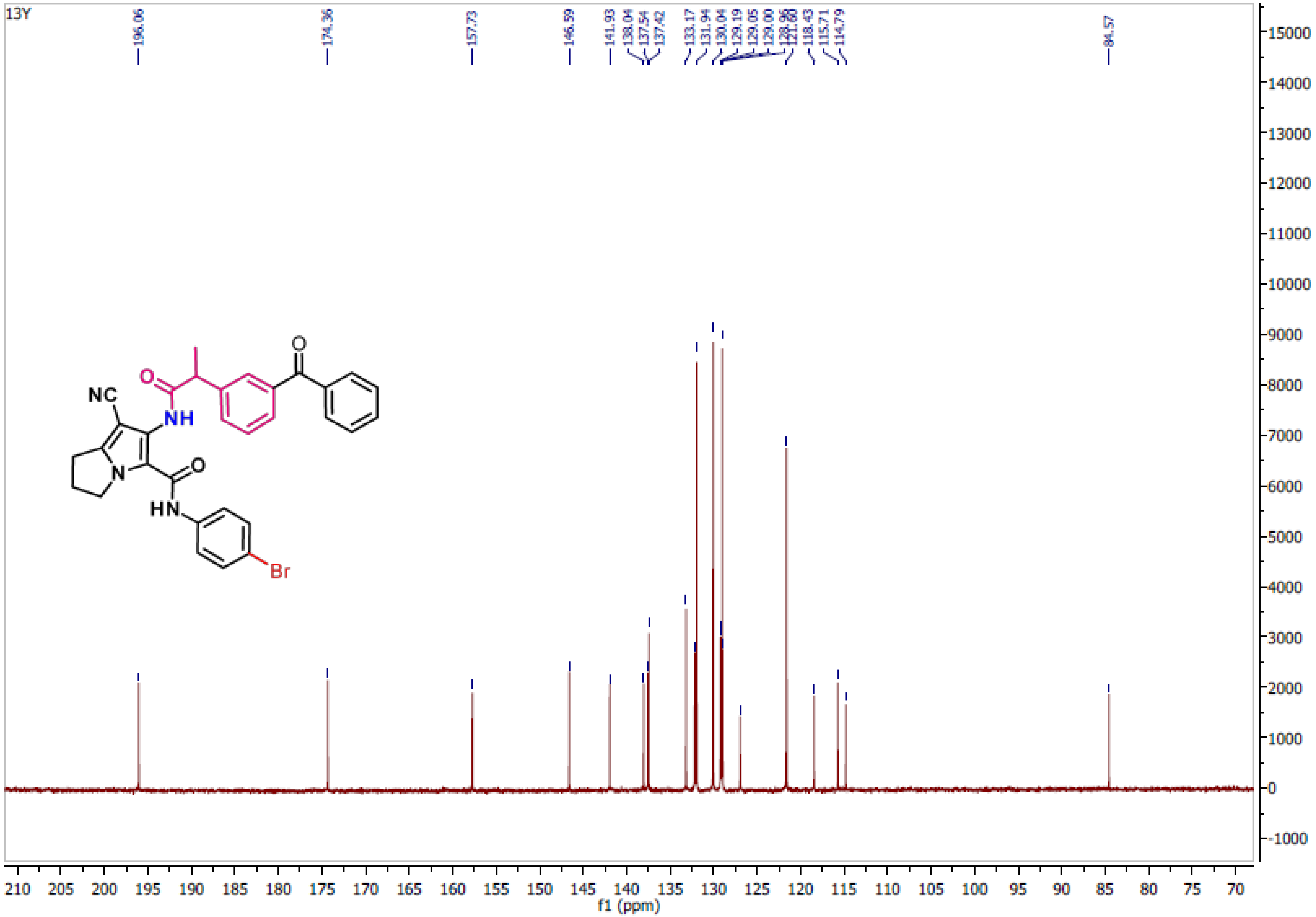

Fig. S58. DEPT C^{135} (DMSO, 125 MHz, δ ppm) of compound **8f**

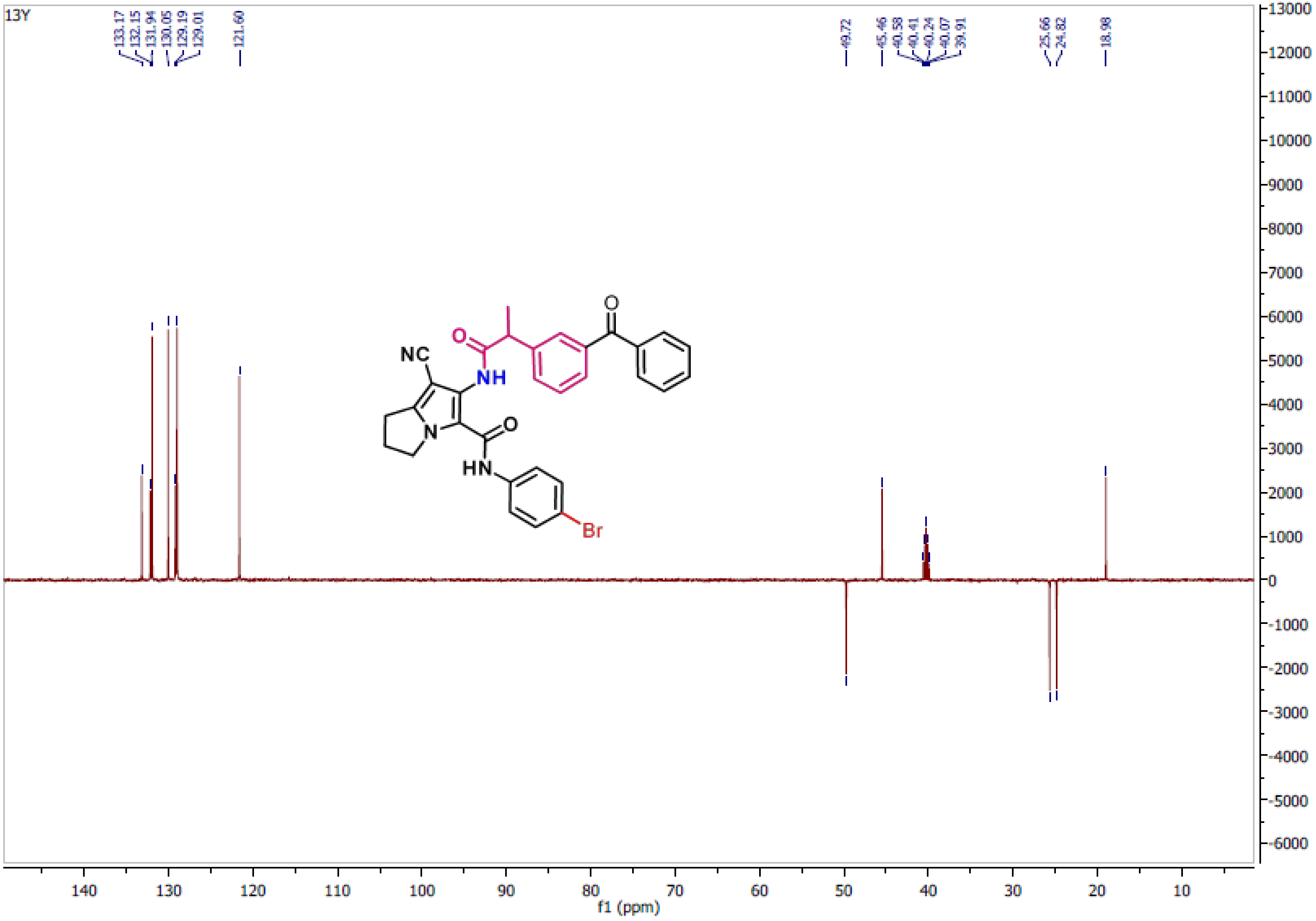

Fig. S59. ^{1}H-NMR (DMSO-d_6, 500 MHz, δ ppm) spectrum of compound **8g**

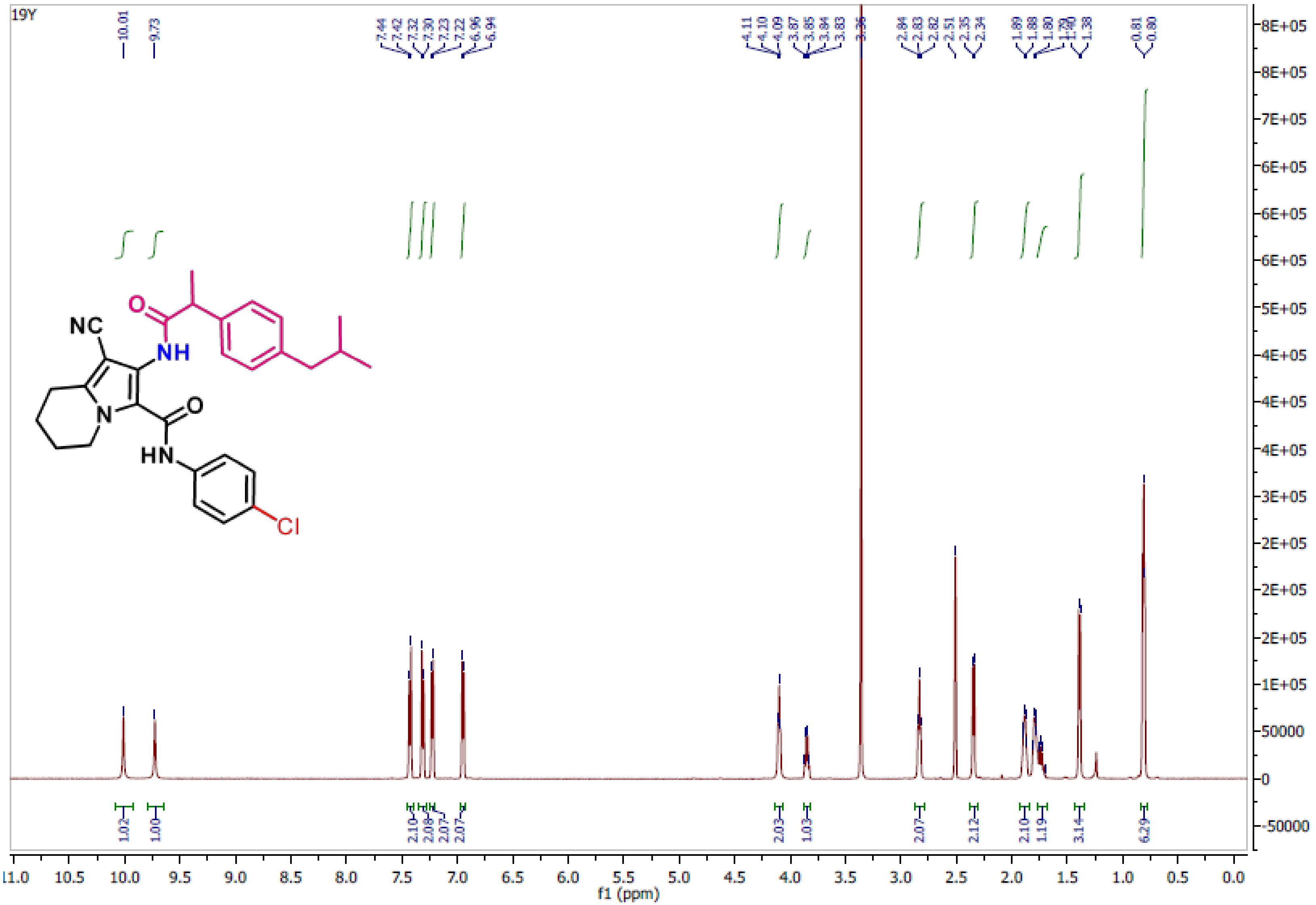

Fig. S60. ^{1}H-NMR (DMSO-d_6, 500 MHz, δ ppm) spectrum of compound **8g** **(zoom on aliphatic Hs)**

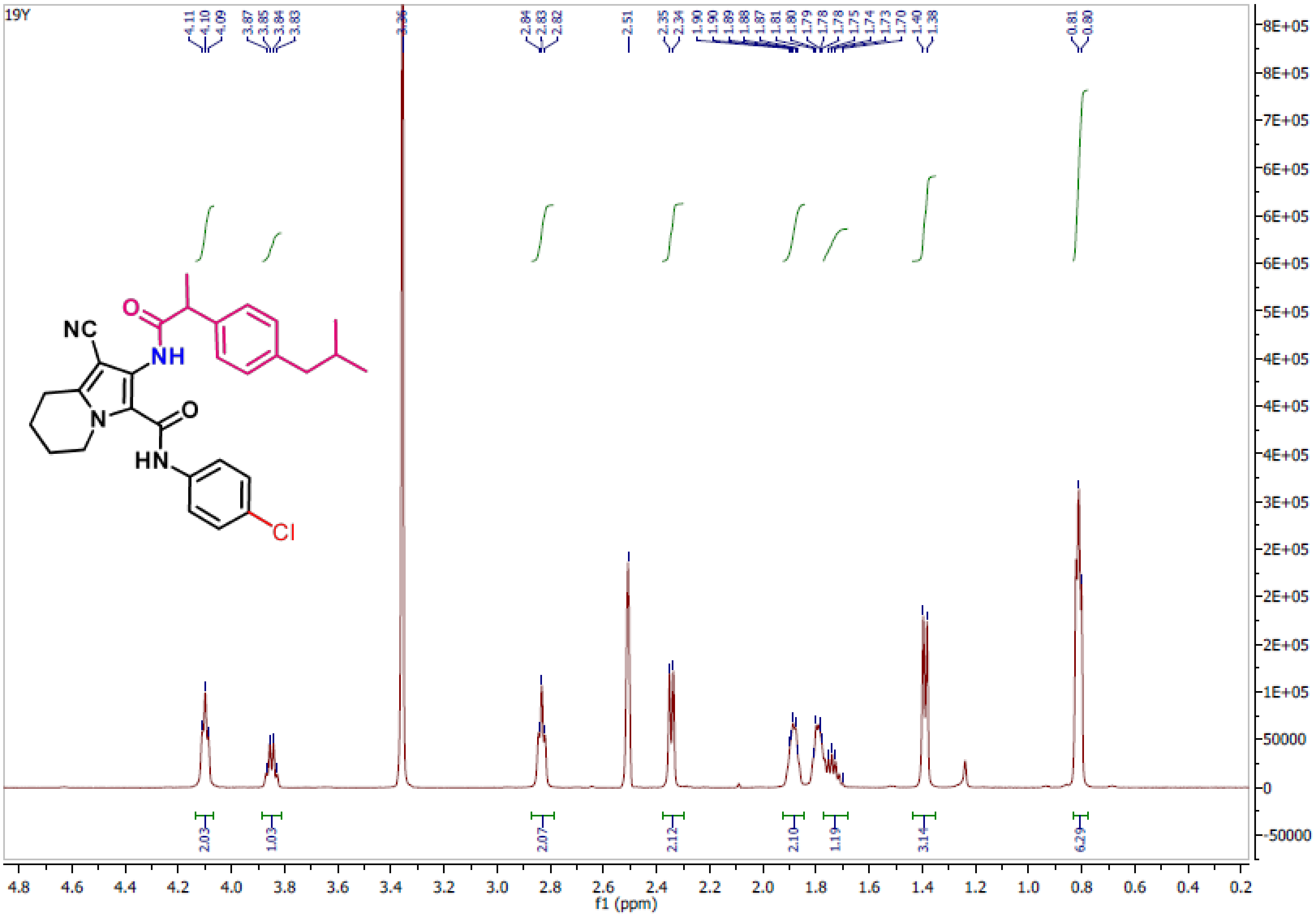

Fig. S61. ^{1}H-NMR (DMSO-d_6, 500 MHz, δ ppm) spectrum of compound **8g** **(zoom on aromatic Hs)**

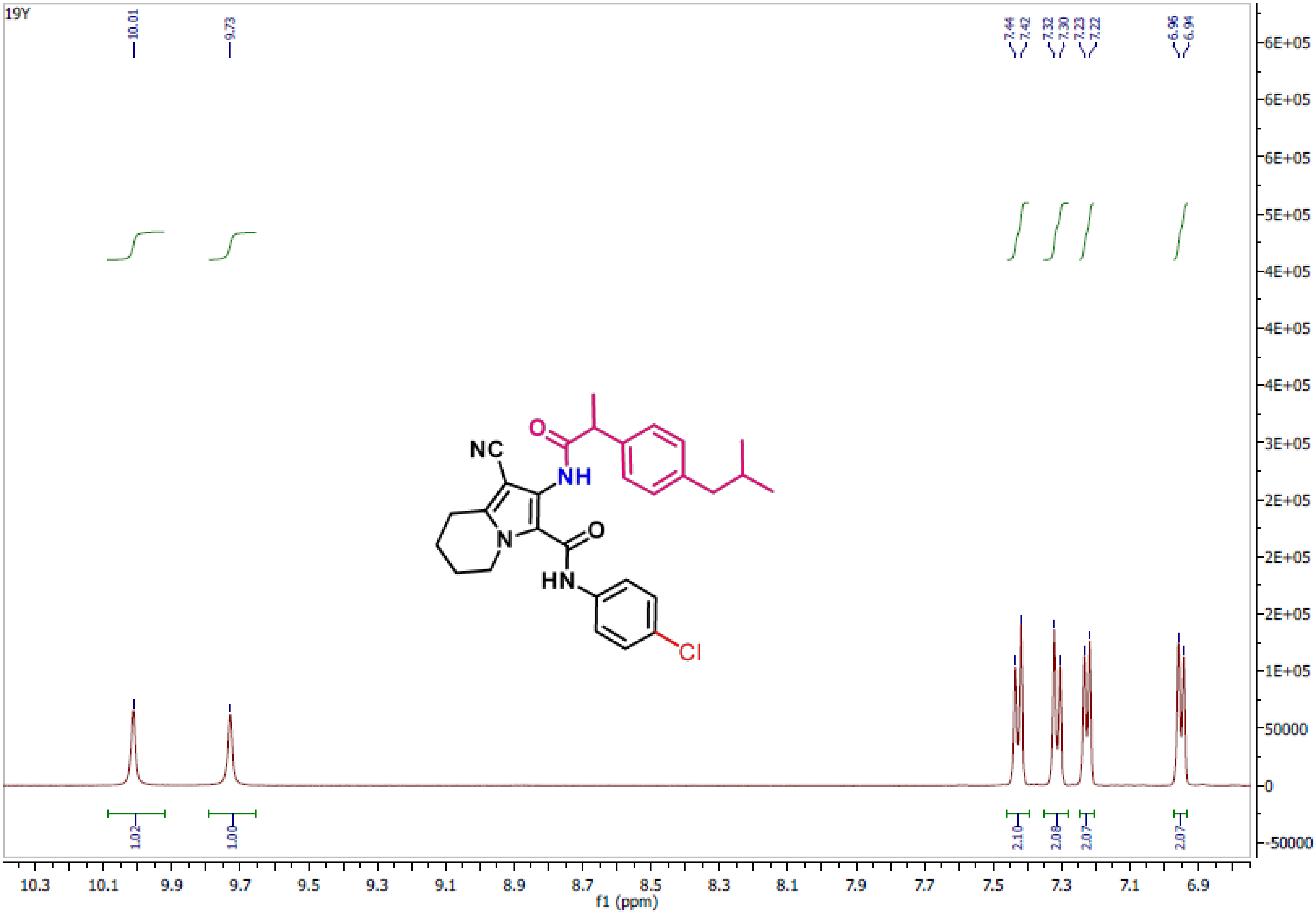

Fig. S62. ^{13}C-NMR (DMSO, 125 MHz, δ ppm) spectrum of compound **8g**

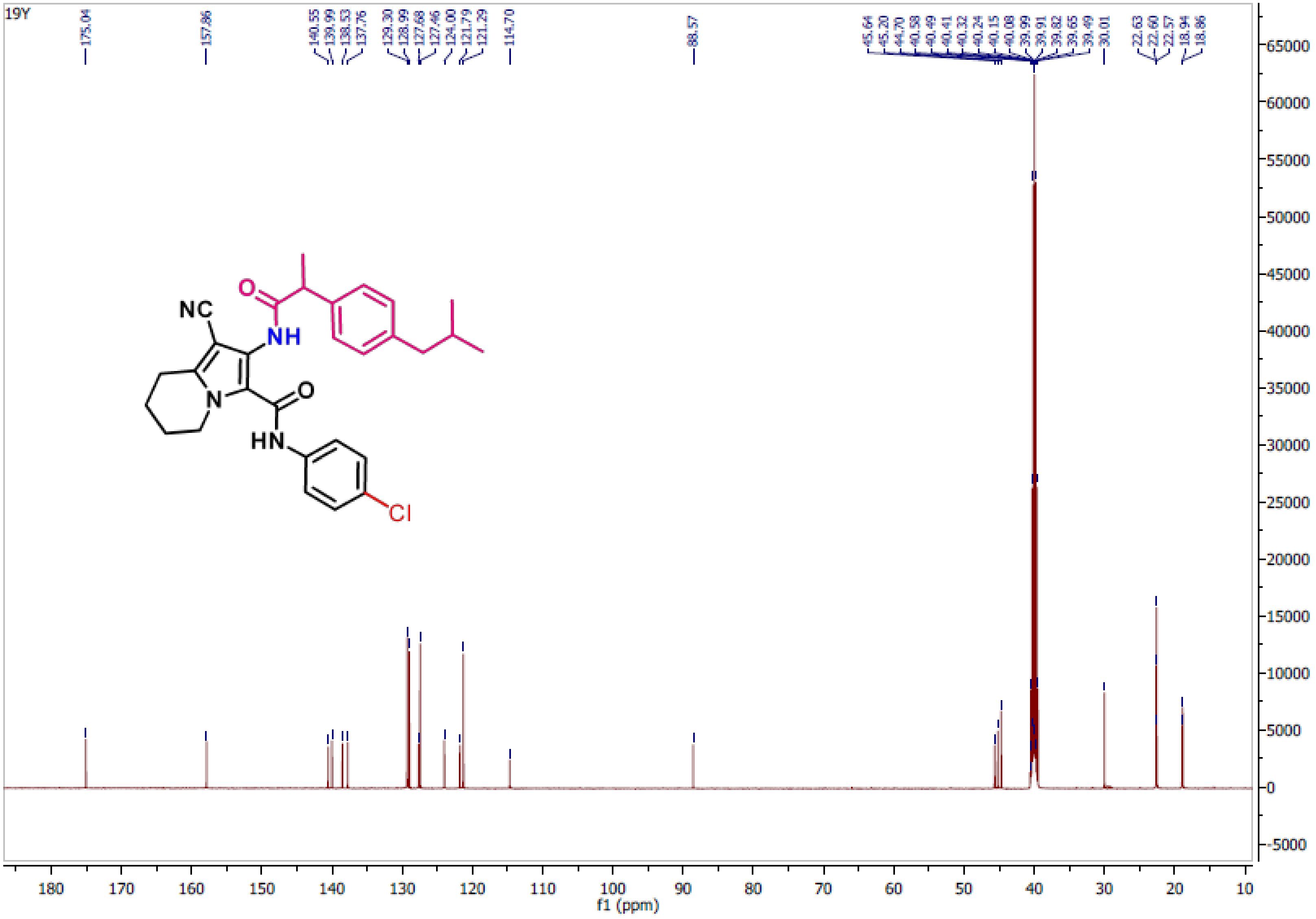

Fig. S63. ^{13}C-NMR (DMSO, 125 MHz, δ ppm) spectrum of compound **8g** **(zoom on aliphatic Cs)**

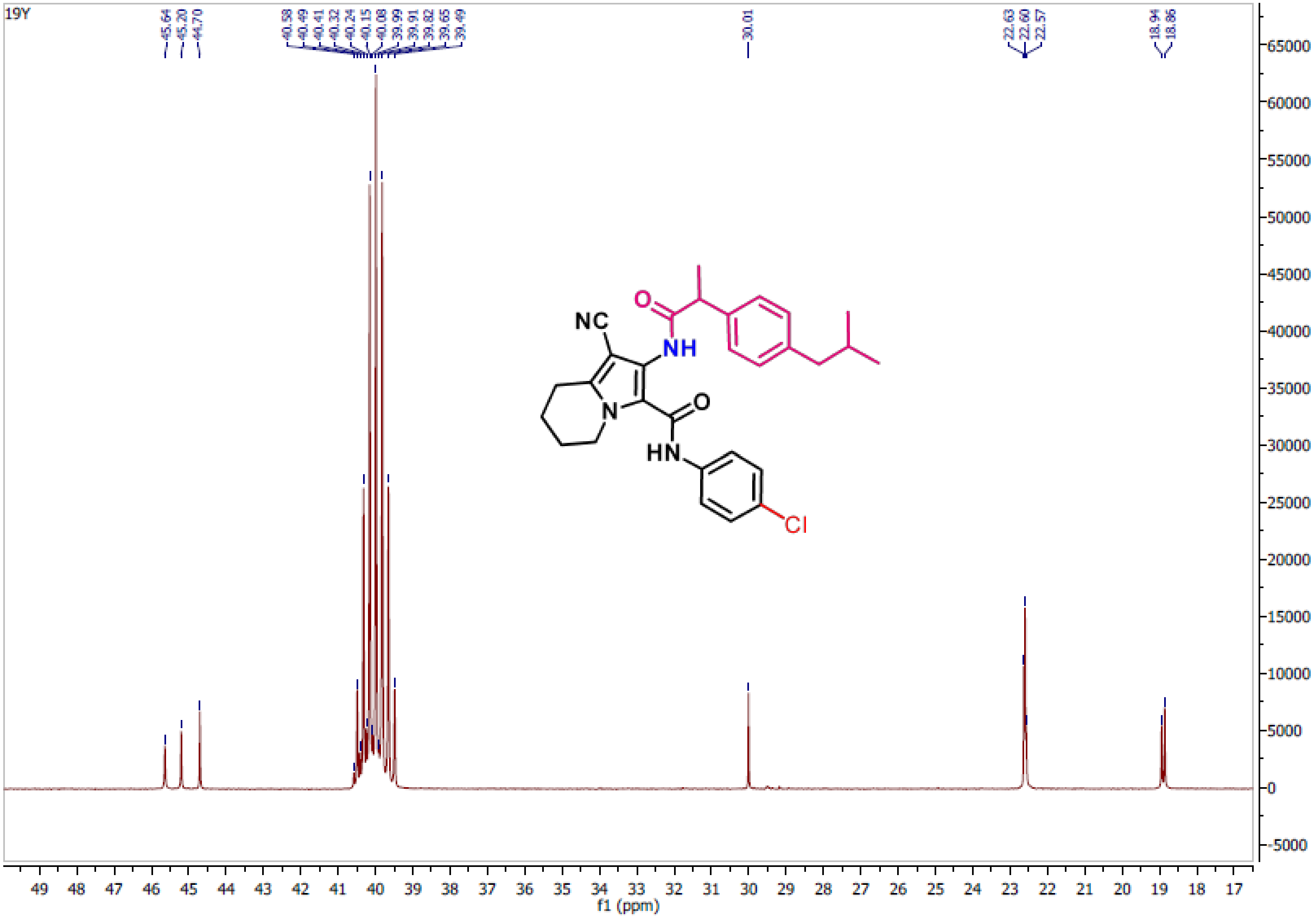

Fig. S64. ^{13}C-NMR (DMSO, 125 MHz, δ ppm) spectrum of compound **8g** **(zoom on aliphatic Cs)**

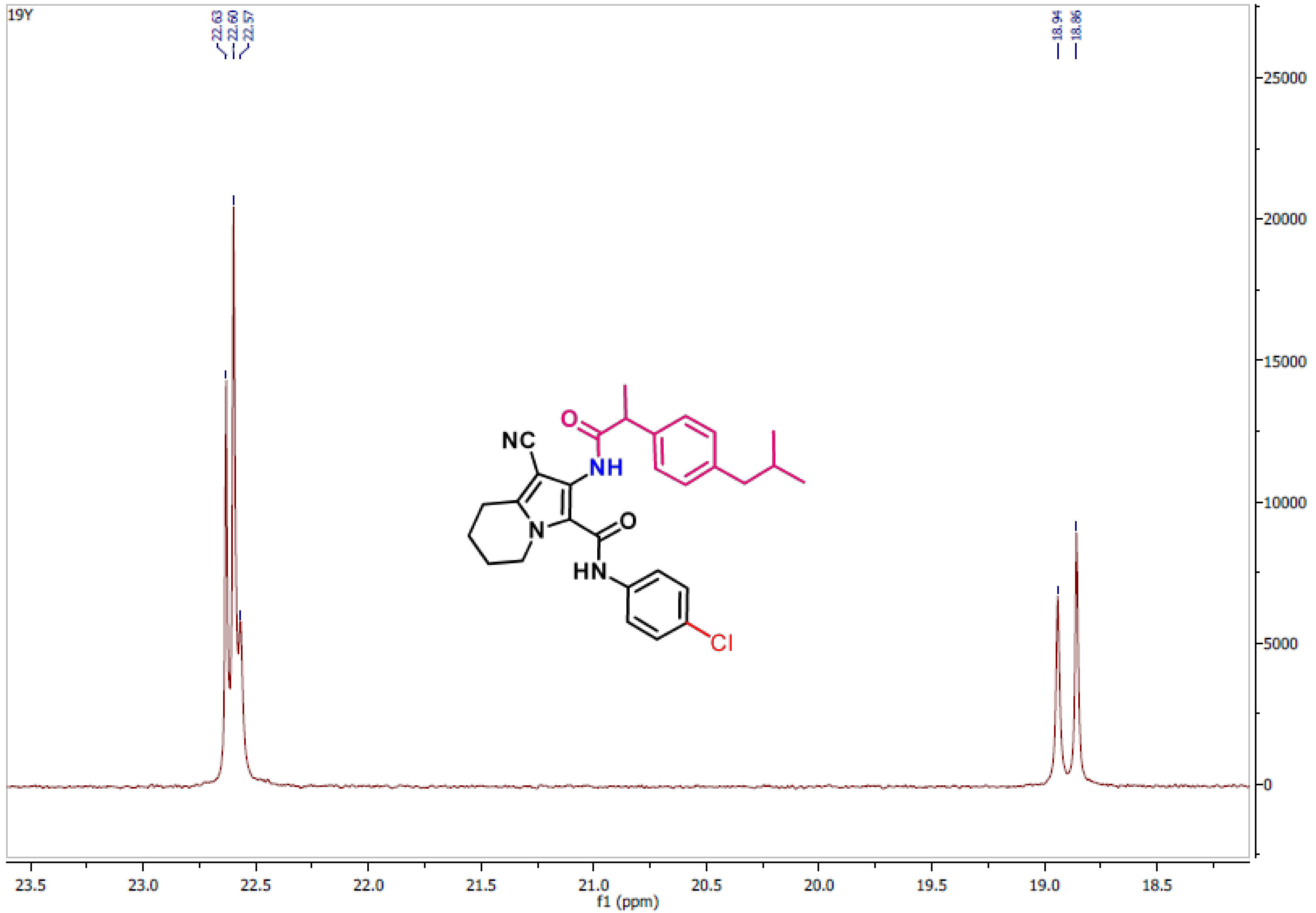

Fig. S65. ^{13}C-NMR (DMSO, 125 MHz, δ ppm) spectrum of compound **8g** **(zoom on aromatic Cs)**

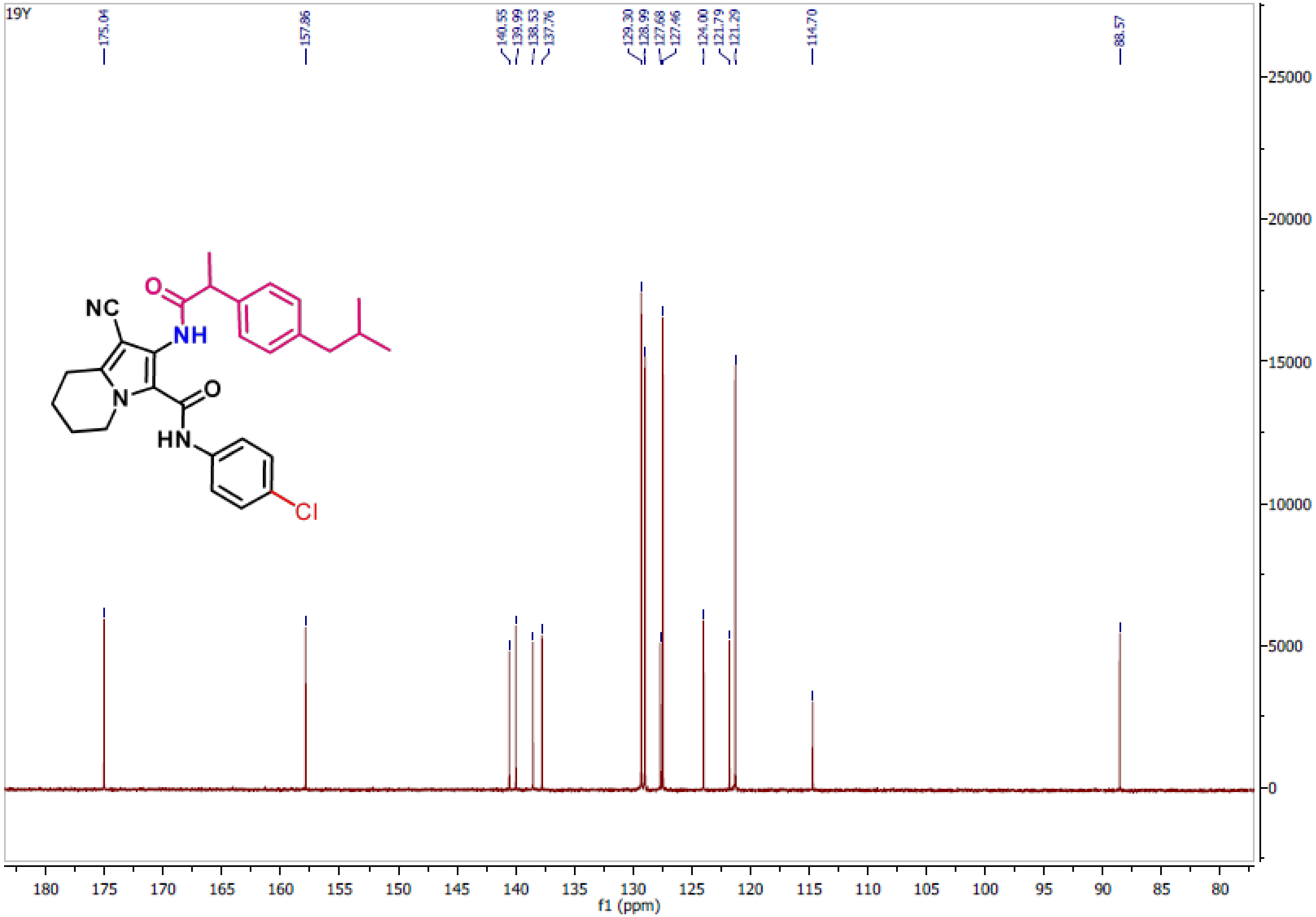

Fig. S66. DEPT C^{135} (DMSO, 125 MHz, δ ppm) of compound **8g**

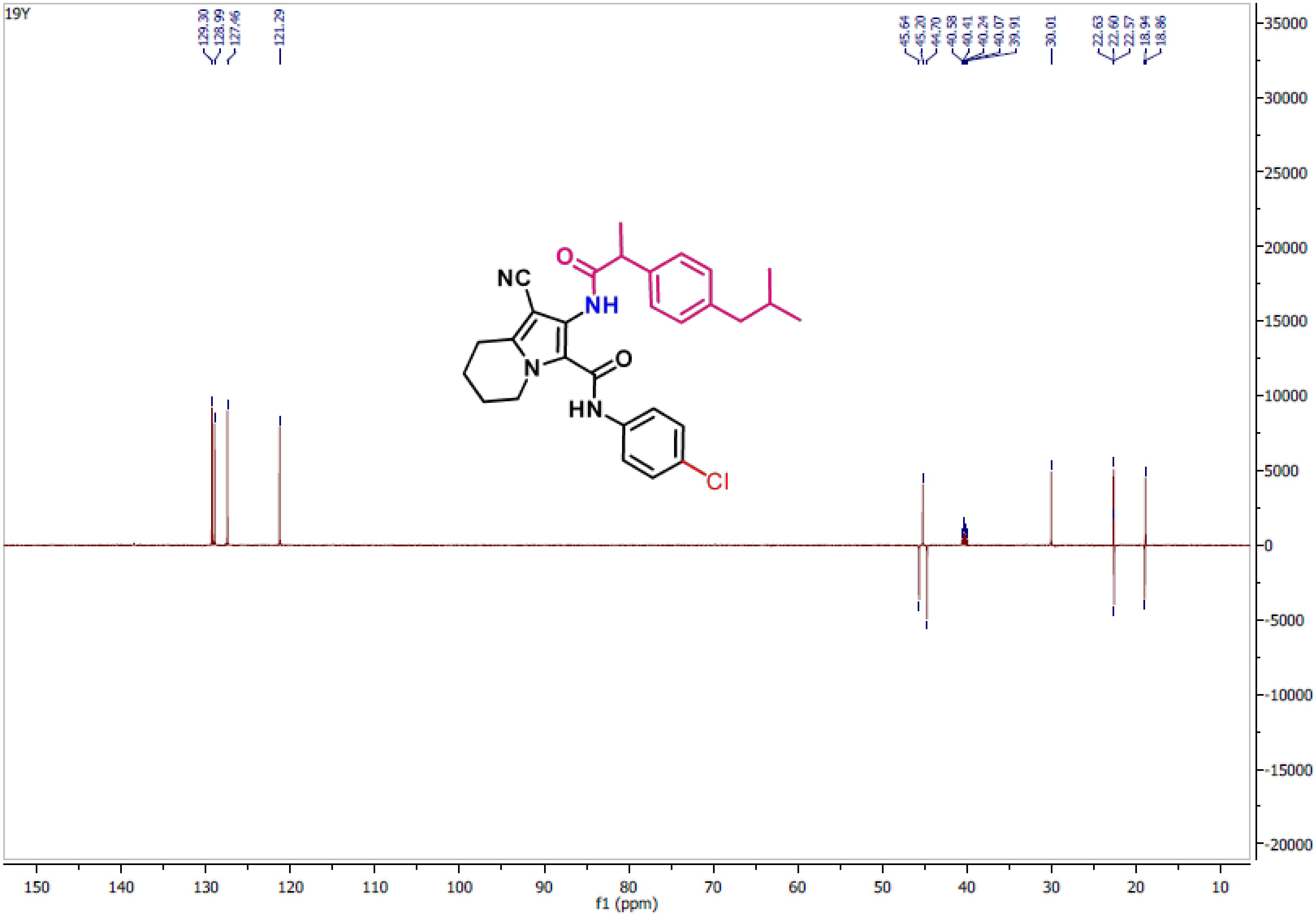

Fig. S67. ^{1}H-NMR (DMSO-d_6, 500 MHz, δ ppm) spectrum of compound **8h**

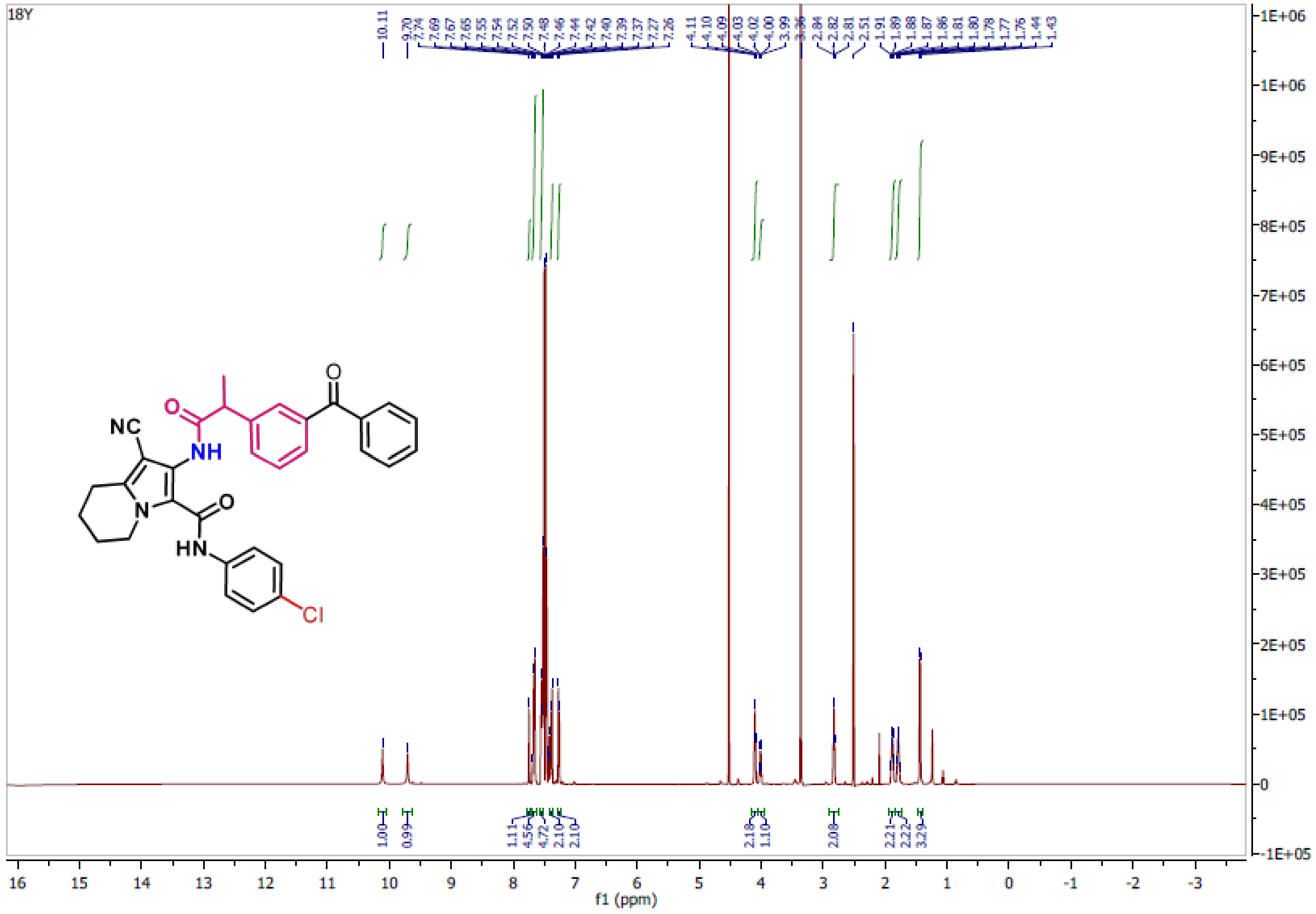

Fig. S68. ^{1}H-NMR (DMSO-d_6, 500 MHz, δ ppm) spectrum of compound **8h** **(zoom on aliphatic Hs)**

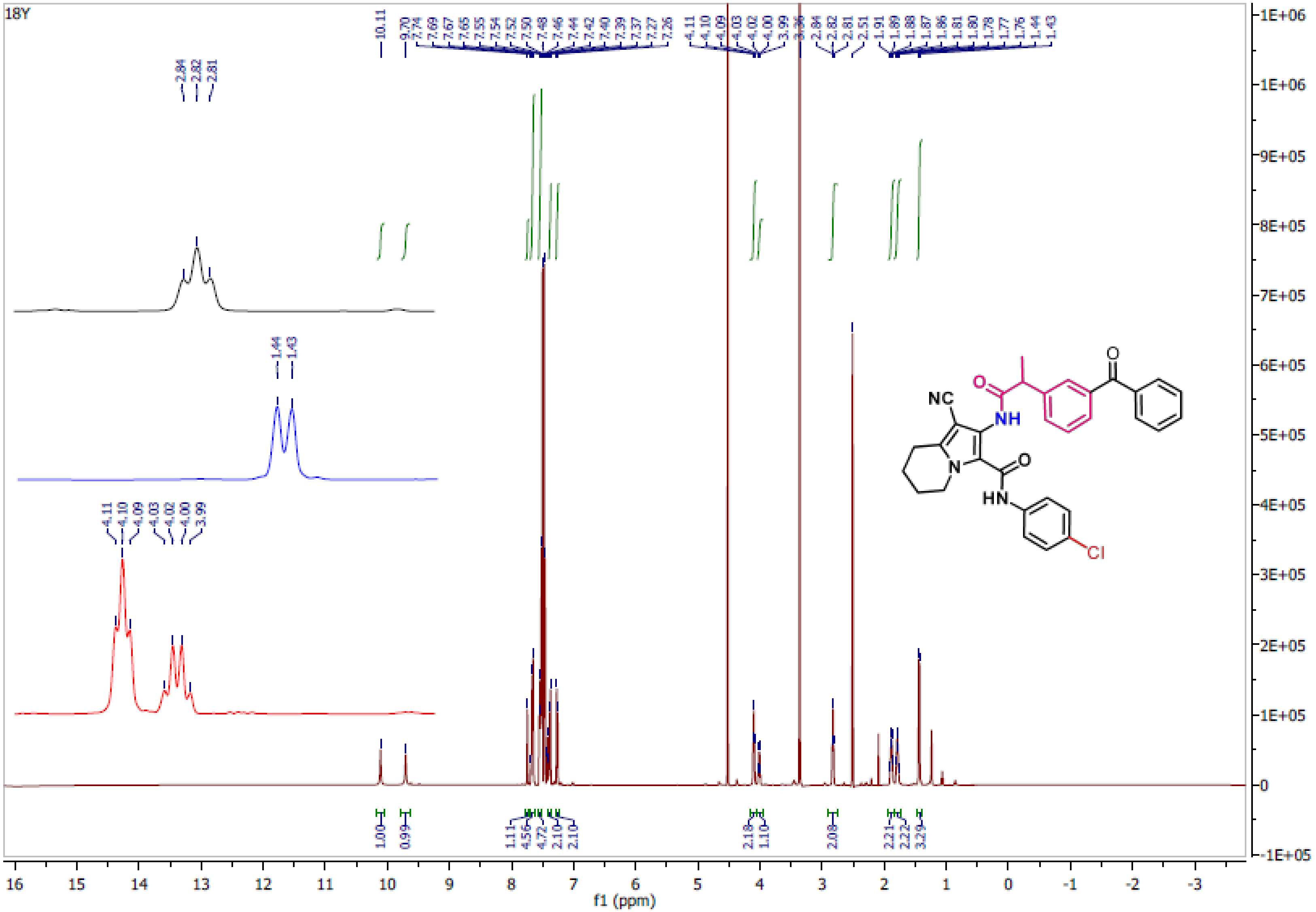

Fig. S69. ^{13}C-NMR (DMSO, 125 MHz, δ ppm) spectrum of compound **8h**

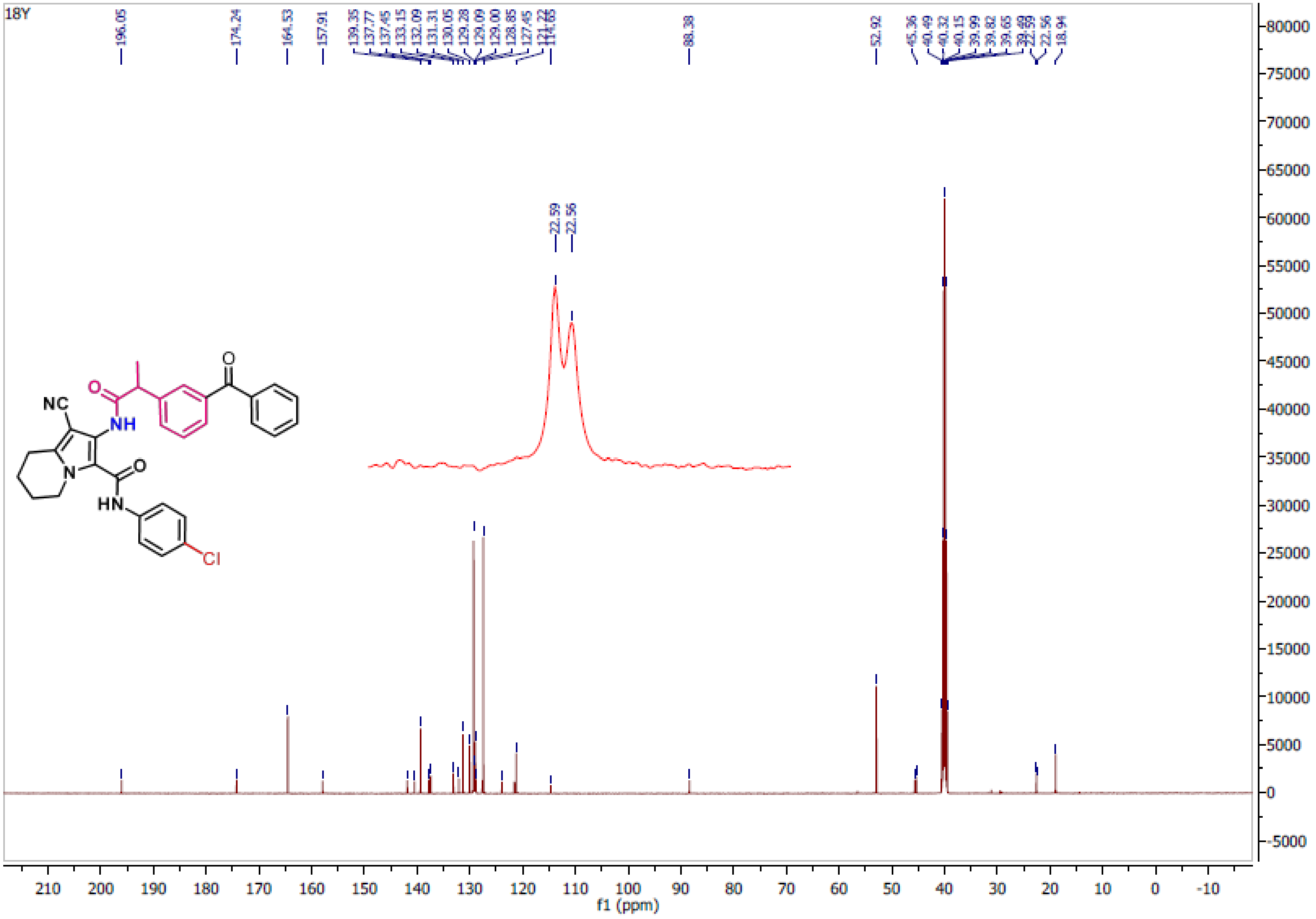

Fig. S70. DEPT C^{135} (DMSO, 125 MHz, δ ppm) of compound **8h**

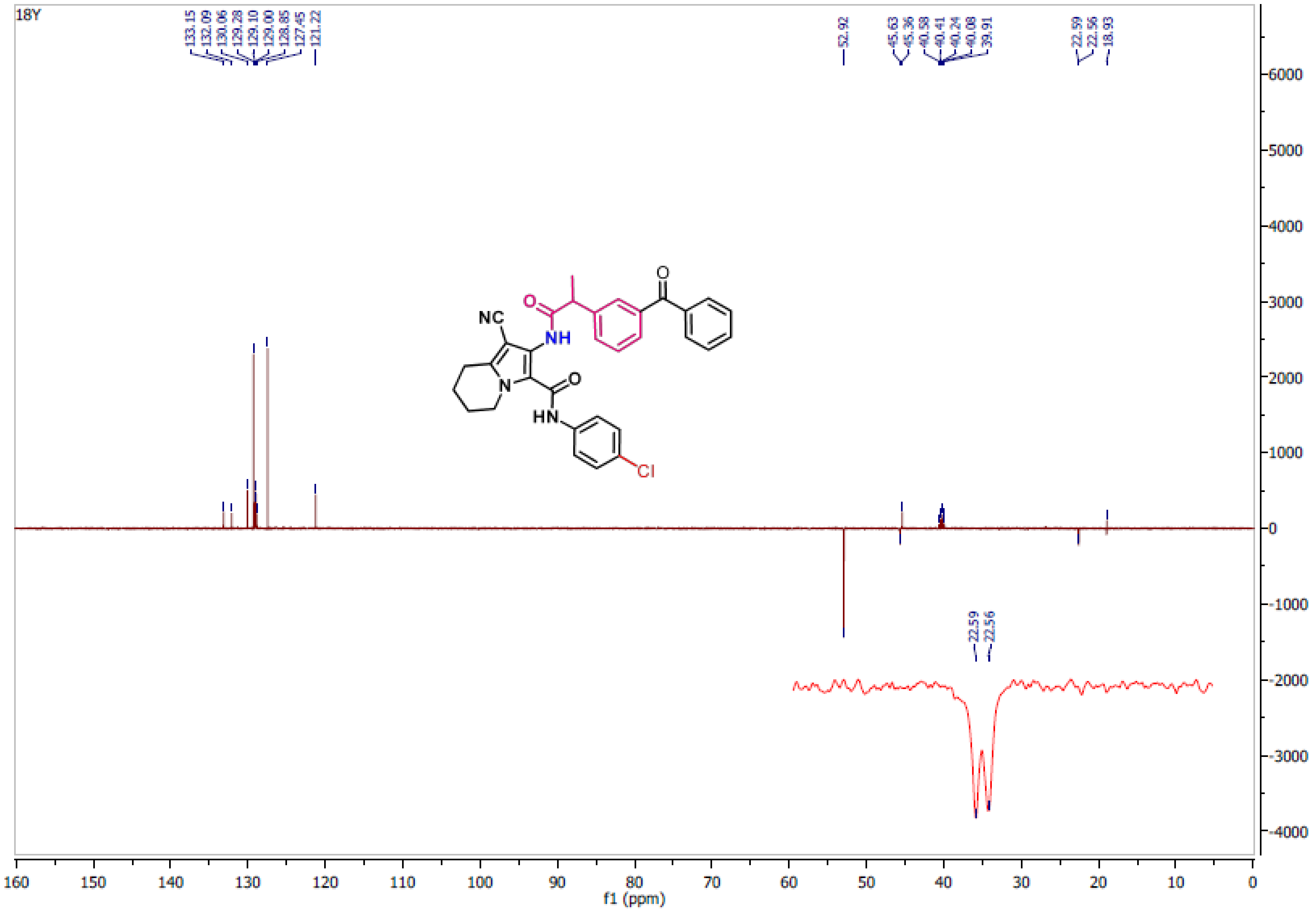

Fig. S71. ^{1}H-NMR ($CDCl_3$, 500 MHz, δ ppm) spectrum of compound **8i**

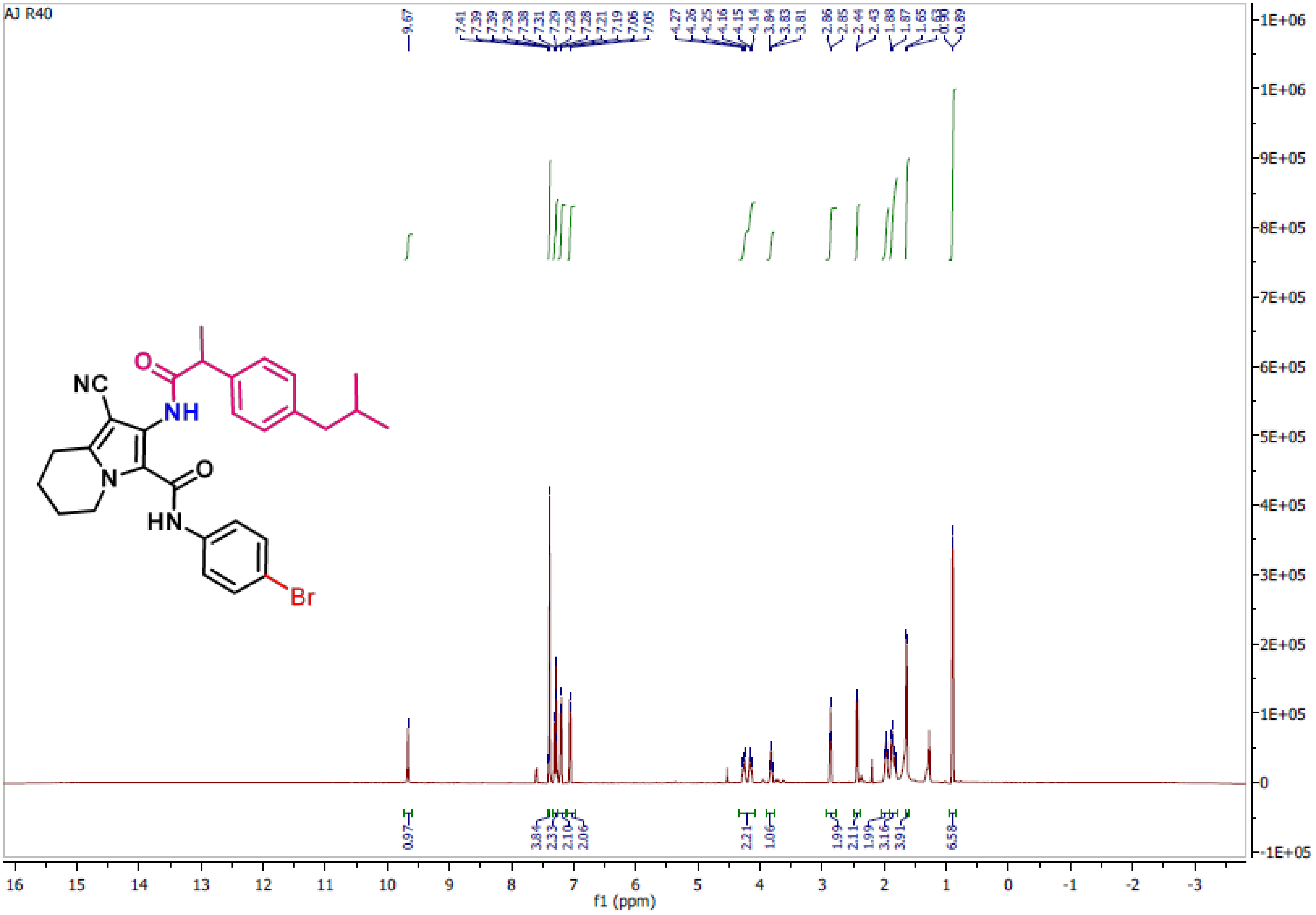

Fig. S72. ^{1}H-NMR ($CDCl_3$, 500 MHz, δ ppm) spectrum of compound **8i** **(zoom on aromatic Hs)**

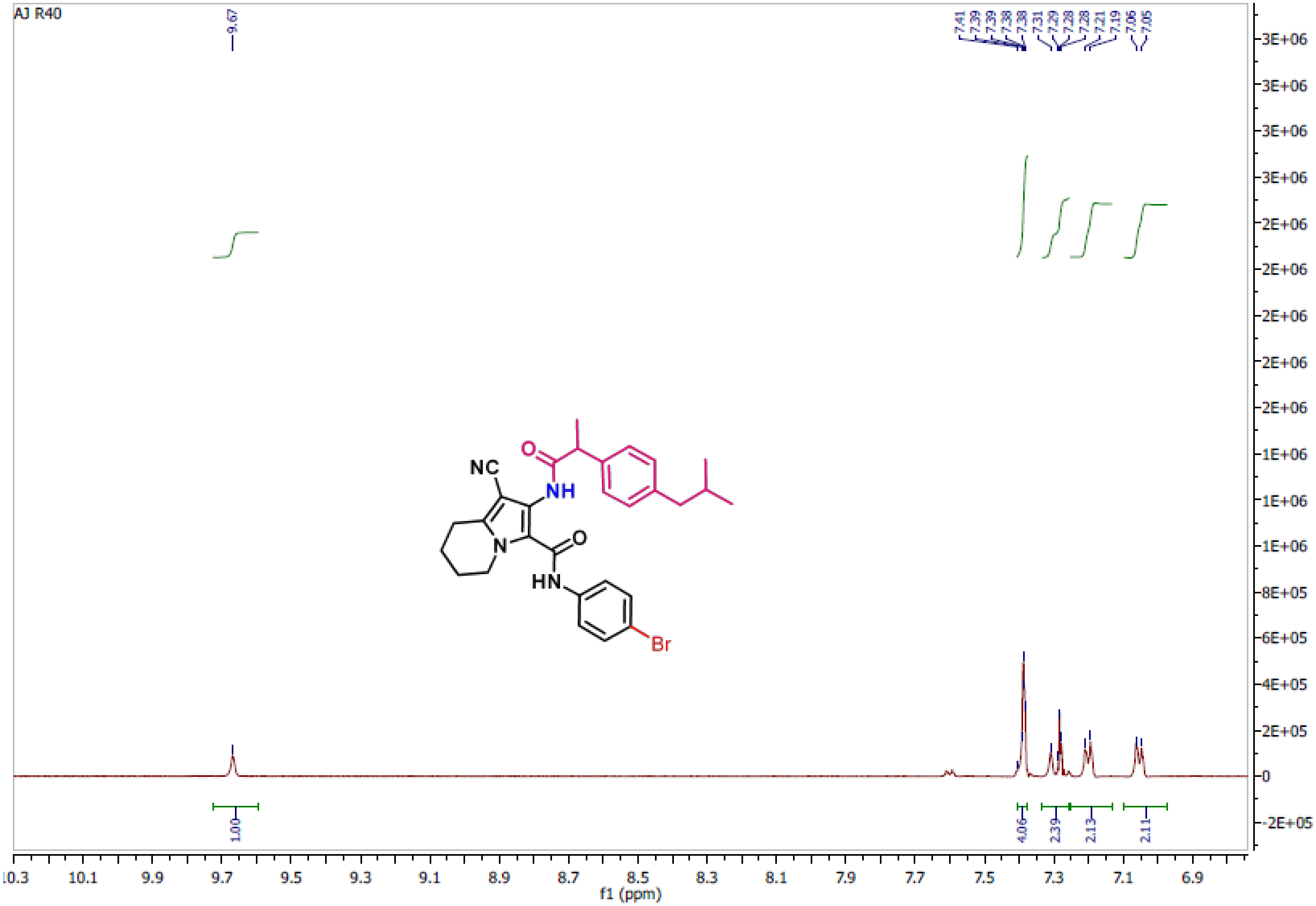

Fig. S73. ^{13}C-NMR ($CDCl_3$, 125 MHz, δ ppm) spectrum of compound **8i**

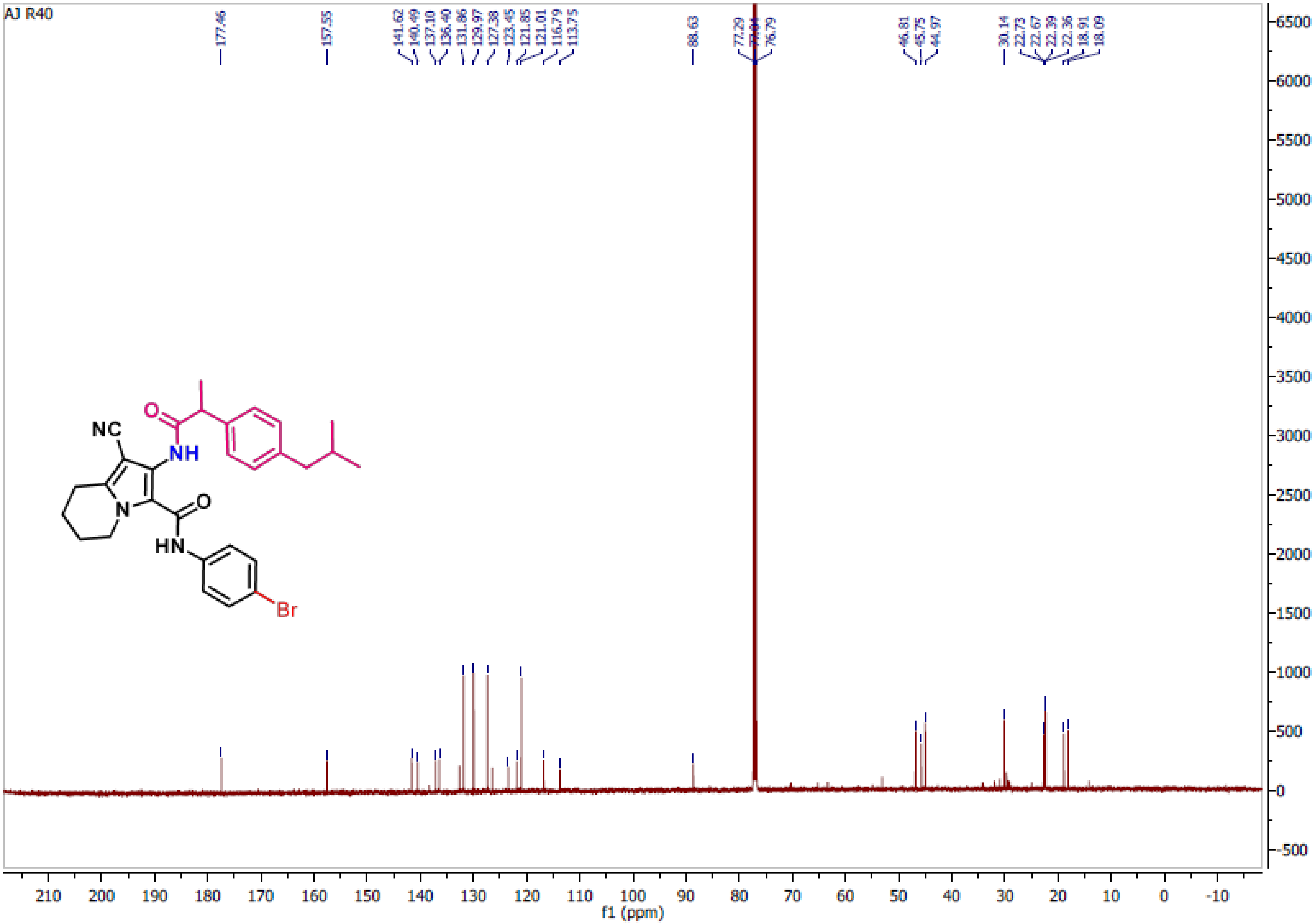

Fig. S74. ^{13}C-NMR ($CDCl_3$, 125 MHz, δ ppm) spectrum of compound **8i** **(zoom on aliphatic Cs)**

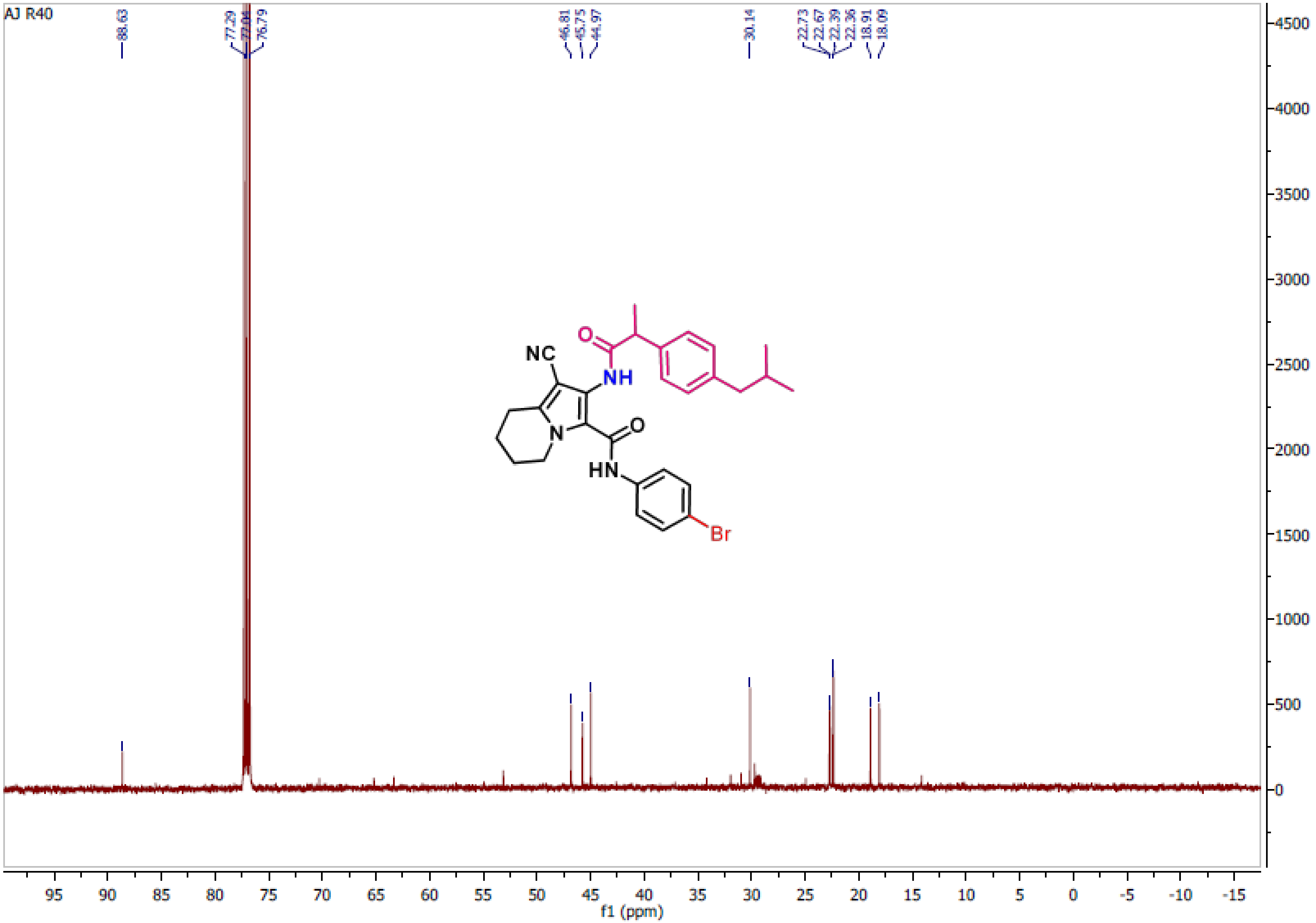

Fig. S75. ^{13}C-NMR ($CDCl_3$, 125 MHz, δ ppm) spectrum of compound **8i** **(zoom on aromatic Cs)**

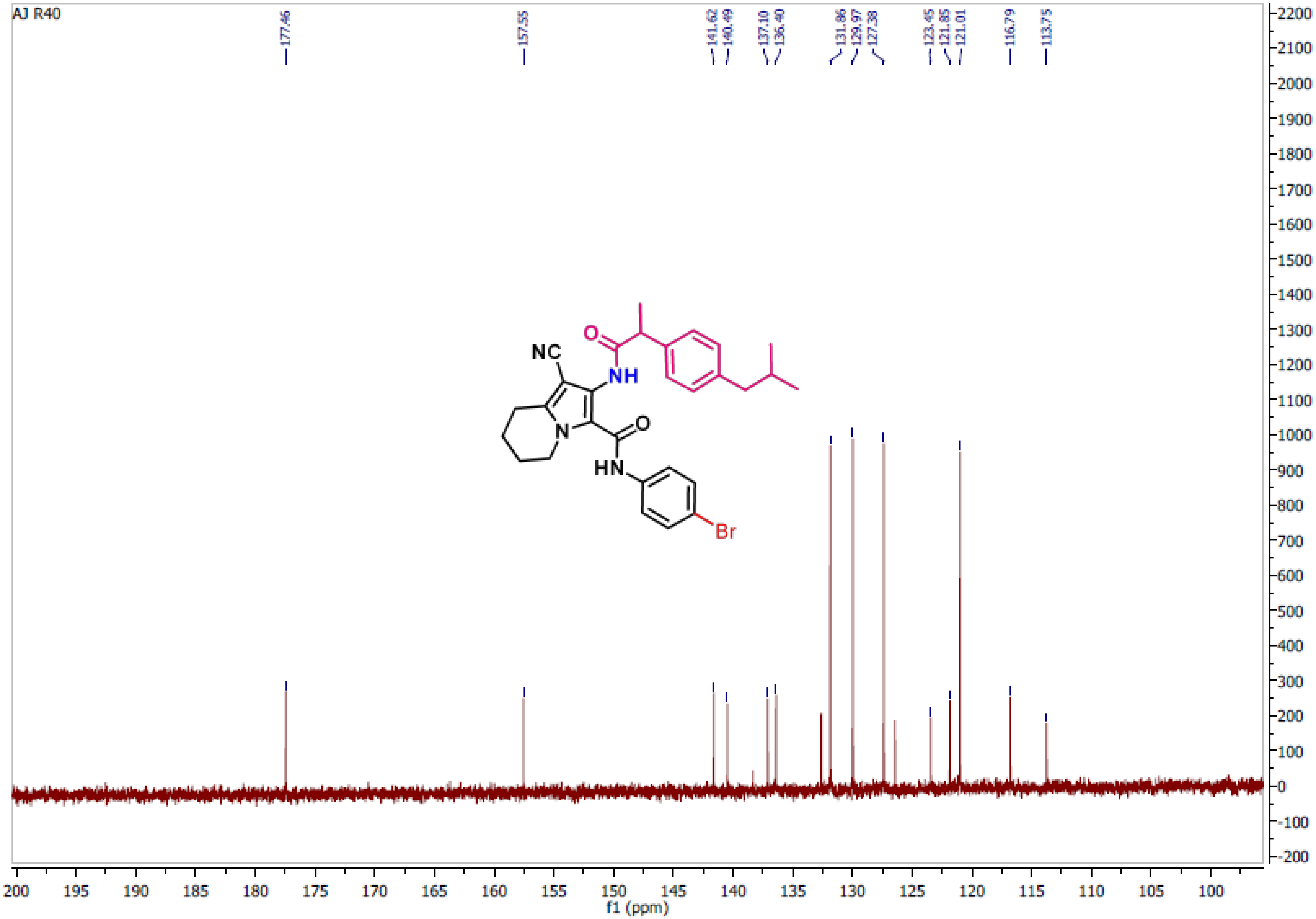

Fig. S76. DEPT C[135] ($CDCl_3$, 125 MHz, δ ppm) of compound **8i**

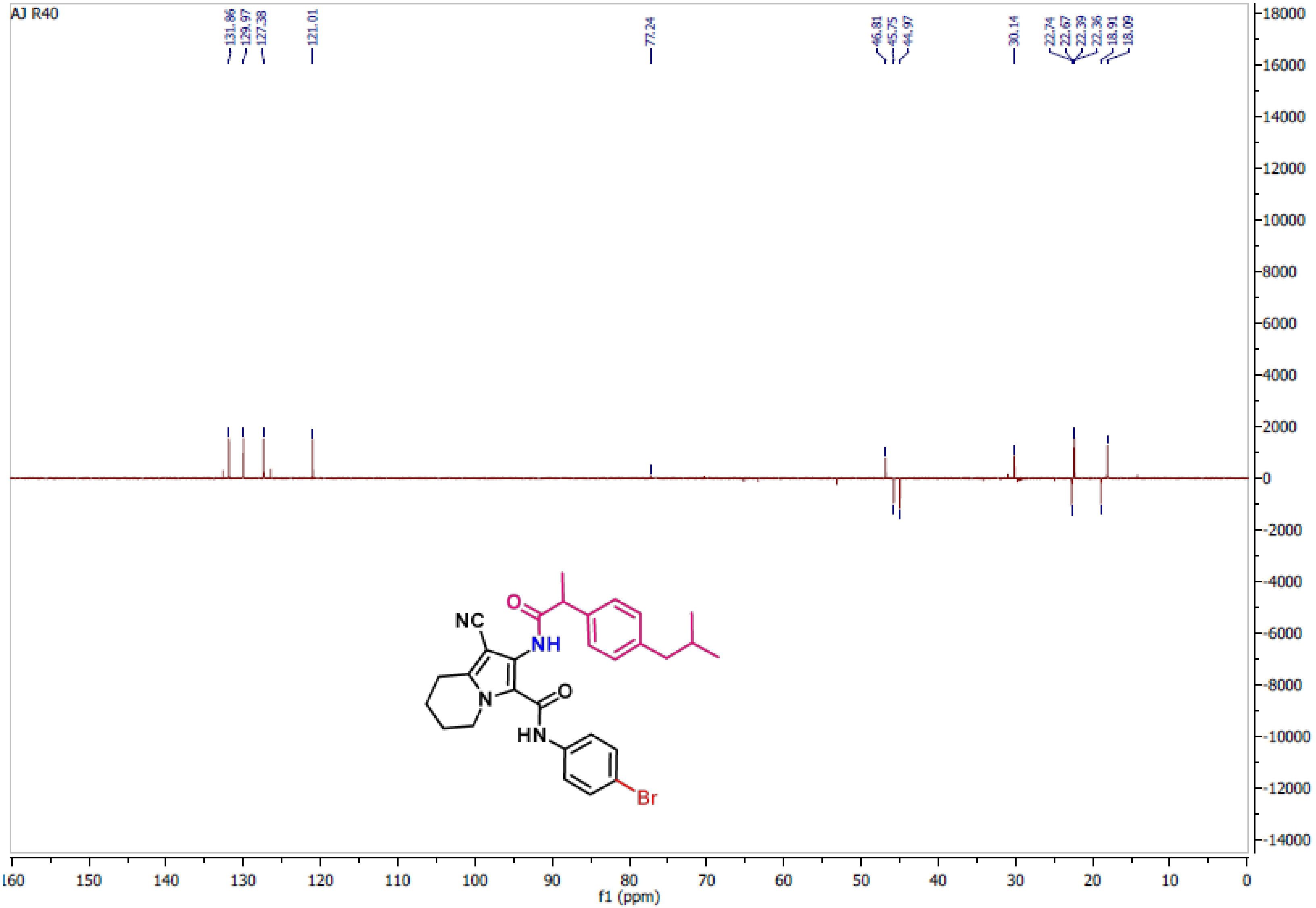

Fig. S77. DEPT C[135] ($CDCl_3$, 125 MHz, *δ* ppm) of compound **8i** **(zoom on aliphatic Cs)**

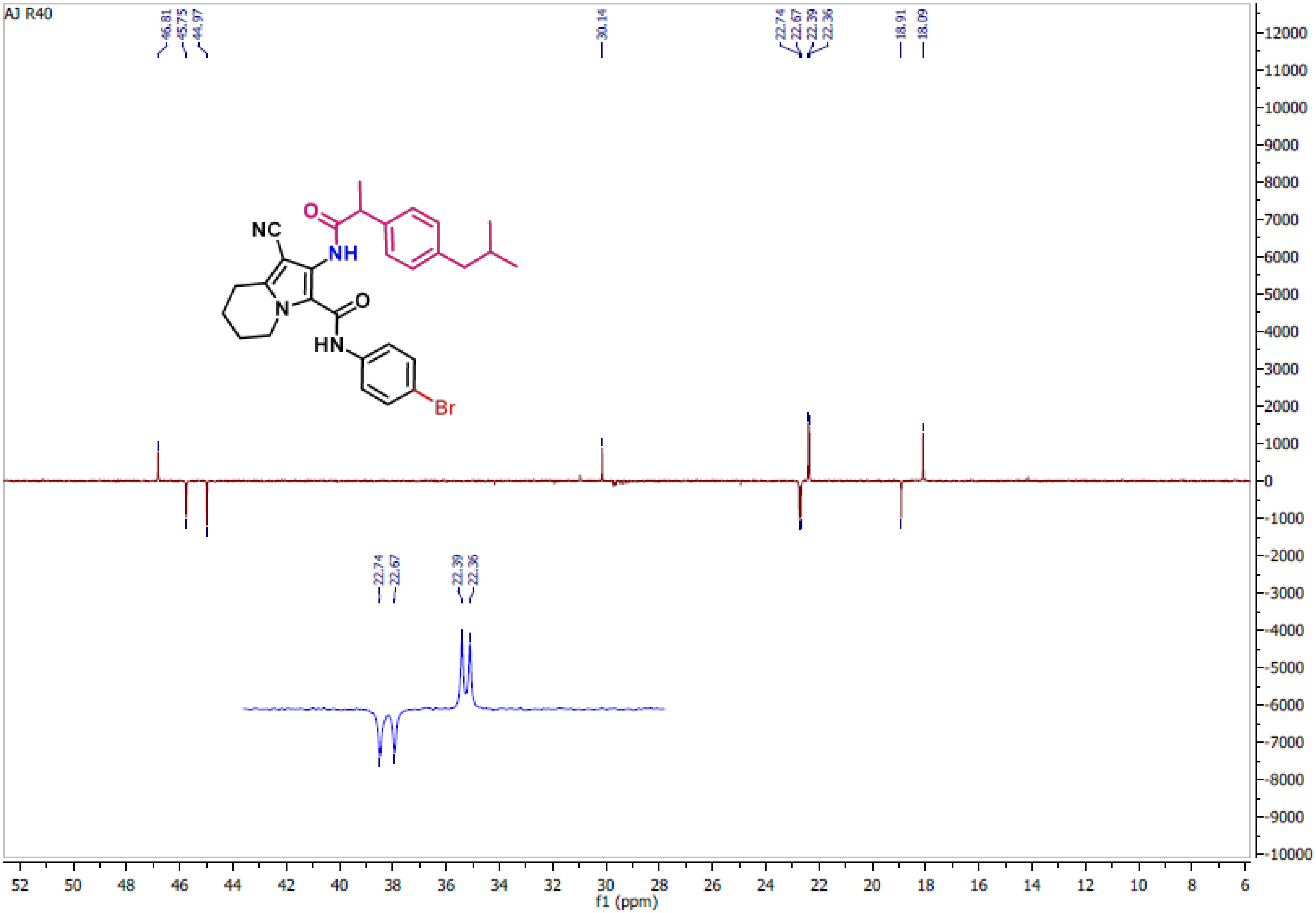

Supplementary materials

Fig. S78. DEPT C135 ($CDCl_3$, 125 MHz, δ ppm) of compound **8i** **(zoom on aromatic Cs)**

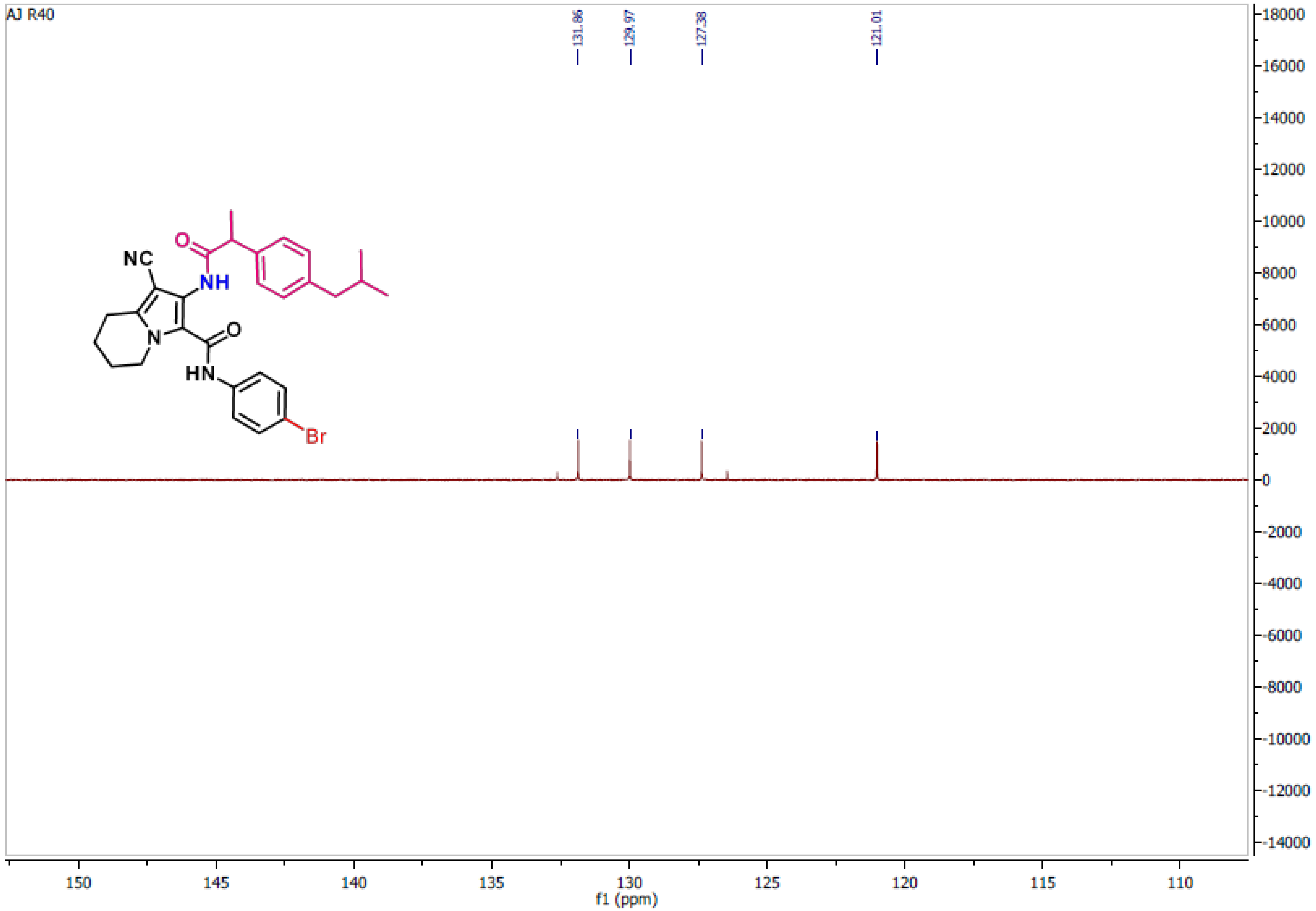

Docking Study

Validation of the docking procedures

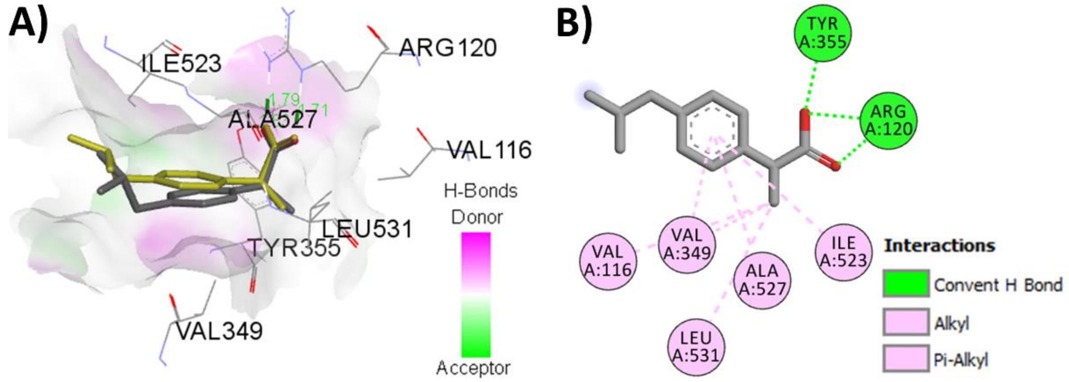

Fig. S79. Binding modes/interactions of re-docked ibuprofen (shown as stick, colored by element) into the active site of COX-1 (pdb code: 1EQG): A) 3D binding mode of re-docked ibuprofen superposed with the co-crystallized ibuprofen (yellow stacks); B) 2D binding mode of the co-crystallized ibuprofen into COX-1 showing different types of binding interactions, hydrogen atoms were omitted for clarity.

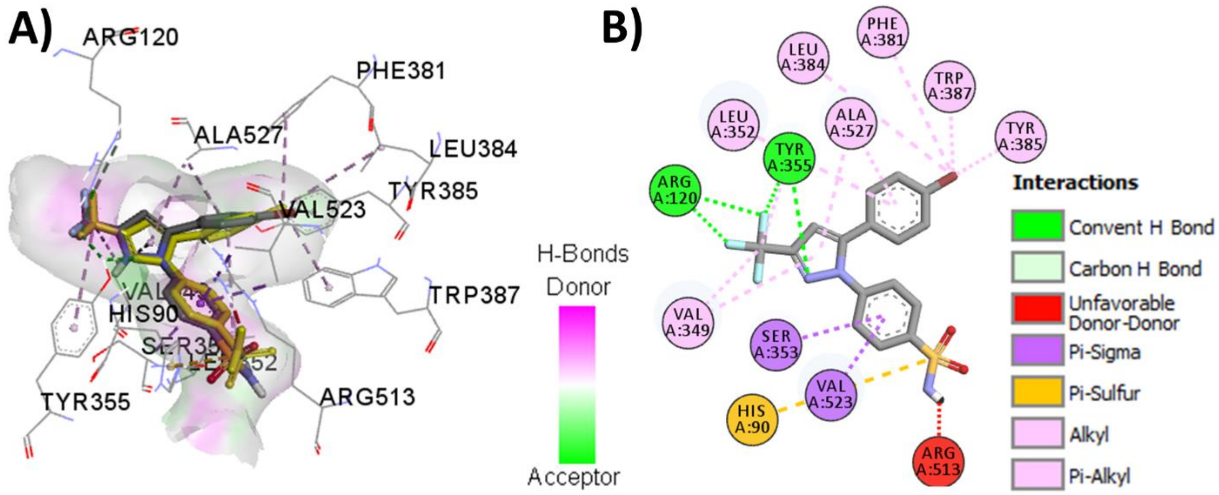

Fig. S80. Binding modes/interactions of the re-docked SC-558 (shown as stick, colored by element) into the active site of COX-2 (pdb code: 1CX2): A) 3D binding mode of the re-docked SC-558 superposed with the co-crystallized SC588 (yellow stacks); B) 2D binding mode of co-crystallized SC588 into COX-2 showing different types of binding interactions, hydrogen atoms were omitted for clarity.

Results of the docking study of compounds 5a,c

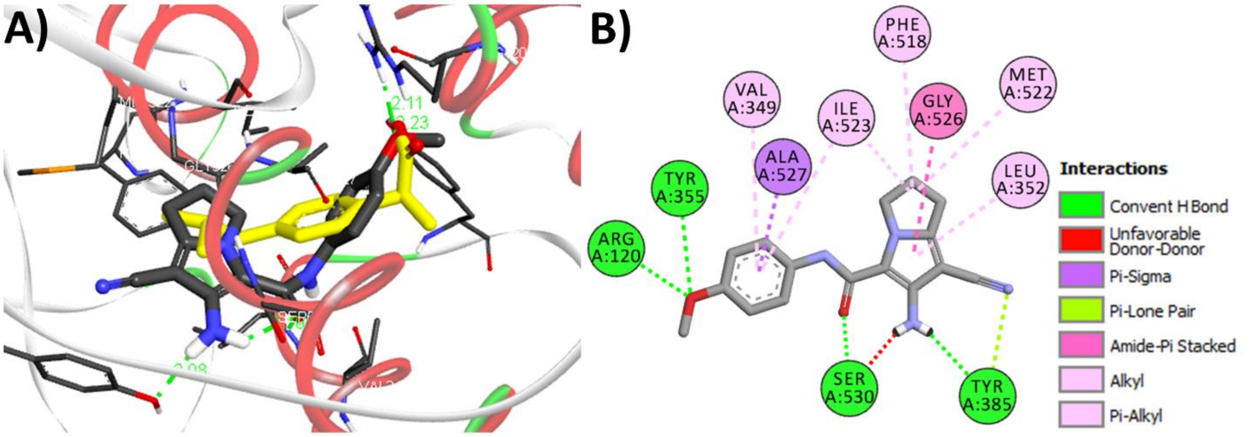

Fig. S81. Binding modes/interactions of compound **5a** (shown as stick, colored by element) into the active site of COX-1 (pdb code: 1EQG): A) 3D binding mode of **5a** superposed with the co-crystallized ibuprofen (yellow stacks); B) 2D binding mode of **5a** into COX-1 showing different types of binding interactions, hydrogen atoms were omitted for clarity.

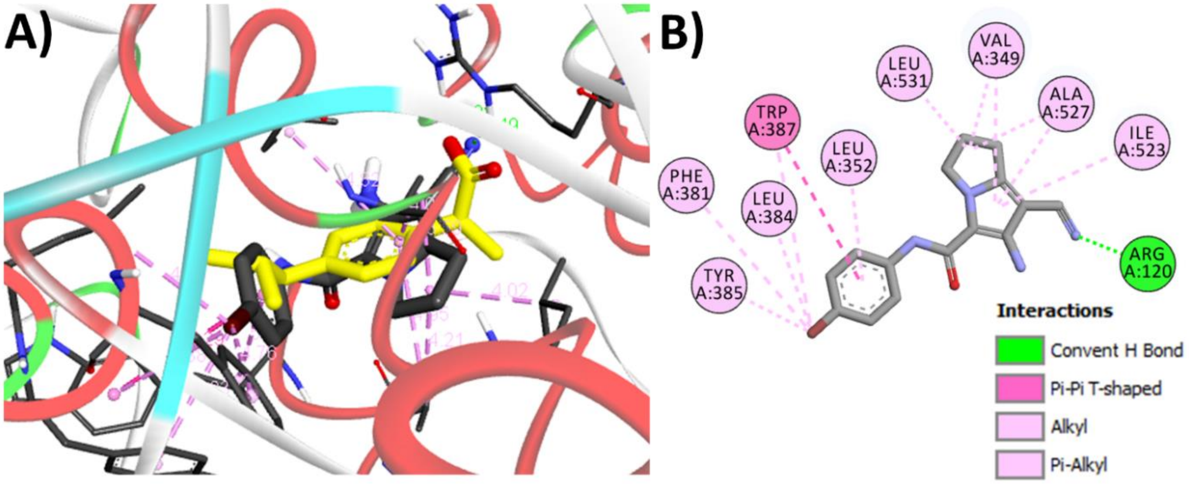

Fig. S82. Binding modes/interactions of compound **5c** (shown as stick, colored by element) into the active site of COX-1 (pdb code: 1EQG): A) 3D binding mode of **5c** superposed with the co-crystallized ibuprofen (yellow stacks); B) 2D binding mode of **5c** into COX-1 showing different types of binding interactions, hydrogen atoms were omitted for clarity.

Table S1: Molecular Properties and Drug-likeness of compound 8a

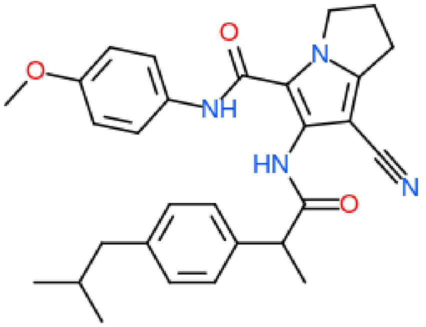

**Molecular formula:** C29 H32 N4 O3<br>**Molecular weight:** 484.25<br>**Number of HBA:** 4<br>**Number of HBD:** 2<br>**MolLogP :** 5.69 (> 5)<br>**MolLogS :** -5.40 (in Log(moles/L)) 1.95 (in mg/L)<br>**MolPSA :** 70.29 A^2<br>**MolVol :** 519.84 A^3<br>**pKa of most Basic/Acidic group :** -0.14 / 10.20<br>**BBB Score :** 3.12 The Blood-Brain Barrier (BBB) Score: 6-High,0-Low *(DOI: 10.1021/acs.jmedchem.9b01220)*<br>**Number of stereo centers:** 1

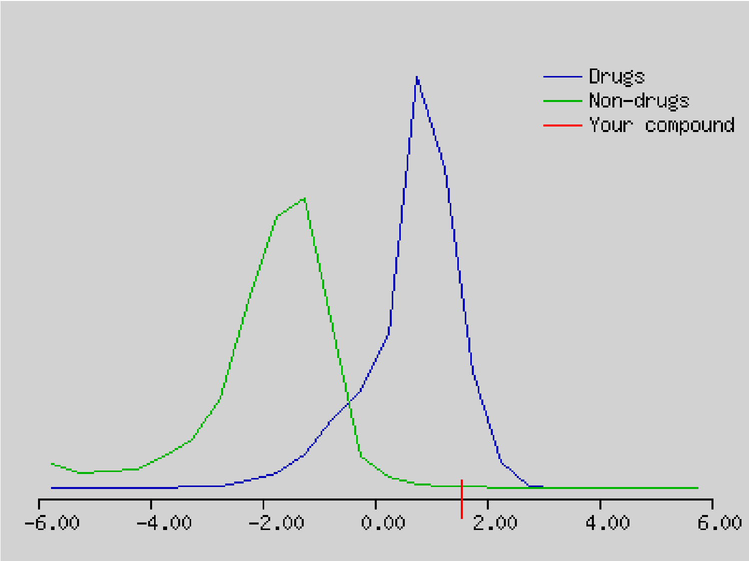

Drug-likeness model score: 1.54

Table S2: **Molecular Properties and Drug-likeness of compound 8b**

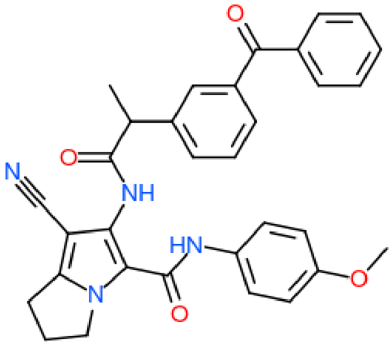

Molecular formula: C32 H28 N4 O4
Molecular weight: 532.21 (> 500)
Number of HBA: 5
Number of HBD: 2
MolLogP : 5.03 (> 5)
MolLogS : -4.95 (in Log(moles/L)) 6.03 (in mg/L)
MolPSA : 83.76 A^2
MolVol : 550.92 A^3
pKa of most Basic/Acidic group : -0.14 / 10.20
BBB Score : 1.86 The Blood-Brain Barrier (BBB) Score: 6-High,0-Low *(DOI: 10.1021/acs.jmedchem.9b01220)*
Number of stereo centers: 1

Drug-likeness model score: 1.06

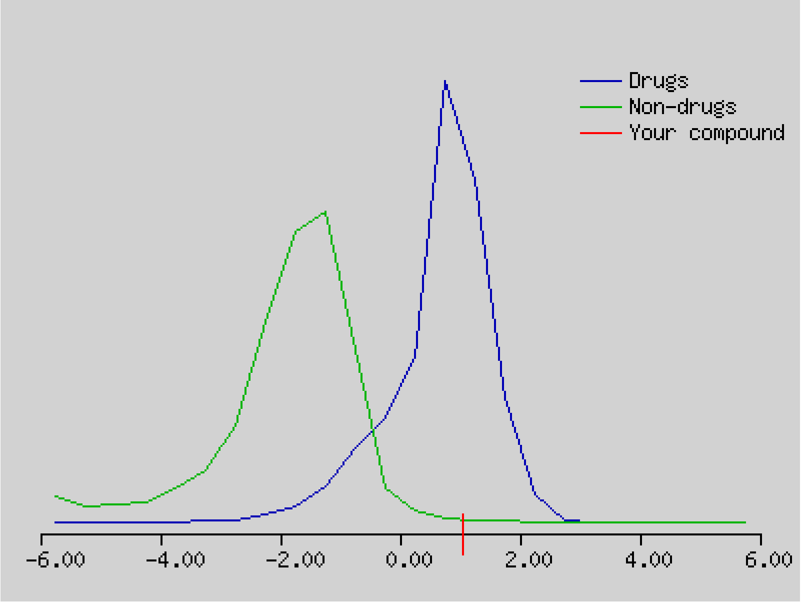

Table S3: Molecular Properties and Drug-likeness of compound 8c

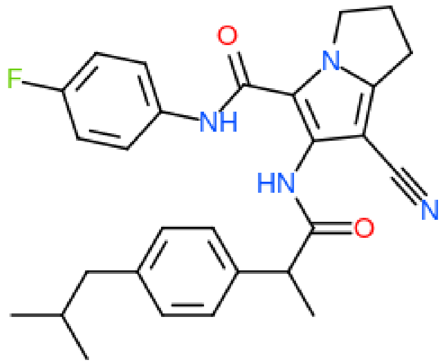

Molecular formula: C28 H29 F N4 O2
Molecular weight: 472.23
Number of HBA: 3
Number of HBD: 2
MolLogP : 5.80 (> 5)
MolLogS : -5.47 (in Log(moles/L)) 1.61 (in mg/L)
MolPSA : 62.75 A^2
MolVol : 493.90 A^3
pKa of most Basic/Acidic group : -2.29 / 9.50
BBB Score : 3.49 The Blood-Brain Barrier (BBB) Score: 6-High,0-Low *(DOI: 10.1021/acs.jmedchem.9b01220)*
Number of stereo centers: 1

Drug-likeness model score: 1.76

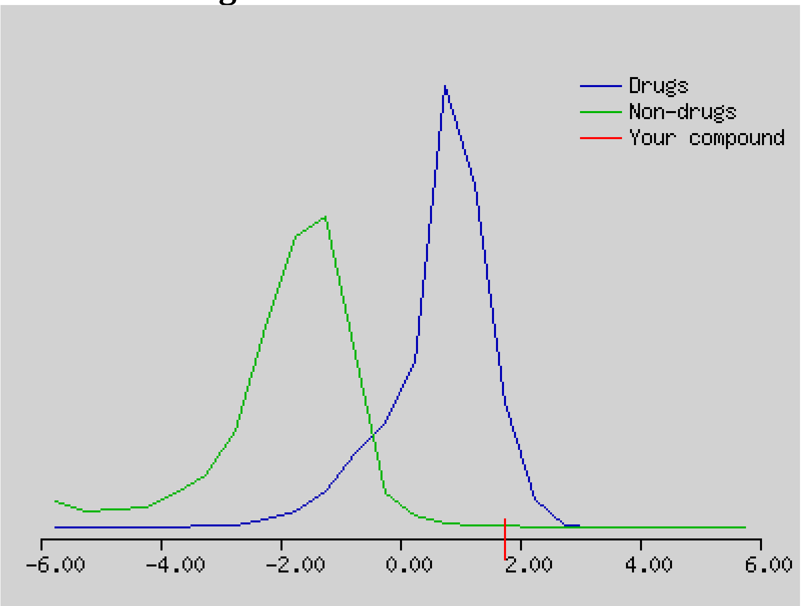

Table S4: Molecular Properties and Drug-likeness of compound 8d

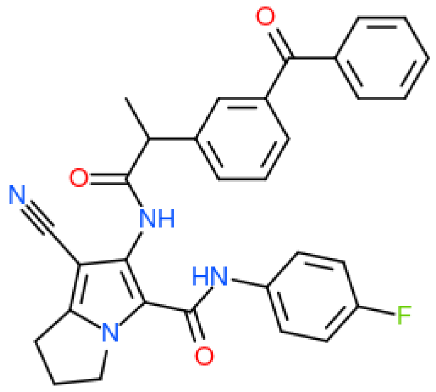

Molecular formula: C31 H25 F N4 O3
Molecular weight: 520.19 (> 500)
Number of HBA: 4
Number of HBD: 2
MolLogP : 5.14 (> 5)
MolLogS : -5.05 (in Log(moles/L)) 4.61 (in mg/L)
MolPSA : 76.21 A^2
MolVol : 524.99 A^3
pKa of most Basic/Acidic group : -2.29 / 9.50
BBB Score : 2.23 The Blood-Brain Barrier (BBB) Score: 6-High,0-Low *(DOI: 10.1021/acs.jmedchem.9b01220)*
Number of stereo centers: 1

Drug-likeness model score: 1.29

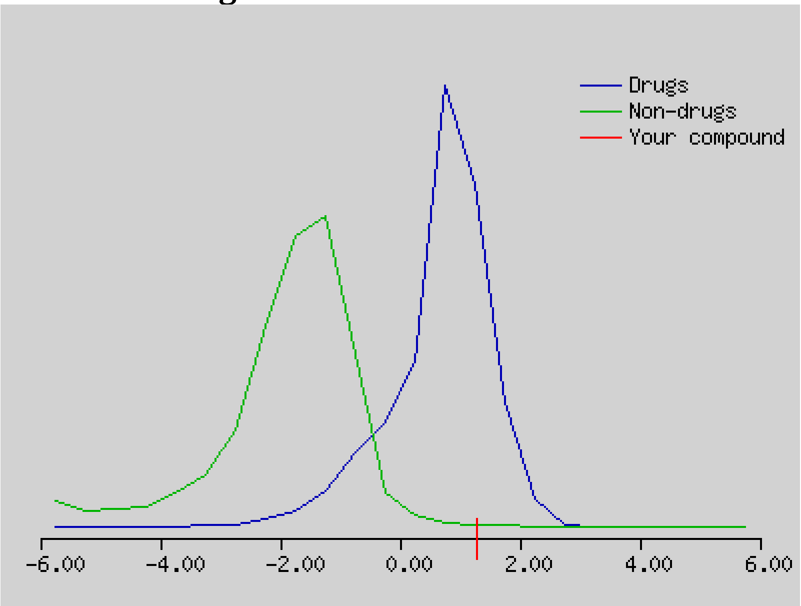

Table S5: Molecular Properties and Drug-likeness of compound **8e**

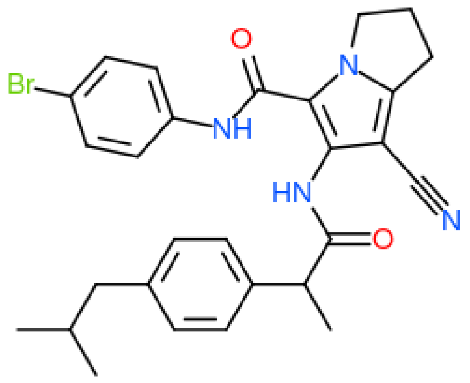

Molecular formula: C28 H29 Br N4 O2
Molecular weight: 532.15 (> 500)
Number of HBA: 3
Number of HBD: 2
MolLogP : 6.57 (> 5)
MolLogS : -5.78 (in Log(moles/L)) 0.89 (in mg/L)
MolPSA : 62.75 A^2
MolVol : 509.84 A^3
pKa of most Basic/Acidic group : -2.29 / 9.50
BBB Score : 3.59 The Blood-Brain Barrier (BBB) Score: 6-High,0-Low *(DOI: 10.1021/acs.jmedchem.9b01220)*
Number of stereo centers: 1

Drug-likeness model score: 1.59

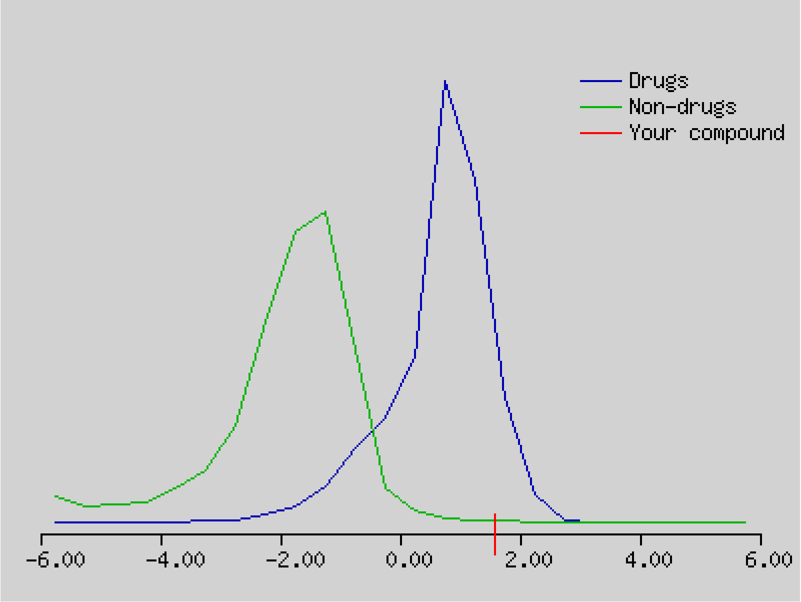

Table S6: Molecular Properties and Drug-likeness of compound 8f

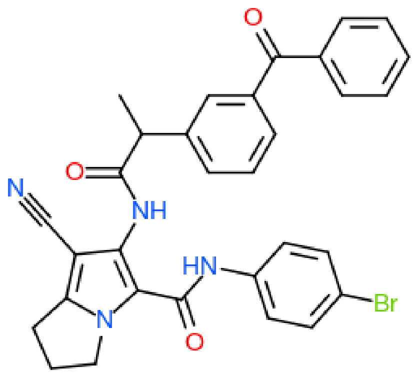

Molecular formula: C31 H25 Br N4 O3
Molecular weight: 580.11 (> 500)
Number of HBA: 4
Number of HBD: 2
MolLogP : 5.91 (> 5)
MolLogS : -5.75 (in Log(moles/L)) 1.03 (in mg/L)
MolPSA : 76.21 A^2
MolVol : 540.93 A^3
pKa of most Basic/Acidic group : -2.29 / 9.50
BBB Score : 2.36 The Blood-Brain Barrier (BBB) Score: 6-High,0-Low *(DOI: 10.1021/acs.jmedchem.9b01220)*
Number of stereo centers: 1

Drug-likeness model score: 1.13

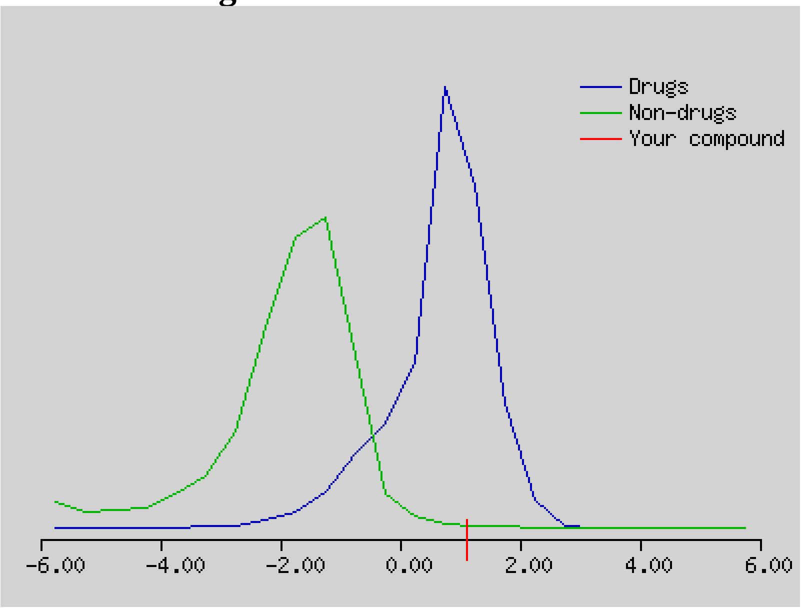

Table S7: **Molecular Properties and Drug-likeness of compound 8g**

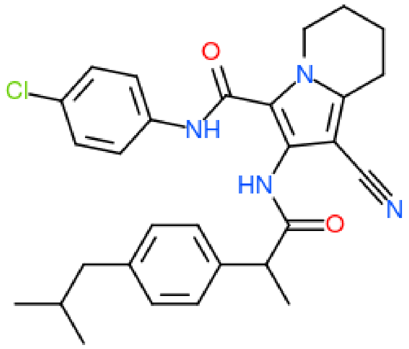

Molecular formula: C29 H31 Cl N4 O2
Molecular weight: 502.21 (> 500)
Number of HBA: 3
Number of HBD: 2
MolLogP : 6.71 (> 5)
MolLogS : -6.64 (in Log(moles/L)) 0.11 (in mg/L)
MolPSA : 62.38 A^2
MolVol : 522.19 A^3
pKa of most Basic/Acidic group : -2.19 / 9.50
BBB Score : 3.50 The Blood-Brain Barrier (BBB) Score: 6-High,0-Low *(DOI: 10.1021/acs.jmedchem.9b01220)*
Number of stereo centers: 1

Drug-likeness model score: 2.03

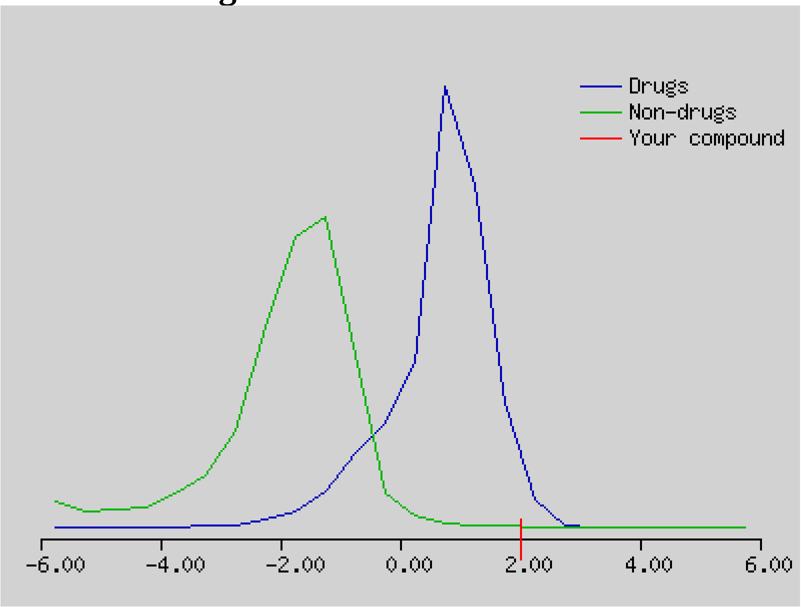

Table S8: **Molecular Properties and Drug-likeness of compound 8h**

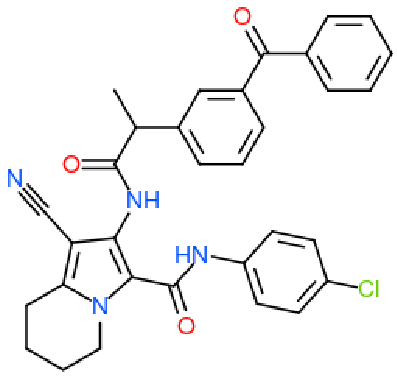

Molecular formula: C32 H27 Cl N4 O3
Molecular weight: 550.18 (> 500)
Number of HBA: 4
Number of HBD: 2
MolLogP : 6.05 (> 5)
MolLogS : -6.24 (in Log(moles/L)) 0.32 (in mg/L)
MolPSA : 75.85 A^2
MolVol : 553.27 A^3
pKa of most Basic/Acidic group : -2.19 / 9.50
BBB Score : 2.26 The Blood-Brain Barrier (BBB) Score: 6-High,0-Low *(DOI: 10.1021/acs.jmedchem.9b01220)*
Number of stereo centers: 1

Drug-likeness model score: 1.57

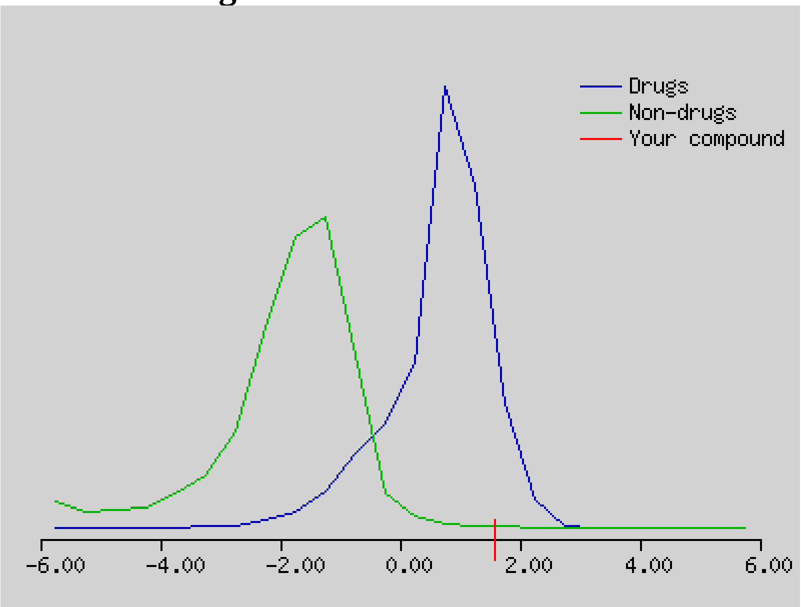

Table S9: Molecular Properties and Drug-likeness of compound 8i

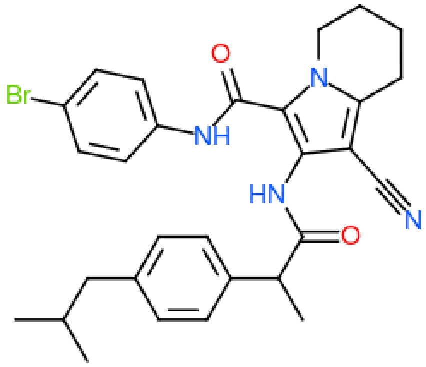

Molecular formula: C29 H31 Br N4 O2
Molecular weight: 546.16 (> 500)
Number of HBA: 3
Number of HBD: 2
MolLogP : 6.95 (> 5)
MolLogS : -5.83 (in Log(moles/L)) 0.80 (in mg/L)
MolPSA : 62.38 A^2
MolVol : 526.85 A^3
pKa of most Basic/Acidic group : -2.19 / 9.50
BBB Score : 3.56 The Blood-Brain Barrier (BBB) Score: 6-High,0-Low *(DOI: 10.1021/acs.jmedchem.9b01220)*
Number of stereo centers: 1

Drug-likeness model score: 1.73

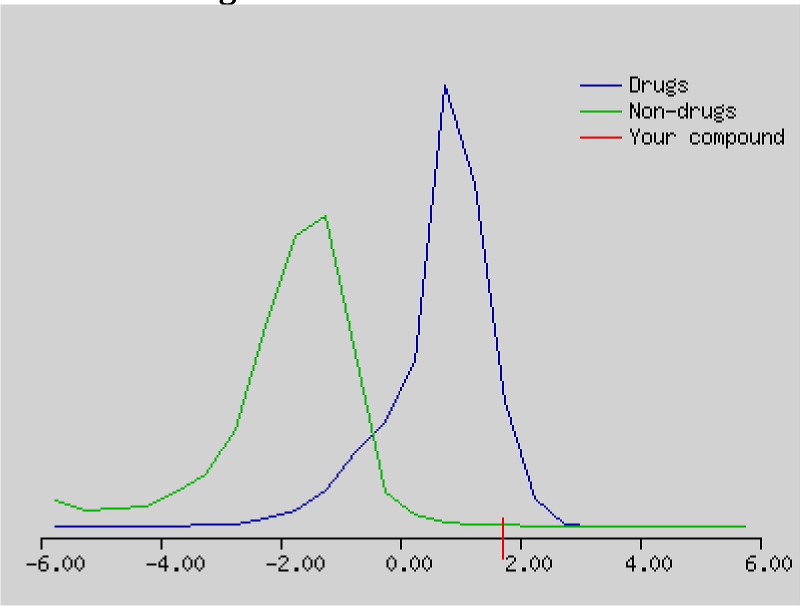

Table S10: ADME-related properties of compound **8a**

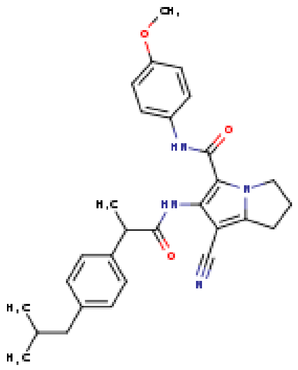

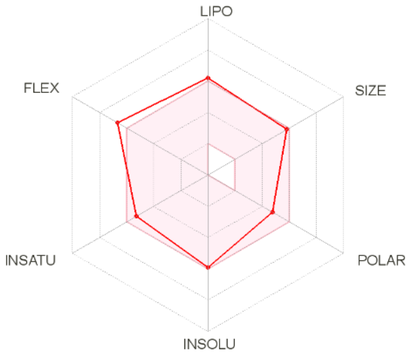

SMILES N#Cc1c2CCCn2c(c1NC(=O)C(c1ccc(cc1)CC(C)C)C)C(=O)Nc1ccc(cc1)OC

Physicochemical Properties	
Formula	C29H32N4O3
Molecular weight	484.59 g/mol
Num. heavy atoms	36
Num. arom. heavy atoms	17
Fraction Csp3	0.34
Num. rotatable bonds	10
Num. H-bond acceptors	4
Num. H-bond donors	2
Molar Refractivity	141.77
TPSA	96.15 Å²
Lipophilicity	
Log $P_{o/w}$ (iLOGP)	3.96
Log $P_{o/w}$ (XLOGP3)	5.37
Log $P_{o/w}$ (WLOGP)	5.13
Log $P_{o/w}$ (MLOGP)	2.64
Log $P_{o/w}$ (SILICOS-IT)	5.25
Consensus Log $P_{o/w}$	4.47
Water Solubility	

Log *S* (ESOL)	-5.92
Solubility	5.87e-04 mg/ml ; 1.21e-06 mol/l
Class	Moderately soluble
Log *S* (Ali)	-7.14
Solubility	3.49e-05 mg/ml ; 7.20e-08 mol/l
Class	Poorly soluble
Log *S* (SILICOS-IT)	-8.36
Solubility	2.10e-06 mg/ml ; 4.34e-09 mol/l
Class	Poorly soluble
Pharmacokinetics	
GI absorption	High
BBB permeant	No
P-gp substrate	Yes
CYP1A2 inhibitor	No
CYP2C19 inhibitor	Yes
CYP2C9 inhibitor	Yes
CYP2D6 inhibitor	Yes
CYP3A4 inhibitor	Yes
Log K_p (skin permeation)	-5.44 cm/s
Druglikeness	
Lipinski	Yes; 0 violation
Ghose	No; 2 violations: MW>480, MR>130
Veber	Yes
Egan	Yes
Muegge	No; 1 violation: XLOGP3>5
Bioavailability Score	0.55
Medicinal Chemistry	
PAINS	0 alert
Brenk	0 alert
Leadlikeness	No; 3 violations: MW>350, Rotors>7, XLOGP3>3.5

Synthetic accessibility	4.46

Table S11: ADME-related properties of compound 8b

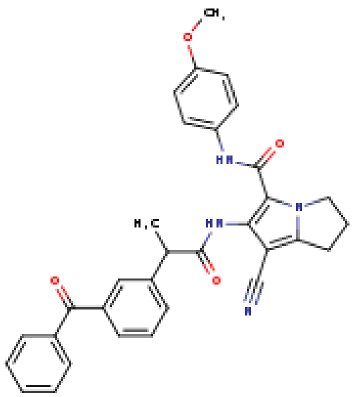

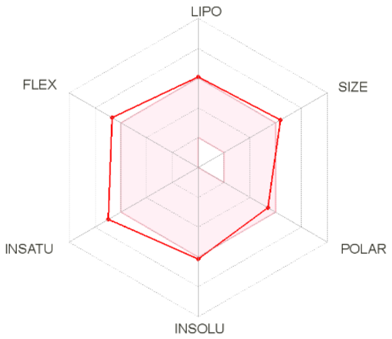

SMILES N#Cc1c2CCCn2c(c1NC(=O)C(c1cccc(c1)C(=O)c1ccccc1)C)C(=O)Nc1ccc(cc1)OC

Physicochemical Properties	
Formula	C32H28N4O4
Molecular weight	532.59 g/mol
Num. heavy atoms	40
Num. arom. heavy atoms	23
Fraction Csp3	0.19
Num. rotatable bonds	10
Num. H-bond acceptors	5
Num. H-bond donors	2
Molar Refractivity	152.26
TPSA	113.22 Å²
**Lipophilicity**	
Log $P_{o/w}$ (iLOGP)	3.11
Log $P_{o/w}$ (XLOGP3)	5.12
Log $P_{o/w}$ (WLOGP)	5.16
Log $P_{o/w}$ (MLOGP)	2.14

Log $P_{o/w}$ (SILICOS-IT)	5.21
Consensus Log $P_{o/w}$	4.15
Water Solubility	
Log S (ESOL)	-6.13
Solubility	3.92e-04 mg/ml ; 7.36e-07 mol/l
Class	Poorly soluble
Log S (Ali)	-7.24
Solubility	3.05e-05 mg/ml ; 5.73e-08 mol/l
Class	Poorly soluble
Log S (SILICOS-IT)	-9.55
Solubility	1.51e-07 mg/ml ; 2.84e-10 mol/l
Class	Poorly soluble
Pharmacokinetics	
GI absorption	Low
BBB permeant	No
P-gp substrate	No
CYP1A2 inhibitor	No
CYP2C19 inhibitor	Yes
CYP2C9 inhibitor	Yes
CYP2D6 inhibitor	No
CYP3A4 inhibitor	Yes
Log K_p (skin permeation)	-5.91 cm/s
Druglikeness	
Lipinski	Yes; 1 violation: MW>500
Ghose	No; 2 violations: MW>480, MR>130
Veber	Yes
Egan	Yes
Muegge	No; 1 violation: XLOGP3>5
Bioavailability Score	0.55
Medicinal Chemistry	
PAINS	0 alert

Brenk	0 alert
Leadlikeness	No; 3 violations: MW>350, Rotors>7, XLOGP3>3.5
Synthetic accessibility	4.51

Table S12: ADME-related properties of compound 8c

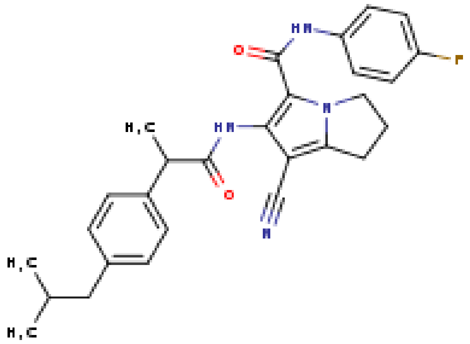

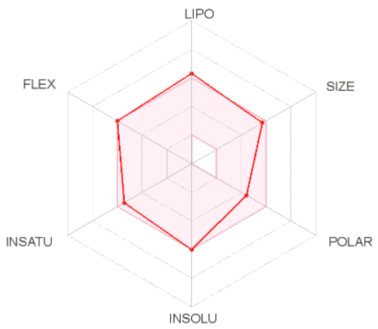

SMILES N#Cc1c2CCCn2c(c1NC(=O)C(c1ccc(cc1)CC(C)C)C)C(=O)Nc1ccc(cc1)F

Physicochemical Properties	
Formula	C28H29FN4O2
Molecular weight	472.55 g/mol
Num. heavy atoms	35
Num. arom. heavy atoms	17
Fraction Csp3	0.32
Num. rotatable bonds	9
Num. H-bond acceptors	4
Num. H-bond donors	2
Molar Refractivity	135.23
TPSA	86.92 Å²
Lipophilicity	
Log $P_{o/w}$ (iLOGP)	3.93
Log $P_{o/w}$ (XLOGP3)	5.50
Log $P_{o/w}$ (WLOGP)	5.68
Log $P_{o/w}$ (MLOGP)	3.34

Log $P_{o/w}$ (SILICOS-IT)	5.59
Consensus Log $P_{o/w}$	4.81
Water Solubility	
Log *S* (ESOL)	-6.00
Solubility	4.72e-04 mg/ml ; 9.99e-07 mol/l
Class	Poorly soluble
Log *S* (Ali)	-7.08
Solubility	3.90e-05 mg/ml ; 8.24e-08 mol/l
Class	Poorly soluble
Log *S* (SILICOS-IT)	-8.53
Solubility	1.40e-06 mg/ml ; 2.96e-09 mol/l
Class	Poorly soluble
Pharmacokinetics	
GI absorption	High
BBB permeant	No
P-gp substrate	Yes
CYP1A2 inhibitor	No
CYP2C19 inhibitor	Yes
CYP2C9 inhibitor	Yes
CYP2D6 inhibitor	Yes
CYP3A4 inhibitor	Yes
Log K_p (skin permeation)	-5.28 cm/s
Druglikeness	
Lipinski	Yes; 0 violation
Ghose	No; 2 violations: WLOGP>5.6, MR>130
Veber	Yes
Egan	Yes
Muegge	No; 1 violation: XLOGP3>5
Bioavailability Score	0.55

Medicinal Chemistry	
PAINS	0 alert
Brenk	0 alert
Leadlikeness	No; 3 violations: MW>350, Rotors>7, XLOGP3>3.5
Synthetic accessibility	4.34

Table S13: ADME-related properties of compound **8d**

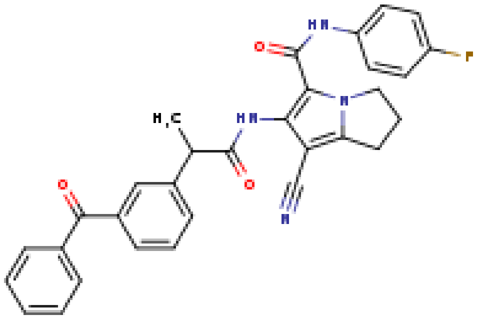

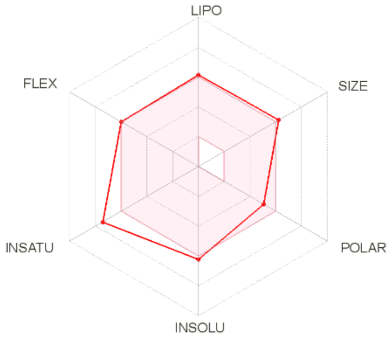

SMILES N#Cc1c2CCCn2c(c1NC(=O)C(c1cccc(c1)C(=O)c1ccccc1)C)C(=O)Nc1ccc(cc1)F

Physicochemical Properties	
Formula	C31H25FN4O3
Molecular weight	520.55 g/mol
Num. heavy atoms	39
Num. arom. heavy atoms	23
Fraction Csp3	0.16
Num. rotatable bonds	9
Num. H-bond acceptors	5
Num. H-bond donors	2
Molar Refractivity	145.72
TPSA	103.99 Å²
**Lipophilicity**	
Log $P_{o/w}$ (iLOGP)	3.11
Log $P_{o/w}$ (XLOGP3)	5.25

Log $P_{o/w}$ (WLOGP)	5.71
Log $P_{o/w}$ (MLOGP)	2.84
Log $P_{o/w}$ (SILICOS-IT)	5.55
Consensus Log $P_{o/w}$	4.49
Water Solubility	
Log S (ESOL)	-6.22
Solubility	3.16e-04 mg/ml ; 6.06e-07 mol/l
Class	Poorly soluble
Log S (Ali)	-7.18
Solubility	3.42e-05 mg/ml ; 6.56e-08 mol/l
Class	Poorly soluble
Log S (SILICOS-IT)	-9.71
Solubility	1.01e-07 mg/ml ; 1.94e-10 mol/l
Class	Poorly soluble
Pharmacokinetics	
GI absorption	Low
BBB permeant	No
P-gp substrate	No
CYP1A2 inhibitor	No
CYP2C19 inhibitor	Yes
CYP2C9 inhibitor	Yes
CYP2D6 inhibitor	No
CYP3A4 inhibitor	Yes
Log K_p (skin permeation)	-5.75 cm/s
Druglikeness	
Lipinski	Yes; 1 violation: MW>500
Ghose	No; 3 violations: MW>480, WLOGP>5.6, MR>130
Veber	Yes
Egan	Yes
Muegge	No; 1 violation: XLOGP3>5
Bioavailability Score	0.55

Medicinal Chemistry	
PAINS	0 alert
Brenk	0 alert
Leadlikeness	No; 3 violations: MW>350, Rotors>7, XLOGP3>3.5
Synthetic accessibility	4.39

Table S14: ADME-related properties of compound 8e

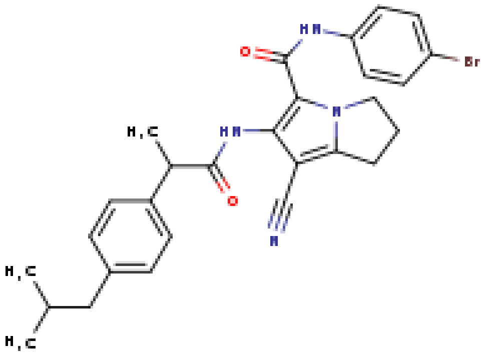

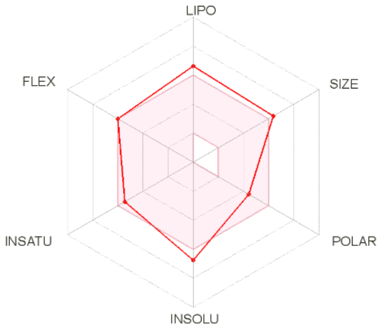

SMILES N#Cc1c2CCCn2c(c1NC(=O)C(c1ccc(cc1)CC(C)C)C)C(=O)Nc1ccc(cc1)Br

Physicochemical Properties	
Formula	C28H29BrN4O2
Molecular weight	533.46 g/mol
Num. heavy atoms	35
Num. arom. heavy atoms	17
Fraction Csp3	0.32
Num. rotatable bonds	9
Num. H-bond acceptors	3
Num. H-bond donors	2
Molar Refractivity	142.98
TPSA	86.92 $Å^2$
**Lipophilicity**	
Log $P_{o/w}$ (iLOGP)	4.44

Log $P_{o/w}$ (XLOGP3)	6.09
Log $P_{o/w}$ (WLOGP)	5.88
Log $P_{o/w}$ (MLOGP)	3.53
Log $P_{o/w}$ (SILICOS-IT)	5.84
Consensus Log $P_{o/w}$	5.16
Water Solubility	
Log S (ESOL)	-6.75
Solubility	9.50e-05 mg/ml ; 1.78e-07 mol/l
Class	Poorly soluble
Log S (Ali)	-7.70
Solubility	1.07e-05 mg/ml ; 2.01e-08 mol/l
Class	Poorly soluble
Log S (SILICOS-IT)	-9.03
Solubility	4.97e-07 mg/ml ; 9.31e-10 mol/l
Class	Poorly soluble
Pharmacokinetics	
GI absorption	High
BBB permeant	No
P-gp substrate	Yes
CYP1A2 inhibitor	No
CYP2C19 inhibitor	Yes
CYP2C9 inhibitor	Yes
CYP2D6 inhibitor	Yes
CYP3A4 inhibitor	Yes
Log K_p (skin permeation)	-5.23 cm/s
Druglikeness	
Lipinski	Yes; 1 violation: MW>500
Ghose	No; 3 violations: MW>480, WLOGP>5.6, MR>130
Veber	Yes
Egan	Yes
Muegge	No; 1 violation: XLOGP3>5

Bioavailability Score	0.55
Medicinal Chemistry	
PAINS	0 alert
Brenk	0 alert
Leadlikeness	No; 3 violations: MW>350, Rotors>7, XLOGP3>3.5
Synthetic accessibility	4.37

Table S15: ADME-related properties of compound 8f

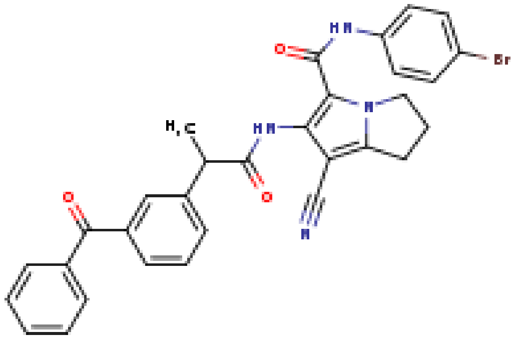

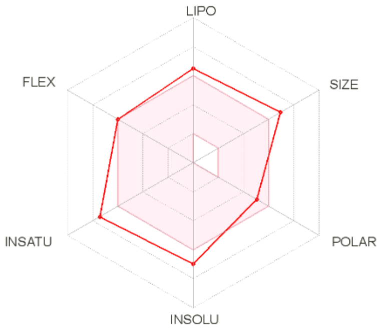

SMILES N#Cc1c2CCCn2c(c1NC(=O)C(c1cccc(c1)C(=O)c1ccccc1)C)C(=O)Nc1ccc(cc1)Br

Physicochemical Properties	
Formula	C31H25BrN4O3
Molecular weight	581.46 g/mol
Num. heavy atoms	39
Num. arom. heavy atoms	23
Fraction Csp3	0.16
Num. rotatable bonds	9
Num. H-bond acceptors	4
Num. H-bond donors	2
Molar Refractivity	153.46
TPSA	103.99 Å²
Lipophilicity	
Log $P_{o/w}$ (iLOGP)	3.54
Log $P_{o/w}$ (XLOGP3)	5.84

Log $P_{o/w}$ (WLOGP)	5.91
Log $P_{o/w}$ (MLOGP)	3.03
Log $P_{o/w}$ (SILICOS-IT)	5.81
Consensus Log $P_{o/w}$	4.82
Water Solubility	
Log *S* (ESOL)	-6.97
Solubility	6.28e-05 mg/ml ; 1.08e-07 mol/l
Class	Poorly soluble
Log *S* (Ali)	-7.80
Solubility	9.32e-06 mg/ml ; 1.60e-08 mol/l
Class	Poorly soluble
Log *S* (SILICOS-IT)	-10.21
Solubility	3.59e-08 mg/ml ; 6.18e-11 mol/l
Class	Insoluble
Pharmacokinetics	
GI absorption	Low
BBB permeant	No
P-gp substrate	No
CYP1A2 inhibitor	No
CYP2C19 inhibitor	Yes
CYP2C9 inhibitor	Yes
CYP2D6 inhibitor	No
CYP3A4 inhibitor	Yes
Log K_p (skin permeation)	-5.70 cm/s
Druglikeness	
Lipinski	Yes; 1 violation: MW>500
Ghose	No; 3 violations: MW>480, WLOGP>5.6, MR>130
Veber	Yes
Egan	No; 1 violation: WLOGP>5.88
Muegge	No; 1 violation: XLOGP3>5

Bioavailability Score	0.55
Medicinal Chemistry	
PAINS	0 alert
Brenk	0 alert
Leadlikeness	No; 3 violations: MW>350, Rotors>7, XLOGP3>3.5
Synthetic accessibility	4.42

Table S16: ADME-related properties of compound 8g

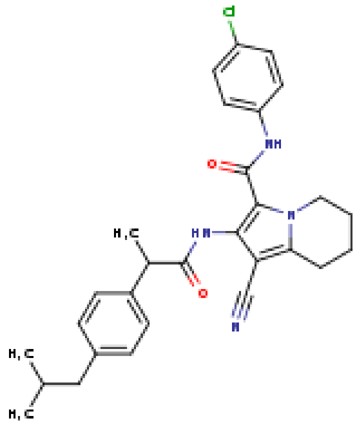

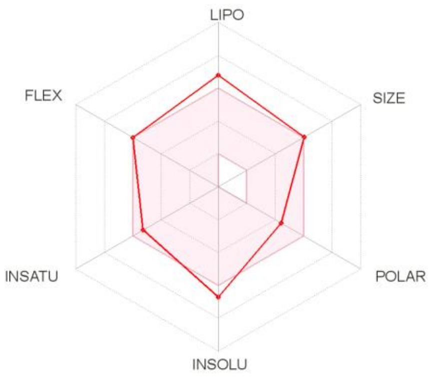

SMILES N#Cc1c(NC(=O)C(c2ccc(cc2)CC(C)C)C)c(n2c1CCCC2)C(=O)Nc1ccc(cc1)Cl

Physicochemical Properties	
Formula	C29H31ClN4O2
Molecular weight	503.04 g/mol
Num. heavy atoms	36
Num. arom. heavy atoms	17
Fraction Csp3	0.34
Num. rotatable bonds	9
Num. H-bond acceptors	3
Num. H-bond donors	2
Molar Refractivity	145.09
TPSA	86.92 Å²
Lipophilicity	

Log $P_{o/w}$ (iLOGP)	4.16
Log $P_{o/w}$ (XLOGP3)	6.38
Log $P_{o/w}$ (WLOGP)	6.16
Log $P_{o/w}$ (MLOGP)	3.63
Log $P_{o/w}$ (SILICOS-IT)	6.05
Consensus Log $P_{o/w}$	5.28
Water Solubility	
Log S (ESOL)	-6.73
Solubility	9.29e-05 mg/ml ; 1.85e-07 mol/l
Class	Poorly soluble
Log S (Ali)	-8.00
Solubility	5.06e-06 mg/ml ; 1.01e-08 mol/l
Class	Poorly soluble
Log S (SILICOS-IT)	-9.11
Solubility	3.90e-07 mg/ml ; 7.76e-10 mol/l
Class	Poorly soluble
Pharmacokinetics	
GI absorption	High
BBB permeant	No
P-gp substrate	Yes
CYP1A2 inhibitor	No
CYP2C19 inhibitor	Yes
CYP2C9 inhibitor	Yes
CYP2D6 inhibitor	Yes
CYP3A4 inhibitor	Yes
Log K_p (skin permeation)	-4.84 cm/s
Druglikeness	
Lipinski	Yes; 1 violation: MW>500
Ghose	No; 3 violations: MW>480, WLOGP>5.6, MR>130
Veber	Yes
Egan	No; 1 violation: WLOGP>5.88

Muegge	No; 1 violation: XLOGP3>5
Bioavailability Score	0.55
Medicinal Chemistry	
PAINS	0 alert
Brenk	0 alert
Leadlikeness	No; 3 violations: MW>350, Rotors>7, XLOGP3>3.5
Synthetic accessibility	4.47

Table S17: ADME-related properties of compound 8h

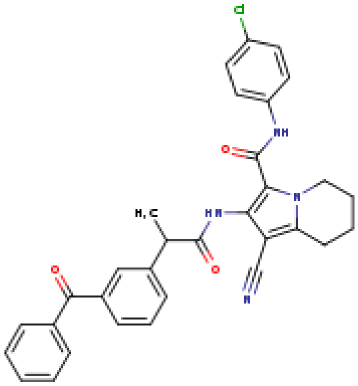

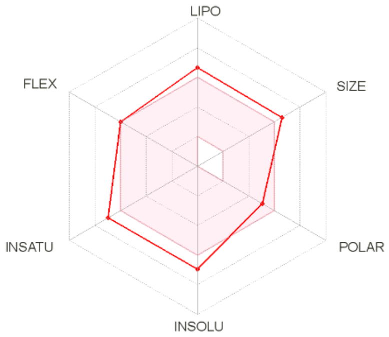

SMILES N#Cc1c(NC(=O)C(c2cccc(c2)C(=O)c2ccccc2)C)c(n2c1CCCC2)C(=O)Nc1ccc(cc1)Cl

Physicochemical Properties	
Formula	C32H27ClN4O3
Molecular weight	551.03 g/mol
Num. heavy atoms	40
Num. arom. heavy atoms	23
Fraction Csp3	0.19
Num. rotatable bonds	9
Num. H-bond acceptors	4
Num. H-bond donors	2
Molar Refractivity	155.58
TPSA	103.99 Å²

Lipophilicity	
Log $P_{o/w}$ (iLOGP)	3.84
Log $P_{o/w}$ (XLOGP3)	6.13
Log $P_{o/w}$ (WLOGP)	6.19
Log $P_{o/w}$ (MLOGP)	3.12
Log $P_{o/w}$ (SILICOS-IT)	6.01
Consensus Log $P_{o/w}$	5.06
Water Solubility	
Log S (ESOL)	-6.95
Solubility	6.19e-05 mg/ml ; 1.12e-07 mol/l
Class	Poorly soluble
Log S (Ali)	-8.10
Solubility	4.42e-06 mg/ml ; 8.02e-09 mol/l
Class	Poorly soluble
Log S (SILICOS-IT)	-10.29
Solubility	2.82e-08 mg/ml ; 5.12e-11 mol/l
Class	Insoluble
Pharmacokinetics	
GI absorption	Low
BBB permeant	No
P-gp substrate	No
CYP1A2 inhibitor	No
CYP2C19 inhibitor	Yes
CYP2C9 inhibitor	Yes
CYP2D6 inhibitor	No
CYP3A4 inhibitor	Yes
Log K_p (skin permeation)	-5.31 cm/s
Druglikeness	
Lipinski	Yes; 1 violation: MW>500
Ghose	No; 3 violations: MW>480, WLOGP>5.6, MR>130
Veber	Yes

Egan	No; 1 violation: WLOGP>5.88
Muegge	No; 1 violation: XLOGP3>5
Bioavailability Score	0.55
Medicinal Chemistry	
PAINS	0 alert
Brenk	0 alert
Leadlikeness	No; 3 violations: MW>350, Rotors>7, XLOGP3>3.5
Synthetic accessibility	4.52

Table S18: ADME-related properties of compound 8i

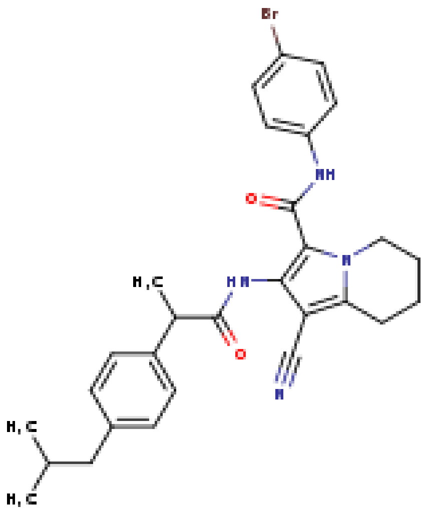

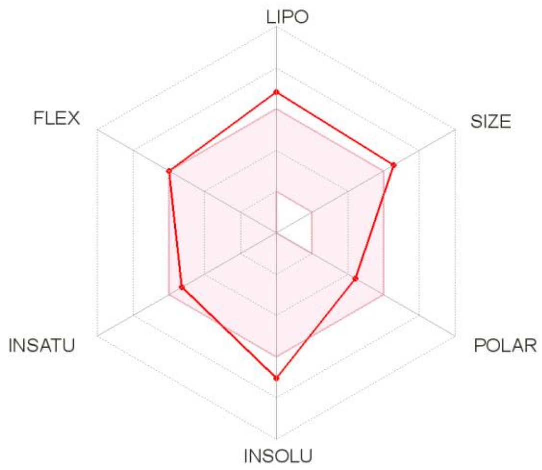

SMILES N#Cc1c(NC(=O)C(c2ccc(cc2)CC(C)C)C)c(n2c1CCCC2)C(=O)Nc1ccc(cc1)Br

Physicochemical Properties	
Formula	C29H31BrN4O2
Molecular weight	547.49 g/mol
Num. heavy atoms	36
Num. arom. heavy atoms	17
Fraction Csp3	0.34
Num. rotatable bonds	9
Num. H-bond acceptors	3

Num. H-bond donors	2
Molar Refractivity	147.78
TPSA	86.92 Å²
Lipophilicity	
Log $P_{o/w}$ (iLOGP)	4.23
Log $P_{o/w}$ (XLOGP3)	6.44
Log $P_{o/w}$ (WLOGP)	6.27
Log $P_{o/w}$ (MLOGP)	3.73
Log $P_{o/w}$ (SILICOS-IT)	6.09
Consensus Log $P_{o/w}$	5.35
Water Solubility	
Log *S* (ESOL)	-7.05
Solubility	4.91e-05 mg/ml ; 8.97e-08 mol/l
Class	Poorly soluble
Log *S* (Ali)	-8.06
Solubility	4.78e-06 mg/ml ; 8.72e-09 mol/l
Class	Poorly soluble
Log *S* (SILICOS-IT)	-9.29
Solubility	2.79e-07 mg/ml ; 5.09e-10 mol/l
Class	Poorly soluble
Pharmacokinetics	
GI absorption	Low
BBB permeant	No
P-gp substrate	Yes
CYP1A2 inhibitor	No
CYP2C19 inhibitor	Yes
CYP2C9 inhibitor	Yes
CYP2D6 inhibitor	Yes
CYP3A4 inhibitor	Yes
Log K_p (skin permeation)	-5.07 cm/s
Druglikeness	

Lipinski	Yes; 1 violation: MW>500
Ghose	No; 3 violations: MW>480, WLOGP>5.6, MR>130
Veber	Yes
Egan	No; 1 violation: WLOGP>5.88
Muegge	No; 1 violation: XLOGP3>5
Bioavailability Score	0.55
Medicinal Chemistry	
PAINS	0 alert
Brenk	0 alert
Leadlikeness	No; 3 violations: MW>350, Rotors>7, XLOGP3>3.5
Synthetic accessibility	4.50
